# Functional and microbiological effects of a microencapsulated probiotic consortium on the ruminal microbiota *in vivo and in vitro* systems

**DOI:** 10.1080/10495398.2025.2547345

**Published:** 2025-08-22

**Authors:** Johanna Marcela Urán Velásquez, Mauricio Agudelo Rendón, Mariana Zapata, Wilyer García Arboleda, Sara Echeverri, Juan Camilo Arroyave Manco, Koen Venema, Juan Vasquez

**Affiliations:** a Research Group in Nutrition and Animal Health, Bialtec S.A.S, Medellín, Colombia; b Mineral Nutrition Research Center, Research Development and Innovation Department, SOMEX S.A.S, Medellin, Colombia; c Beneficial Microbes^®^ Consultancy, Wageningen, the Netherlands

**Keywords:** Functional analysis, microbial community, microencapsulation, physicochemical parameters, probiotic, rumen simulation

## Abstract

Fistulated animals and rumen simulation systems are essential for evaluating the effects of ingredients like probiotics, proposed as sustainable alternatives to growth-promoting antibiotics in ruminant nutrition. This study assessed the impact of microencapsulated probiotics on the structure and functionality of an initial ruminal microbial community using *in vivo* (IVV) and *in vitro* (IVT) systems. The IVT system was inoculated with rumen fluid obtained from the cattle animal used as IVV system. Over time, both systems were analysed for short-chain fatty acid (SCFA) production, microbial composition and functionality using next-generation sequencing. Physicochemical parameters were consistent across both systems and the inoculum, with an increase in propionate concentration observed. Although the microbial composition of IVV and IVT systems was highly similar (Pearson correlation of 0.869), significant differences in B-diversity were noted (*p* value = 0.023). The systems also exhibited high similarity in enzymatic profiles (correlation: 0.971) and metabolic pathways (correlation: 0.938), despite differences in functional B-diversity. Both systems showed increased production of fibrolytic enzymes, enhancing feed efficiency. The use of microencapsulated probiotics induced both taxonomic and functional changes in the initial microbial community of the IVT and IVV systems, which can be linked to the zootechnical effects of using probiotics as additives in ruminal animal nutrition.

## Introduction

Rumen is one of the compartments in the stomach of ruminant animals, including sheep, goats and cows. The rumen harbours a highly complex microbiota, which plays a crucial role in fermentation processes, where microorganisms interact synergistically to degrade fibrous substrates and ferment the resulting substrates into short-chain fatty acids (SCFA), gases and other metabolites. This metabolic activity is fundamental for the nutrition and health of ruminants,^1^ as well as for the sustainable production of meat and milk. Consequently, understanding the ruminal microbiota’s composition and functionality has been a central focus of animal science.^2–4^ Several studies have shown that the composition and functionality of the ruminal microbiota can vary considerably in response to dietary, environmental, and physiological factors.^5^^,^^6^

Among the strategies to modulate this microbiota, probiotics have emerged as a promising tool to enhance the health and performance of ruminants by promoting beneficial microbial communities and inhibiting pathogenic microbes.^7^ However, much of the existing research has focused on freeze-dried probiotics, which are susceptible to degradation in the harsh conditions of the gastrointestinal tract and may not achieve their full potential *in vivo* (IVV). Previous studies have largely overlooked the use of microencapsulated probiotic consortia, which offer several advantages over traditional methods. A recent study by Ramon et al.^8^ explored the use of microencapsulated probiotic microorganisms in ruminant diets and demonstrated substantial improvements in fibre digestibility and dry matter intake. Microencapsulation has emerged as a groundbreaking technology that protects probiotics from gastrointestinal conditions, significantly enhancing their stability, bioavailability, and functional efficacy in the rumen.^9^ Additionally, the global demand for probiotics in animal nutrition continues to rise, with the market valued at USD 5.18 billion in 2023 and projected to keep growing, driven by increasing awareness of animal health, productivity, and the environmental impact of livestock systems.^10^ These trends give relevance to studying the effects of microencapsulated probiotics in ruminal systems.

While previous studies have focused on individual probiotic strains, few have comprehensively explored the functional and structural impact of microencapsulated probiotic consortia in the rumen. Some studies in animals have been focused on the evaluation of the effects of probiotics on the ruminal microbiota, mainly concluding that probiotics stimulate the establishment of a healthy microbial community.^4^^,^^11^ Additionally, *in vitro* (IVT) systems that simulate the ruminal environment have been used to study the effects of different diets, additives and conditions on the microbial community in the rumen,^12^^,^^13^ and few studies have integrated both IVV and IVT systems to comprehensively explore the functional and structural impacts of microencapsulated probiotics across multiple conditions. Despite significant advances in our understanding of the composition and microbiological functionality of the rumen, there are still important gaps in our knowledge, especially regarding the mechanisms of action of probiotics and their effects on enzyme expression and specific metabolic pathways in the rumen.^7^ This gap underscores the need for studies that investigate the functional implications of probiotic supplementation on ruminal metabolism and microbial community structure. Therefore, the ability to simulate, standardize, and manipulate these communities in controlled environments offers unprecedented opportunities for the advancement of ruminal science and the development of innovative nutritional strategies to optimize rumen function and the production of high-quality meat and milk.^2^

The addition of microencapsulated probiotics is expected to enhance ruminal fermentation efficiency by modifying the structure and functionality of the microbial community. Accordingly, the aim of this study was to deepen our understanding of how microencapsulated probiotics (Fortcell Feed^®^) supplementation influences the physicochemical parameters and microbial characteristics of the ruminal microbiome by assessing both functional and structural changes in an IVV system (fistulated cattle animal) and an IVT system (reactors inoculated with ruminal fluid).

## Materials and methods

### *In vivo* system

The IVV system was a crossbred steer (Blanco Orejinegro [BON] breed, a Colombian native cattle breed), approximately 32 months old and weighing about 400 kg, fitted with a rumen fistula and maintained at the Somex Mineral Nutrition Research Center (Cinmex) located in the municipality of El Santuario, Antioquia, Colombia. Due to regulatory constraints that limit the availability of fistulated animals and to avoid inter-animal variability in the direct comparison of IVV and IVT systems, a single fistulated donor was used. The IVV was fed *ad libitum* with King Grass (*Pennisetum purpureum ×* *Pennisetum glaucum*) cut pasture, which was selected due to its nutritional composition being representative of diets commonly used in Colombia, making its degradability of particular interest. Commercial mineral supplement (Nitromin 1, Somex) containing 0.6% microencapsulated probiotics (Fortcell Feed^®^ composed of microencapsulated *Bacillus subtilis* BS-GA28 and *Saccharomyces cerevisiae* CEC01, at a concentration of 1x10^9^ CFU/g) was supplied directly into the rumen via fistula every day until the end of the experiment, with a daily quantity of 100 g/d. The composition of this supplement is commonly used as a reference product in ruminant nutrition in Colombia and contains the microencapsulated probiotics that the study aimed to evaluate. At the start of this study (day 0), 2 L ruminal fluid samples from the IVV were taken to be used as ‘inoculum’ for the IVT system. Sampling of ruminal fluid (45 mL) from this IVV system was done every week for 4 weeks. 40 mL of ‘Inoculum’ and of each sample from IVV system underwent measurements of pH, total solids (TS) and volatile solids (VS, which represent the organic fraction of the solids that can be volatilized upon heating at 550 °C). An aliquot of 3 mL of each sample was stored at −80 °C until processed for SCFA concentration measurement. An aliquot of 1.5 mL of each sample was stored in a 2 mL Eppendorf tube at −80 °C until processed for metagenomic analysis.

### *In vitro* system

The IVT system was a 900 mL volume Rusitec-type reactor with a working volume of 700 mL, maintained at 39 °C with periodic piston flow agitation. The IVT was inoculated with 500 mL of ruminal fluid, 200 mL of McDougall′s buffer and 80 g of ruminal digesta from IVV. The IVT system had 4 replicates (i.e. 4 experimental units). The IVT was fed with a loading rate of 15 g dry King Grass/day using standard Ankom R510 porous bags (Ankom Technology, Macedon, NY), with dimensions of 5 cm × 10 cm with a 50 ± 10-micron porosity. Each bag also contained 1.4 g of The Commercial Nitromin 1 with microencapsulated probiotics. The bags were changed once a day manually. After each change of bag, the reactors were flushed with CO_2_ for 40 s to keep an anaerobic environment. McDougall buffer with pH = 8.4 was continuously fed to maintain the dilution rate *via* a peristaltic pump, maintaining a liquid phase hydraulic retention time (TRH) of 1.5 d. Each week for a period of 4 weeks, a 45 mL sample of effluent from each replicate underwent measurement of pH, TS and VS. Also, each week 3 mL of sample was stored at −80 °C until processed for SCFA concentration measurement. An aliquot of 2 mL of effluent was stored at −80 °C with the same periodicity for evaluation of the microbial community (microbial community analysis was only conducted on samples from one of the replicates). The evaluation for IVV and IVT systems was carried out at the same time.

### Analytical and statistical methods for physicochemical parameters

For TS and VS measurements, the method established in the Standard Methods of the American Public Health Association^14^ was utilized. pH was measured using a pH meter (Thermo Scientific OrionStarTM A221). The SCFA: acetic, propionic, butyric and valeric acid, were quantified by high-performance liquid chromatography (HPLC) using an Agilent 1200 system equipped with an ICSep COREGEL-87H3 column. The mobile phase consisted of 0.01 N sulphuric acid, and its flow rate was maintained at of 0.6 mL/min. The column’s temperature was maintained at 80 °C, and an Infrared (IR) detector was employed at 55 °C. The volume of the sample injected was 20 µL. All statistical comparisons were conducted using the SPSS software, with a significance level set at 0.05.

### DNA extraction and sequencing

DNA from samples was isolated using the NORGEN Stool DNA Isolation Kit, according to the manufacturer’s protocol. The DNA samples were quantified using NanoDrop 2000/2000c (Thermo Scientific, Waltham, MA). The DNA libraries were prepared using the Nextera XT DNA Library Preparation Kit (Illumina, San Diego, CA) and IDT Unique Dual Indexes with total DNA input of 1 ng. Genomic DNA was fragmented using a proportional amount of Illumina Nextera XT fragmentation enzyme. Unique dual indexes were added to each sample followed by 12 cycles of PCR to construct libraries. The DNA libraries were purified using AMpure magnetic Beads (Beckman Coulter, Brea, CA) and eluted in QIAGEN EB buffer. The DNA libraries were quantified using Qubit 4 fluorometer and Qubit dsDNA HS Assay Kit. Libraries were then sequenced on Illumina NovaSeq platform 2 × 150 bp.

### Bioinformatics and statistical analysis for microbial community

A high-performance k-mer algorithm was used to analyse microbial NGS reads, mapping them to curated reference databases (genomes, virulence, antimicrobial resistance markers). Taxonomic classification and relative abundance were determined using a phylogeny tree and variable length k-mer fingerprints. To exclude false positives, results were filtered using a threshold based on internal statistical scores derived from diverse metagenomes. Alpha diversity was calculated using Chao1 (used to estimate species richness), Simpson (used to quantify the diversity of a community) and Shannon (used to quantify species richness and evenness in a community) indices and compared using Wilcoxon tests. Beta diversity was assessed using Bray–Curtis similarity, with statistical differences evaluated using PERMANOVA (9999 permutations). Principal coordinate analysis (PCoA) was performed.

### Bioinformatics and statistics for functional analysis

Adapter trimming, QC and preprocessing of reads were done using BBduk.^15^ Reads were mapped against the UniRef90 protein database, clustering sequences with ≥90% identity.^16^ Metagenomic reads were mapped to gene sequences, and gene family abundances were estimated as described by.^17^ Gene families were annotated to MetaCyc reactions and further categorized into enzyme classes (Enzyme Commission, Pfam, CAZy and GO Terms) to assess gene function in the community.^18^ Abundance values were normalized using total sum scaling (TSS) to produce ‘Copies per million’ (CPM) units. Pearson correlation indexes were calculated to compare metabolic pathways and enzymes between the IVV and IVT systems and to correlate SCFA production with enzyme presence. Statistical differences in metabolic pathways were calculated using PERMANOVA (9999 permutations), and PCoA of CPM was performed.

## Results

### Physicochemical results

**Figure 1.**
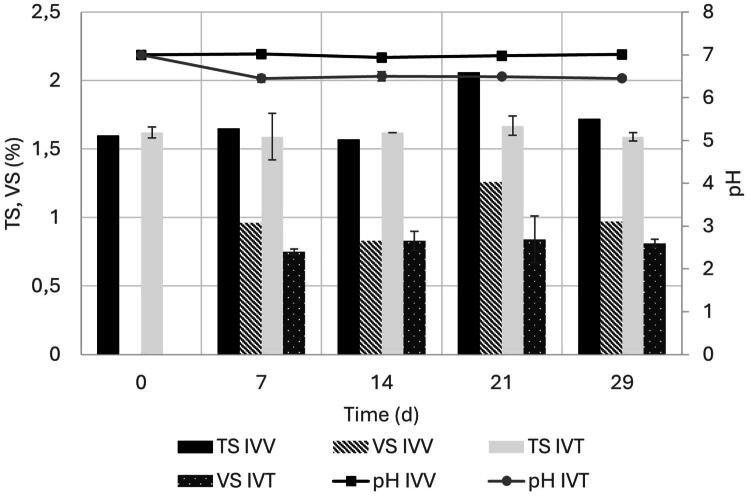
Physicochemical parameters over time for IVV and IVT systems. Values at day 0 correspond to the parameters of the inoculum. TS: total solids; VS: volatile solids. Error bars represent one standard deviation above and below the mean, calculated from the four replicates for IVT.

The pH in IVV remained constant at a value close to 7.0 during the whole experiment, while the pH in the IVT system decreased from 7.0 to a value close to 6.5 during the first 7 d of the experiment and remained constant around that value for the rest of the experiment. In terms of TS and VS, differences were observed only on day 21, where higher values of TS and VS were recorded in the IVV system. Values of TS and VS in the IVT system remained constant at around 1.6% and 0.8%, respectively, while in the IVV system, TS values varied between 1.6 and 2.1% and VS values varied between 0.7 and 1.3% (Figure 1).

**Figure 2.**
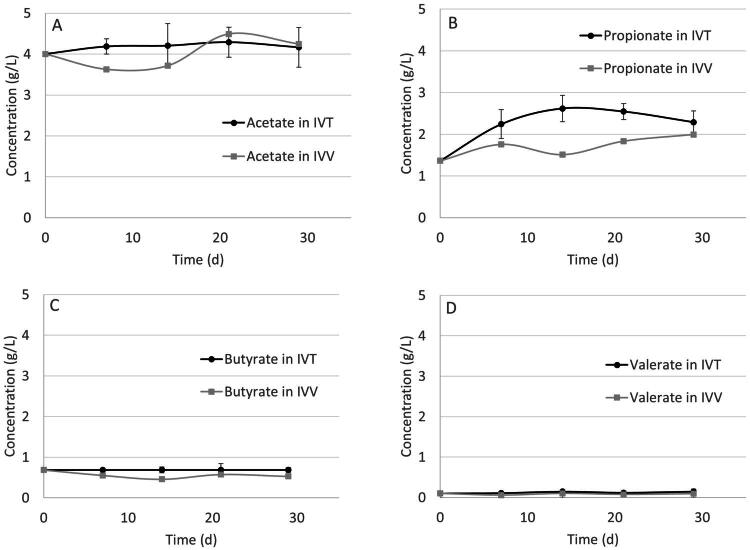
Evolution of SCFA for IVV and IVT systems. (A) Acetate, (B) propionate, (C) butyrate, (D) valerate. Values at day 0 correspond to the inoculum. Error bars represent one standard deviation above and below the mean, calculated from the four replicates for IVT.

The behaviour of the IVT systems shows less variation over time than the IVV regarding SCFA concentration. In the IVV system, acetate values remained close to 4.1 g/L, propionate concentrations varies between 1.3 and 2.2 g/L, butyrate values remained close to 0.7 g/L and valerate values remained close to 0.1 g/L. In the IVT system, acetate values varied between 3.6 and 4.4 g/L, propionate concentrations varies between 1.3 and 2.0 g/L, butyrate values remained close to 0.7 g/L and valerate values remained close to 0.1 g/L. The acetate to propionate ratio on the rumen used for the inoculum before the experiment started was 2.9. During the experiment, this ratio was found to be on average 2.27 ± 0.203 for the IVV system and 1.74 ± 0.121 for the IVT system. A difference was observed at one evaluation point (day 14) in the concentrations of propionate and butyrate between the systems; however, this difference was not sustained over time (Figure 2).

### Microbiological analysis

**Figure 3.**
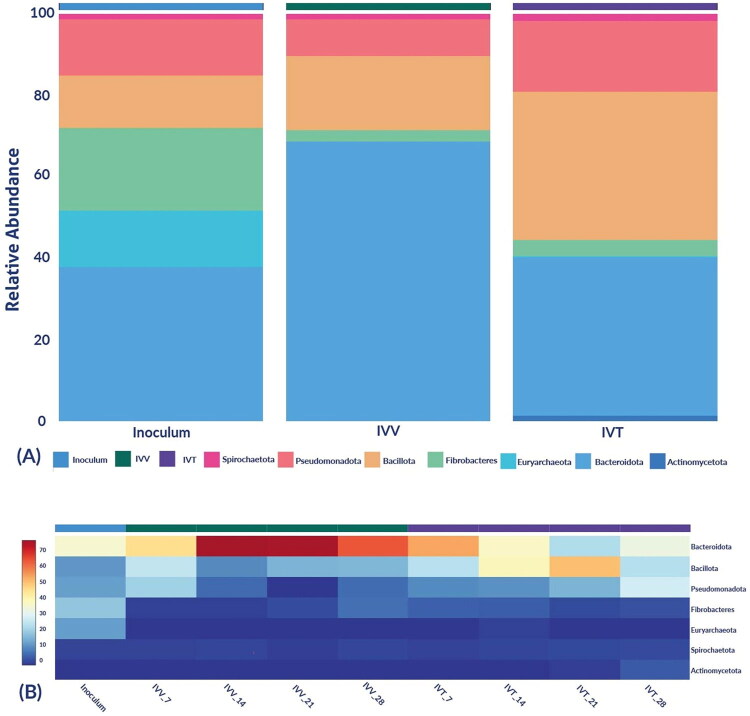
Relative abundance of microbial composition at the phylum level. (a) Bar distribution of phyla for the inoculum and the *in vivo* (IVV) and *in vitro* (IVT) systems. (b) Heatmap of phyla for the inoculum and each of the analysed samples from the *in vivo* (IVV) and *in vitro* (IVT) systems in the days 7, 14, 21 and 28).

In all samples evaluated, 7 phyla were identified: *Pseudomonadota, Bacteroidota, Bacillota, Actinomycetota, Spirochaetota, Fibrobacteres* and *Euryarchaeota*. In the inoculum from which these systems were assembled, all previously mentioned phyla were identified: *Bacteroidota, Fibrobacteres, Pseudomonadota, Euryarchaeota, Bacillota* and *Spirochaetota*, with abundances of 37.9%, 20.4%, 13.8%, 13.7%, 12.8% and 1.4%, respectively, except for the *Actinomycetota* phylum, which was only identified in the IVT system (Figure 3(a)). When comparing the evaluated systems: IVV and IVT, the most abundant phylum in all these systems was *Bacteroidota* with 68.58% and 38.92% respectively, with statistically significant differences (*p* < 0.043), followed by the *Bacillota* phylum with abundances of 18.14% and 36.27%, respectively, without significant differences.

**Figure 4.**
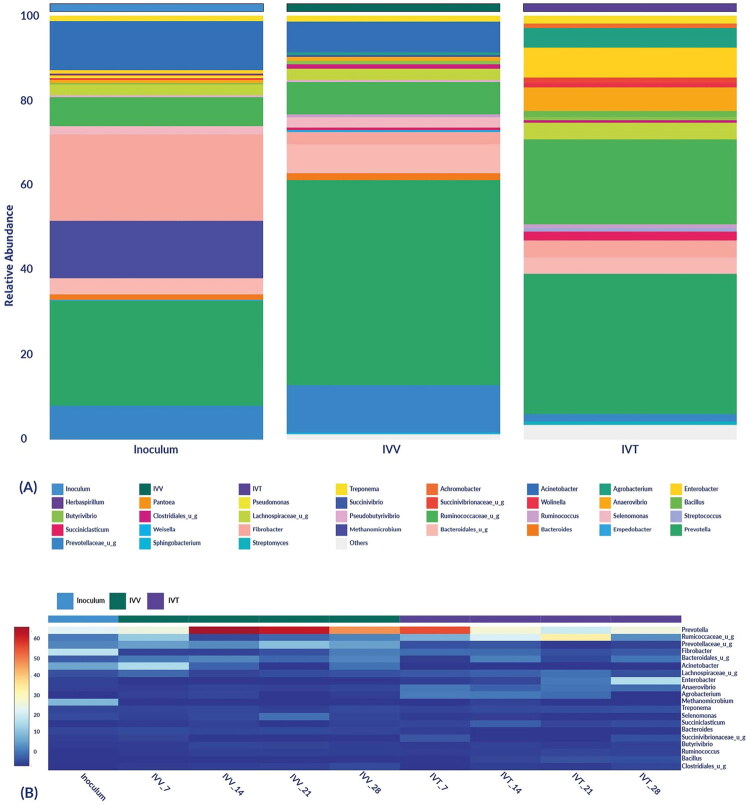
Relative abundance of microbial composition at the genus level. a) Bar distribution of the top 20 most abundant genera for the inoculum and the *in vivo* (IVV) and *in vitro* (IVT) systems. b) Heatmap of the top 20 most abundant genera for the inoculum and each analysed sample from the *in vivo* (IVV) and *in vitro* (IVT) systems in the days 7, 14, 21 and 28).

Regarding the microbial composition at the genus level, a total of 43 genera were identified, with *Prevotella* spp. being the most abundant both in the inoculum and in the two systems, IVV and IVT, with abundances of 24.79%, 48.44% and 33.07%, respectively. Significant differences between the systems were observed in *Agrobacterium* spp., *Anaerovibrio* spp. and *Succiniclasticum* spp. (*p* < 0.05, *p* < 0.021 and *p* < 0.034, respectively) (Figure 4).

According to the diversity analyses (Table 1), IVV and IVT have similar diversity according to the Chao1 and Simpson indices, but IVT shows higher species evenness according to the Shannon index. On the other hand, the inoculum sample presented lower species richness estimation according to the Chao1 index and higher species dominance according to the Simpson index. Despite these observed differences, none of them were statistically significant, as confirmed by Wilcoxon tests where all the p-values were found to be higher than 0.05 (Table 1).

**Table 1.** Alpha diversity indices and statistical comparisons of alpha diversities between systems.

	Alpha diversity index value^a^				*p* value for the comparison^b^		
Sample	Chao	Simpson	Shannon	Comparison	Chao	Simpson	Shannon
Inoculum	30	0.91	3.92	Inoculum-IVV	1	0.48	0.48
IVV	30	0.86	3.59	Inoculum-IVT	0.72	0.48	0.48
IVT	32	0.87	3.81	IVV-IVT	0.56	0.38	0.38

^a^Alpha diversity index values for the inoculum and the evaluated *in vivo* (IVV) and *in vitro* (IVT) systems. Indices for IVV and IVT were calculated as the average of all time points.

^b^*p* Values from Wilcoxon tests comparing diversity between systems.

### Functional analysis

**Figure 5.**
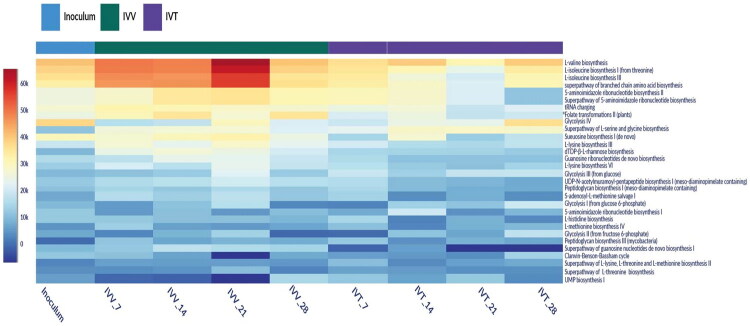
Heatmap of the top 30 most abundant metabolic pathways based on copies per million (CPM) for the inoculum and each analysed sample from the *in vivo* (IVV) and *in vitro* (IVT) systems in the days 7, 14, 21 and 28. Colours indicate abundance, with red indicating the highest abundance in CPM and dark blue indicating the lowest abundance.

A total of 134 metabolic pathways were identified in all the samples. The composition was similar, both in the inoculum and in the samples from the IVV and IVT systems. The most abundant metabolic pathways for all systems in question (inoculum, IVV, and IVT) were L-valine biosynthesis with 45,394, 55,231 and 41,537 CPM, respectively, and L-isoleucine biosynthesis I with 43,072, 53,628 and 36,300 CPM, respectively (Figure 5).

Between the two evaluated systems, IVV *vs.* IVT, statistically significant differences were observed in the abundance of CPM for the following metabolic pathways: super pathway of branched-chain amino acid biosynthesis (*p* < 0.021), coenzyme A biosynthesis I (prokaryotic) (*p* < 0.05), dTDP-β-L-rhamnose biosynthesis (*p* < 0.021), L-isoleucine biosynthesis I (from threonine) (*p* < 0.021), L-lysine biosynthesis III (*p* < 0.021), folate transformations II (plants) (*p* < .021), L-lysine biosynthesis VI (*p* < 0.021), L-isoleucine biosynthesis III (*p* < 0.021), 5-aminoimidazole ribonucleotide biosynthesis II (*p* < 0.043), superpathway of 5-aminoimidazole ribonucleotide biosynthesis (*p* < 0.043), UDP-N-acetylmuramoyl-pentapeptide biosynthesis I (meso-diaminopimelate containing) (*p* < 0.043), adenine and adenosine salvage III (*p* < 0.021), guanosine ribonucleotides de novo biosynthesis (*p* < 0.021), tRNA charging (*p* < 0.021), L-valine biosynthesis (*p* < 0.021). It is important to mention that of all the metabolic pathways obtained in the samples, only the mixed acid fermentation pathway is directly involved in cellulolytic processes, as it is implicated in the fermentation of cellulolytic products into organic acids such as lactic acid, acetic acid, and succinic acid, which are products of cellulose degradation by microorganisms in the rumen.

**Figure 6.**
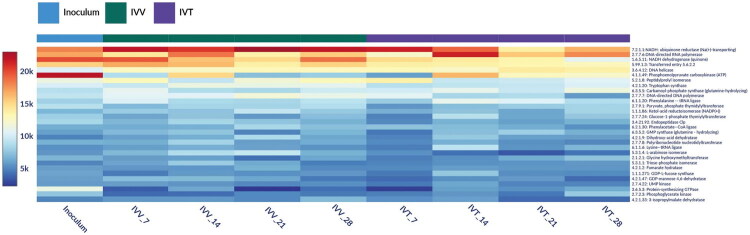
Heatmap of the top 30 most abundant enzymes based on copies per million (CPM) for the inoculum and each analysed sample from the *in vivo* (IVV) and *in vitro* (IVT) systems in the days 7, 14, 21 and 28. Colours indicate abundance, with red indicating the highest abundance in CPM and dark blue indicating the lowest abundance.

**Figure 7.**
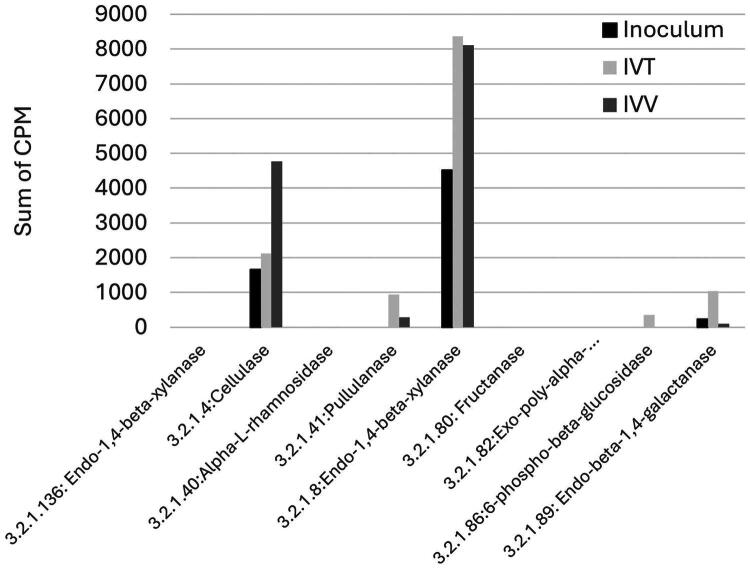
Main cellulolytic enzymes found in the functional analysis for the inoculum and the IVV and IVT systems. CPM: copies per million.

On the other hand, approximately 813 enzymes were identified in the analysed samples. The most abundant enzymes in the inoculum and in the two systems (IVV and IVT), were those related to biological processes such as DNA replication (Figure 6). Nevertheless, a deeper analysis of cellulolytic enzymes that are important in the ruminal environment for the degradation of grass, showed that in general, their CPM were higher in the IVV and IVT systems compared to the inoculum (Figure 7). This was especially evident for the Cellulase, pullulanase and endo-1,4-beta-xyalanase enzymes that were found in the IVV and IVT systems up to twice their presence in the inoculum.

**Figure 8.**
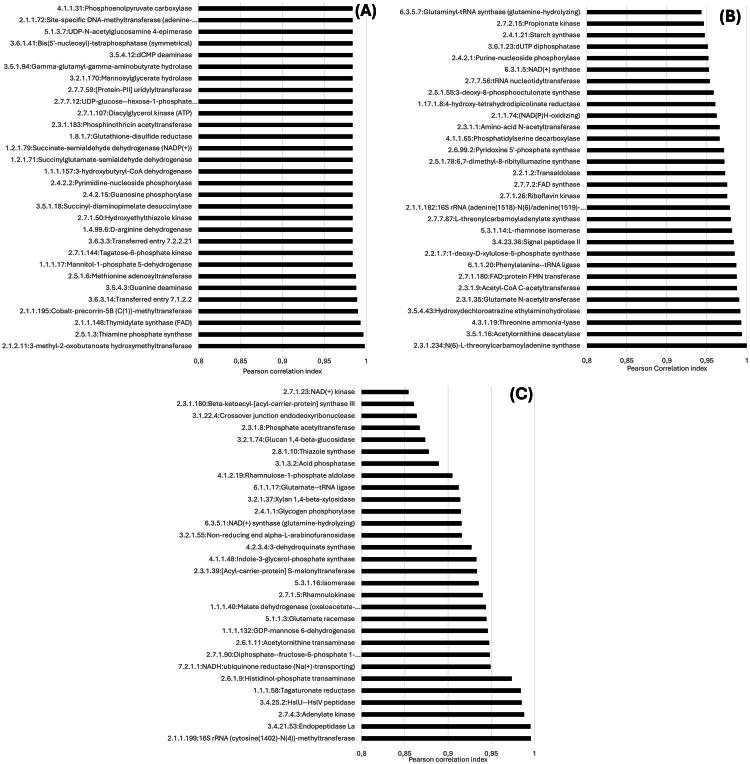
Most correlated enzymes for the main volatile fatty acids observed in the *in vivo* (IVV) and *in vitro* (IVT) systems. (A) Acetate; (B) Propionate; (C) Butyrate.

The relation between SCFA and enzymes was analysed. Figure 8 shows the enzymes that had the highest positive correlation with the concentration of each of the main SCFA found in this study (acetate, propionate and butyrate). For visual purposes, only the 30 of the most correlated enzymes are shown, nevertheless, the correlation values for all the enzymes identified in the functional analysis are presented in Supplementary material. The number of enzymes with a high correlation index (higher than 0.75) were 203 for acetate, 109 for propionate and 60 for butyrate. From those, 6 enzymes correlated highly with all the three SCFA at the same time, the enzymes were pectinesterase, demethylmenaquinone methyltransferase, feruloyl esterase, purine-nucleoside phosphorylase, dehydroquinate synthase and glycogen phosphorylase (Figure 8).

### Similarity analysis

To approach the similarity between the inoculum IVV and IVT systems, beta diversity and Pearson correlation analyses were conducted based on their microbial and functional composition, i.e. metabolic pathways and enzymes.

**Figure 9.**
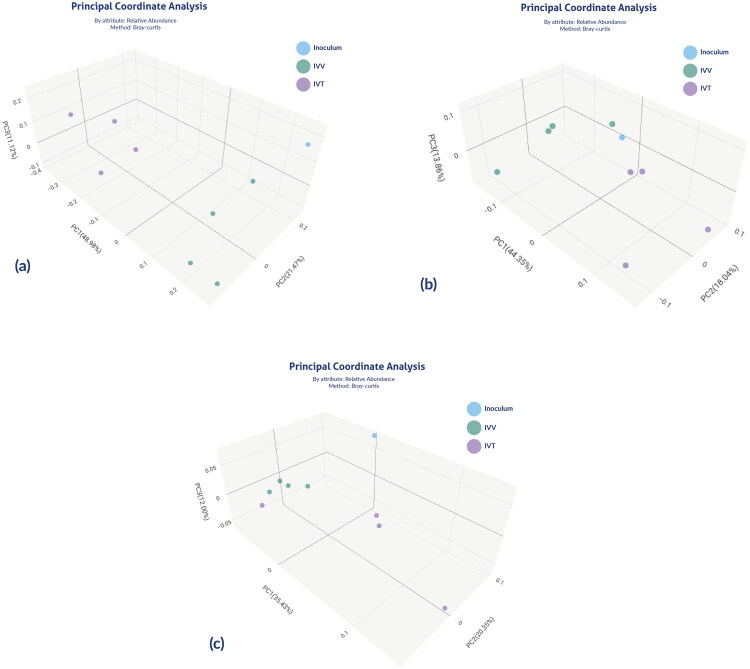
Principal coordinate analysis (PCoA) plot with Bray–Curtis dissimilarity. Plots illustrating distances between communities, the inoculum and the IVV and IVT systems, in all individual samples (*n* = 8), for (a) microbial community, (b) metabolic pathways, (c) enzymes composition.

Beta diversity analyses based on microbial composition suggest significant differences among all analysed samples (*p* = 0.003), as well as between the IVV and IVT samples specifically (*p* < 0.023). Similarly, significant differences in beta diversity for metabolic pathways and enzymes composition were observed between the IVV and IVT systems (*p* < 0.02) and (*p* < 0.027), respectively (Figure 9).

Moreover, Pearson correlation allows us to evaluate how much treatments resemble each other based on the values of microbial and functional composition, where values close to 1 indicate high similarity between the evaluated groups. Based on this, the Pearson correlation coefficients for microbial composition, metabolic pathways and enzyme profiles are presented (Table 2). An average correlation coefficient of 0.869 at the microbiological level between the IVV and IVT systems was observed. Lower correlations were found between the inoculum and IVT (*r* = 0.673), and between the inoculum and IVV (*r* = 0.756). Similarly, at the metabolic and enzymatic levels, greater similarity between the IVV and IVT systems was evidenced with correlation coefficients of 0.93 and 0.971, respectively (Table 2).

**Table 2.** Pearson correlation coefficients between the inoculum, *in vivo* (IVV) and in vitro (IVT) systems.

	Pearson correlation coefficient		
	Microbial composition	Pathways	Enzyme composition
Inoculum-IVT	0.673	0.924	0.942
Inoculum-IVV	0.756	0.916	0.935
IVT-IVV	0.869	0.938	0.971

## Discussion

The results of this study show that the physicochemical and microbiological parameters of the rumen fluid, collected at the beginning of the experiment from the IVV steer and used as inoculum to initiate the IVT system, underwent several changes both in the IVV and the IVT systems after the addition of the microencapsulated probiotics. This demonstrates how microencapsulation enhances probiotic stability and effectiveness in both systems, offering a novel approach to improving rumen function and animal growth. These findings support previous research indicating that probiotics can enhance zootechnical performance in ruminants by improving nutrient utilization.^19–22^ This was further described by Ramon et al., who assessed the effects of using microencapsulated probiotic microorganisms in both IVV and IVT systems, demonstrating significant improvements in fibre and dry matter digestibility of pastures, achieving up to an 11% increase in neutral detergent fibre (NDF) and acid detergent fibre (ADF) digestibility, thereby increasing weight gain in animals by up to 115 g/animal/day.^8^

The stability of physicochemical parameters such as pH, TS, and VS in the IVV and IVT systems throughout the experiment of this study underscores the robustness of IVT systems in maintaining rumen-like conditions. The usefulness of IVT systems to properly simulate ruminal environments and conduct studies that could predict what happens IVV has also been confirmed in other studies.^23^^,^^24^ The slight decrease in pH in the IVT system initially, which stabilized around 6.5, may reflect the influence of several factors such as the adaptation of the microbial community to the IVT environment (including a well-documented reduction in protozoa in IVT systems compared to IVV systems), the absence of active absorption of acids as it occurs in the IVV animals and the differences in production and elimination mechanisms of gases (mainly CO_2_) between both systems.^25^^,^^26^ The fact that the pH in the IVT system was consistently lower than the pH of the buffer used in the experiments reflects the proper action of the microbial community that lowers the pH due to the production of SCFA. The pH values observed in both systems over time are close to what has been reported for the pH values of ruminal environments.^27^ The generally consistent TS and VS values between systems indicate that the probiotic supplementation did not adversely affect the overall physicochemical environment of the rumen.

Despite that the concentration of most SCFA did not change significantly over time, an increase was observed in propionate concentration in both IVV and IVT systems compared to the inoculum, as well as a decrease in the acetate to propionate ratio. This suggests that there was enhanced fermentation efficiency, a critical factor for improved feed conversion and animal growth. Propionate is a key gluconeogenic precursor, and its increased production is often associated with better feed efficiency and weight gain in ruminants. These findings are consistent with studies by^28^ and^29^ who found a correlation between increased propionate production and improved feed efficiency and methane reduction which benefits both the environment and the animal’s energy efficiency. This finding is supported by the significant correlations observed between the relative abundance of certain enzymes and SCFA production, indicating that the probiotic-induced changes in microbial enzyme activity are directly linked to improved fermentation outcomes. It was also observed that six enzymes found in the functional analysis highly correlated with the three main SCFA observed in both systems (acetate, propionate and butyrate). Those enzymes can be of interest for further studies of rumen metabolism and SCFA production in such environments.

To date, the number of studies describing the changes occurring in rumen microbiome composition and functionality is limited, and even more so in IVT systems, where data comparison between different systems can demonstrate the ability to adapt a community to various conditions. In this study, bacteria were the predominant kingdom identified in all samples, consistent with their critical role in feed biopolymer degradation and fermentation processes.^30^^,^^31^ Notably, the phyla *Bacteroidetes* and *Bacillota*, key players in fibre degradation, were found to be abundant, which supports the hypothesis that probiotics can enhance fibre digestion, thereby improving zootechnical performance.^32^ In addition, the genus *Prevotella* spp. utilizes starch and proteins to produce succinate and acetate and, as in our study, is one of the most abundant genera in the rumen of cows^33^^,^^34^

The high correlation (0.869) in microbial composition between IVV and IVT systems, despite significant differences in beta diversity, suggests that while the overall microbial community structure remained similar, specific taxa varied between the systems. The presence of unique genera in each system and the inoculum reflects the adaptability and specificity of the microbial communities to their respective environments. Also, the high similarity in enzymatic profiles and metabolic pathways between IVV and IVT systems (correlations of 0.971 and 0.938, respectively) indicates that probiotic treatment effectively promoted similar functional outcomes in both environments. The significant increase in fibrolytic enzyme production in both systems, particularly enzymes like cellulase, pullulanase and endo-1,4-beta-xylanase, underscores the role of probiotics in enhancing the breakdown of complex plant fibres and supports their use for better feed efficiency.

## Conclusion

This study offers important insights into the effects of probiotics, such as Fortcell Feed^®^, on ruminal microbiota composition, enzyme production and SCFA metabolism. Following probiotic supplementation, an increase in propionate concentration and a higher abundance of genes associated to fibrolytic enzymes were observed in the ruminal microbiome. These findings suggest that supplemented probiotics, as a sustainable alternative to traditional growth promoters, enhance ruminal fermentation efficiency and nutrient utilization, thereby improving zootechnical performance in ruminants. Future experimental designs should carefully consider control conditions that include the impact of time on the ruminal microbiome’s structure and functionality. Given the high degree of similarity observed between the IVV and IVT systems, the use of IVT models offer a valuable and ethical alternative for further investigation of the specific metabolic pathways and enzymes involved in probiotic-induced improvements, ultimately contributing to the optimization of zootechnical parameters in cattle.

## Supplementary Material

Supplementary material Enzymes with positive correlation indexes.pdf
