# Supplementary material for: Functional and microbiological effects of a microencapsulated probiotic consortium on the ruminal microbiota in vivo and in vitro systems
Source: Anim Biotechnol. 2025 Aug 22;36(1):2547345. doi: 10.1080/10495398.2025.2547345 (PMC12674296; doi:10.1080/10495398.2025.2547345)
Supplement: Supplementary material Enzymes with positive correlation indexes.pdf [file LABT_A_2547345_SM2722.pdf]

System	SCFA	Enzyme	Correlation index
IVT	Propionic Ac [g/L]	2.3.1.234:N(6)-L-threonylcarbamoyladenine synthase	0,999715137
IVV	Acetic Ac [g/L]	2.1.2.11:3-methyl-2-oxobutanoate hydroxymethyltransferase	0,998058926
IVT	Acetic Ac [g/L]	2.5.1.3:Thiamine phosphate synthase	0,996473103
IVV	Butyric Ac [g/L]	2.1.1.199:16S rRNA (cytosine(1402)-N(4))-methyltransferase	0,99575259
IVV	Butyric Ac [g/L]	3.4.21.53:Endopeptidase La	0,995335406
IVT	Propionic Ac [g/L]	3.5.1.16:Acetylornithine deacetylase	0,99385866
IVT	Propionic Ac [g/L]	4.3.1.19:Threonine ammonia-lyase	0,993006278
IVV	Acetic Ac [g/L]	2.1.1.148:Thymidylate synthase (FAD)	0,992982035
IVT	Propionic Ac [g/L]	3.5.4.43:Hydroxydechloroatrazine ethylaminohydrolase	0,991722024
IVT	Propionic Ac [g/L]	2.3.1.35:Glutamate N-acetyltransferase	0,990450785
IVV	Acetic Ac [g/L]	2.1.1.195:Cobalt-precorrin-5B (C(1))-methyltransferase	0,990098729
IVV	Acetic Ac [g/L]	3.6.3.14:Transferred entry 7.1.2.2	0,989187328
IVV	Butyric Ac [g/L]	2.7.4.3:Adenylate kinase	0,988049793
IVV	Acetic Ac [g/L]	3.5.4.3:Guanine deaminase	0,987936765
IVT	Propionic Ac [g/L]	2.3.1.9:Acetyl-CoA C-acetyltransferase	0,987662529
IVV	Acetic Ac [g/L]	2.5.1.6:Methionine adenosyltransferase	0,987527812
IVV	Propionic Ac [g/L]	2.7.1.180:FAD:protein FMN transferase	0,987360788
IVT	Propionic Ac [g/L]	6.1.1.20:Phenylalanine--tRNA ligase	0,986780484
IVV	Butyric Ac [g/L]	3.4.25.2:HslU--HslV peptidase	0,985122183
IVV	Propionic Ac [g/L]	2.2.1.7:1-deoxy-D-xylulose-5-phosphate synthase	0,984745372
IVV	Butyric Ac [g/L]	1.1.1.58:Tagaturonate reductase	0,984149101
IVT	Acetic Ac [g/L]	1.1.1.17:Mannitol-1-phosphate 5-dehydrogenase	0,983641027
IVT	Acetic Ac [g/L]	2.7.1.144:Tagatose-6-phosphate kinase	0,983641027
IVT	Acetic Ac [g/L]	3.6.3.3:Transferred entry 7.2.2.21	0,983641027
IVT	Acetic Ac [g/L]	1.4.99.6:D-arginine dehydrogenase	0,983641027
IVT	Acetic Ac [g/L]	2.7.1.50:Hydroxyethylthiazole kinase	0,983641027
IVT	Acetic Ac [g/L]	3.5.1.18:Succinyl-diaminopimelate desuccinylase	0,983641027
IVT	Acetic Ac [g/L]	2.4.2.15:Guanosine phosphorylase	0,983641027
IVT	Acetic Ac [g/L]	2.4.2.2:Pyrimidine-nucleoside phosphorylase	0,983641027
IVT	Acetic Ac [g/L]	1.1.1.157:3-hydroxybutyryl-CoA dehydrogenase	0,983641027
IVT	Acetic Ac [g/L]	1.2.1.71:Succinylglutamate-semialdehyde dehydrogenase	0,983641027
IVT	Acetic Ac [g/L]	1.2.1.79:Succinate-semialdehyde dehydrogenase (NADP(+))	0,983641027
IVT	Acetic Ac [g/L]	1.8.1.7:Glutathione-disulfide reductase	0,983641027
IVT	Acetic Ac [g/L]	2.3.1.183:Phosphinothricin acetyltransferase	0,983641027
IVT	Acetic Ac [g/L]	2.7.1.107:Diacylglycerol kinase (ATP)	0,983641027
IVT	Acetic Ac [g/L]	2.7.7.12:UDP-glucose--hexose-1-phosphate uridylyltransferase	0,983641027
IVT	Acetic Ac [g/L]	2.7.7.59:[Protein-PII] uridylyltransferase	0,983641027
IVT	Acetic Ac [g/L]	3.2.1.170:Mannosylglycerate hydrolase	0,983641027
IVT	Acetic Ac [g/L]	3.5.1.94:Gamma-glutamyl-gamma-aminobutyrate hydrolase	0,983641027
IVT	Acetic Ac [g/L]	3.5.4.12:dCMP deaminase	0,983641027
IVT	Acetic Ac [g/L]	3.6.1.41:Bis(5'-nucleosyl)-tetraphosphatase (symmetrical)	0,983641027
IVT	Acetic Ac [g/L]	5.1.3.7:UDP-N-acetylglucosamine 4-epimerase	0,983641027
IVT	Acetic Ac [g/L]	2.1.1.72:Site-specific DNA-methyltransferase (adenine-specific)	0,983641027
IVT	Acetic Ac [g/L]	4.1.1.31:Phosphoenolpyruvate carboxylase	0,983641027
IVT	Acetic Ac [g/L]	5.1.3.23:UDP-2,3-diacetamido-2,3-dideoxyglucuronic acid 2-epimerase	0,983641027
IVT	Acetic Ac [g/L]	1.2.7.7:3-methyl-2-oxobutanoate dehydrogenase (ferredoxin)	0,983641027
IVT	Acetic Ac [g/L]	1.7.1.4:Nitrite reductase (NAD(P)H)	0,983641027
IVT	Acetic Ac [g/L]	2.1.1.171:16S rRNA (guanine(966)-N(2))-methyltransferase	0,983641027
IVT	Acetic Ac [g/L]	2.1.1.173:23S rRNA (guanine(2445)-N(2))-methyltransferase	0,983641027
IVT	Acetic Ac [g/L]	2.1.1.35:tRNA (uracil(54)-C(5))-methyltransferase	0,983641027
IVT	Acetic Ac [g/L]	2.3.1.118:N-hydroxyarylamine O-acetyltransferase	0,983641027
IVT	Acetic Ac [g/L]	2.7.14.1:Protein arginine kinase	0,983641027
IVT	Acetic Ac [g/L]	2.7.8.5:CDP-diacylglycerol--glycerol-3-phosphate 1-phosphatidyltransferase	0,983641027
IVT	Acetic Ac [g/L]	2.7.9.3:Selenide, water dikinase	0,983641027
IVT	Acetic Ac [g/L]	3.4.13.9:Xaa-Pro dipeptidase	0,983641027
IVT	Acetic Ac [g/L]	3.6.3.19:Transferred entry 7.5.2.1	0,983641027
IVT	Acetic Ac [g/L]	4.1.3.1:Isocitrate lyase	0,983641027
IVT	Acetic Ac [g/L]	4.2.1.42:Galactarate dehydratase	0,983641027
IVT	Acetic Ac [g/L]	4.2.2.n2:Peptidoglycan lytic endotransglycosylase	0,983641027
IVT	Acetic Ac [g/L]	4.99.1.12:Pyridinium-3,5-bisthiocarboxylic acid mononucleotide nickel chelatase	0,983641027
IVT	Acetic Ac [g/L]	5.4.99.61:Precorrin-8X methylmutase	0,983641027
IVT	Acetic Ac [g/L]	6.2.1.17:Propionate--CoA ligase	0,983641027
IVT	Acetic Ac [g/L]	3.6.1.7:Acylphosphatase	0,983641027
IVT	Acetic Ac [g/L]	1.11.1.1:NADH peroxidase	0,983641027
IVT	Acetic Ac [g/L]	1.17.4.1:Ribonucleoside-diphosphate reductase	0,983641027
IVT	Acetic Ac [g/L]	2.6.1.13:Ornithine aminotransferase	0,983641027
IVT	Acetic Ac [g/L]	1.2.1.39:Phenylacetaldehyde dehydrogenase	0,983641027
IVT	Acetic Ac [g/L]	1.4.1.14:Glutamate synthase (NADH)	0,983641027
IVT	Acetic Ac [g/L]	1.4.3.5:Pyridoxal 5'-phosphate synthase	0,983641027
IVT	Acetic Ac [g/L]	2.1.1.186:23S rRNA (cytidine(2498)-2'-O)-methyltransferase	0,983641027
IVT	Acetic Ac [g/L]	2.1.1.190:23S rRNA (uracil(1939)-C(5))-methyltransferase	0,983641027
IVT	Acetic Ac [g/L]	2.1.1.264:23S rRNA (guanine(2069)-N(7))-methyltransferase	0,983641027
IVT	Acetic Ac [g/L]	3.1.21.7:Deoxyribonuclease V	0,983641027
IVT	Acetic Ac [g/L]	4.3.1.2:Methylaspartate ammonia-lyase	0,983641027
IVT	Acetic Ac [g/L]	6.3.1.8:Glutathionylspermidine synthase	0,983641027
IVT	Acetic Ac [g/L]	7.1.1.1:Proton-translocating NAD(P)(+) transhydrogenase	0,983641027
IVT	Acetic Ac [g/L]	1.1.1.6:Glycerol dehydrogenase	0,983641027
IVT	Acetic Ac [g/L]	1.11.1.6:Catalase	0,983641027
IVT	Acetic Ac [g/L]	3.1.7.2:Guanosine-3',5'-bis(diphosphate) 3'-diphosphatase	0,983641027
IVT	Acetic Ac [g/L]	3.2.1.70:Glucan 1,6-alpha-glucosidase	0,983641027
IVV	Propionic Ac [g/L]	3.4.23.36:Signal peptidase II	0,983541013
IVT	Propionic Ac [g/L]	5.3.1.14:L-rhamnose isomerase	0,981549019

IVT	Propionic Ac [g/L]	2.7.7.87:L-threonylcarbamoyladenylate synthase	0,979732374
IVT	Acetic Ac [g/L]	3.1.3.1:Alkaline phosphatase	0,979336131
IVV	Propionic Ac [g/L]	2.1.1.182:16S rRNA (adenine(1518)-N(6)/adenine(1519)-N(6))-dimethyltransferase	0,978848377
IVV	Acetic Ac [g/L]	2.4.2.21:Nicotinate-nucleotide--dimethylbenzimidazole phosphoribosyltransferase	0,976093859
IVV	Propionic Ac [g/L]	2.7.1.26:Riboflavin kinase	0,975439018
IVV	Propionic Ac [g/L]	2.7.7.2:FAD synthase	0,975439018
IVV	Acetic Ac [g/L]	2.7.2.3:Phosphoglycerate kinase	0,974162399
IVV	Acetic Ac [g/L]	1.3.3.4:Protoporphyrinogen oxidase	0,973903324
IVV	Butyric Ac [g/L]	2.6.1.9:Histidinol-phosphate transaminase	0,973585411
IVT	Propionic Ac [g/L]	2.2.1.2:Transaldolase	0,972866686
IVV	Acetic Ac [g/L]	1.17.7.3:(E)-4-hydroxy-3-methylbut-2-enyl-diphosphate synthase (flavodoxin)	0,972193432
IVT	Propionic Ac [g/L]	2.5.1.78:6,7-dimethyl-8-ribityllumazine synthase	0,971836432
IVV	Acetic Ac [g/L]	2.4.1.20:Cellobiose phosphorylase	0,971475661
IVT	Propionic Ac [g/L]	2.6.99.2:Pyridoxine 5'-phosphate synthase	0,971430469
IVT	Acetic Ac [g/L]	1.1.1.27:L-lactate dehydrogenase	0,971073588
IVV	Acetic Ac [g/L]	6.2.1.5:Succinate--CoA ligase (ADP-forming)	0,968512283
IVT	Acetic Ac [g/L]	1.1.1.103:L-threonine 3-dehydrogenase	0,967046905
IVT	Acetic Ac [g/L]	1.2.1.70:Glutamyl-tRNA reductase	0,966662887
IVV	Acetic Ac [g/L]	2.1.1.33:tRNA (guanine(46)-N(7))-methyltransferase	0,96616783
IVV	Propionic Ac [g/L]	4.1.1.65:Phosphatidylserine decarboxylase	0,965928496
IVT	Propionic Ac [g/L]	2.3.1.1:Amino-acid N-acetyltransferase	0,965907954
IVV	Acetic Ac [g/L]	4.2.1.8:Mannonate dehydratase	0,965715868
IVT	Acetic Ac [g/L]	3.5.1.5:Urease	0,96546426
IVV	Acetic Ac [g/L]	4.1.99.17:Phosphomethylpyrimidine synthase	0,964829464
IVV	Acetic Ac [g/L]	3.1.3.5:5'-nucleotidase	0,962550815
IVT	Propionic Ac [g/L]	2.1.1.74:(NAD(P)H-oxidizing)	0,962313523
IVT	Acetic Ac [g/L]	2.5.1.54:3-deoxy-7-phosphoheptulonate synthase	0,961346309
IVT	Acetic Ac [g/L]	1.2.7.8:Indolepyruvate ferredoxin oxidoreductase	0,960931094
IVT	Propionic Ac [g/L]	1.17.1.8:4-hydroxy-tetrahydrodipicolinate reductase	0,960578593
IVT	Acetic Ac [g/L]	2.7.1.191:Protein-N(pi)-phosphohistidine--D-mannose phosphotransferase	0,960156094
IVV	Acetic Ac [g/L]	2.5.1.78:6,7-dimethyl-8-ribityllumazine synthase	0,958843192
IVV	Propionic Ac [g/L]	2.5.1.55:3-deoxy-8-phosphooctulonate synthase	0,958504885
IVT	Acetic Ac [g/L]	1.1.1.100:3-oxoacyl-[acyl-carrier-protein] reductase	0,956403618
IVV	Acetic Ac [g/L]	2.7.7.7:DNA-directed DNA polymerase	0,955176075
IVT	Propionic Ac [g/L]	2.7.7.56:tRNA nucleotidyltransferase	0,953664862
IVT	Propionic Ac [g/L]	6.3.1.5:NAD(+) synthase	0,952703952
IVV	Acetic Ac [g/L]	1.1.1.133:dTDP-4-dehydrorhamnose reductase	0,952231368
IVT	Acetic Ac [g/L]	2.3.1.15:Glycerol-3-phosphate 1-O-acyltransferase	0,95199982
IVV	Propionic Ac [g/L]	2.4.2.1:Purine-nucleoside phosphorylase	0,951947936
IVT	Acetic Ac [g/L]	6.3.4.14:Biotin carboxylase	0,951660195
IVT	Propionic Ac [g/L]	3.6.1.23:dUTP diphosphatase	0,951197992
IVV	Acetic Ac [g/L]	6.2.1.30:Phenylacetate--CoA ligase	0,950296581
IVV	Acetic Ac [g/L]	3.5.1.2:Glutaminase	0,949232797
IVV	Butyric Ac [g/L]	7.2.1.1:NADH:ubiquinone reductase (Na(+)-transporting)	0,949106388
IVV	Butyric Ac [g/L]	2.7.1.90:Diphosphate--fructose-6-phosphate 1-phosphotransferase	0,948431057
IVV	Butyric Ac [g/L]	2.6.1.11:Acetylornithine transaminase	0,947587269
IVT	Propionic Ac [g/L]	2.4.1.21:Starch synthase	0,94731846
IVT	Propionic Ac [g/L]	2.7.2.15:Propionate kinase	0,946192566
IVV	Butyric Ac [g/L]	1.1.1.132:GDP-mannose 6-dehydrogenase	0,946015007
IVT	Acetic Ac [g/L]	2.1.1.14:5-methyltetrahydropteroyltriglutamate--homocysteine S-methyltransferase	0,945255622
IVV	Butyric Ac [g/L]	5.1.1.3:Glutamate racemase	0,944434212
IVV	Butyric Ac [g/L]	1.1.1.40:Malate dehydrogenase (oxaloacetate-decarboxylating) (NADP(+))	0,943841398
IVT	Propionic Ac [g/L]	6.3.5.7:Glutaminyl-tRNA synthase (glutamine-hydrolyzing)	0,943076012
IVT	Propionic Ac [g/L]	4.1.1.70:Transferred entry 7.2.4.5	0,94199432
IVT	Acetic Ac [g/L]	6.1.1.1:Tyrosine--tRNA ligase	0,941250796
IVT	Acetic Ac [g/L]	1.15.1.1:Superoxide dismutase	0,940189198
IVV	Butyric Ac [g/L]	2.7.1.5:Rhamnulokinase	0,939903011
IVV	Acetic Ac [g/L]	3.1.11.2:Exodeoxyribonuclease III	0,939788238
IVT	Acetic Ac [g/L]	1.2.1.72:Erythrose-4-phosphate dehydrogenase	0,939727193
IVT	Acetic Ac [g/L]	2.4.2.22:Xanthine phosphoribosyltransferase	0,939354073
IVV	Acetic Ac [g/L]	3.6.4.12:DNA helicase	0,939291351
IVV	Acetic Ac [g/L]	3.6.1.66:XTP/dITP diphosphatase	0,937939226
IVT	Acetic Ac [g/L]	2.1.1.176:16S rRNA (cytosine(967)-C(5))-methyltransferase	0,937635559
IVT	Propionic Ac [g/L]	4.2.1.47:GDP-mannose 4,6-dehydratase	0,936867967
IVV	Propionic Ac [g/L]	2.7.13.3:Histidine kinase	0,936746831
IVV	Butyric Ac [g/L]	5.3.1.16:isomerase	0,935334683
IVV	Acetic Ac [g/L]	1.17.7.1:(E)-4-hydroxy-3-methylbut-2-enyl-diphosphate synthase (ferredoxin)	0,93486894
IVV	Acetic Ac [g/L]	3.1.1.11:Pectinesterase	0,934050436
IVV	Butyric Ac [g/L]	2.3.1.39:[Acyl-carrier-protein] S-malonyltransferase	0,933523122
IVT	Acetic Ac [g/L]	5.1.3.20:ADP-glyceromanno-heptose 6-epimerase	0,933122335
IVV	Butyric Ac [g/L]	4.1.1.48:Indole-3-glycerol-phosphate synthase	0,932789109
IVT	Acetic Ac [g/L]	2.7.8.7:Holo-[acyl-carrier-protein] synthase	0,931497373
IVV	Acetic Ac [g/L]	4.3.2.1:Argininosuccinate lyase	0,930603472
IVT	Propionic Ac [g/L]	5.4.99.2:Methylmalonyl-CoA mutase	0,928701466
IVV	Butyric Ac [g/L]	4.2.3.4:3-dehydroquinate synthase	0,927073236
IVV	Acetic Ac [g/L]	4.6.1.12:2-C-methyl-D-erythritol 2,4-cyclodiphosphate synthase	0,926643528
IVT	Propionic Ac [g/L]	1.3.1.12:Prephenate dehydrogenase	0,926617241
IVT	Acetic Ac [g/L]	2.5.1.16:Spermidine synthase	0,925402416
IVV	Acetic Ac [g/L]	2.1.1.163:Demethylmenaquinone methyltransferase	0,924760826
IVT	Acetic Ac [g/L]	3.5.4.5:Cytidine deaminase	0,923952247
IVV	Acetic Ac [g/L]	5.1.3.3:Aldose 1-epimerase	0,92384363
IVV	Propionic Ac [g/L]	4.1.1.20:Diaminopimelate decarboxylase	0,923455624
IVT	Acetic Ac [g/L]	6.1.1.4:Leucine--tRNA ligase	0,923329462

IVT	Acetic Ac [g/L]	6.3.4.15:Biotin--[biotin carboxyl-carrier protein] ligase		0,920666118
IVT	Propionic Ac [g/L]	3.4.11.1:Leucyl aminopeptidase		0,920493323
IVT	Propionic Ac [g/L]	1.1.1.95:Phosphoglycerate dehydrogenase		0,920391972
IVV	Acetic Ac [g/L]	1.1.1.23:Histidinol dehydrogenase		0,919926834
IVT	Propionic Ac [g/L]	5.4.99.18:5-(carboxyamino)imidazole ribonucleotide mutase		0,918604828
IVT	Acetic Ac [g/L]	5.99.1.2:Transferred entry 5.6.2.1		0,918236071
IVV	Acetic Ac [g/L]	2.7.3.9:Phosphoenolpyruvate--protein phosphotransferase		0,917281656
IVV	Butyric Ac [g/L]	3.2.1.55:Non-reducing end alpha-L-arabinofuranosidase		0,915989591
IVV	Butyric Ac [g/L]	6.3.5.1:NAD(+) synthase (glutamine-hydrolyzing)		0,915594004
IVV	Propionic Ac [g/L]	3.1.1.73:Feruloyl esterase		0,915407152
IVV	Acetic Ac [g/L]	2.5.1.3:Thiamine phosphate synthase		0,914893116
IVV	Butyric Ac [g/L]	2.4.1.1:Glycogen phosphorylase		0,914861212
IVT	Propionic Ac [g/L]	6.4.1.1:Pyruvate carboxylase		0,914583823
IVV	Propionic Ac [g/L]	4.2.1.126:N-acetylmuramic acid 6-phosphate etherase		0,914268077
IVV	Butyric Ac [g/L]	3.2.1.37:Xylan 1,4-beta-xylosidase		0,914092402
IVT	Acetic Ac [g/L]	5.3.1.8:Mannose-6-phosphate isomerase		0,913587858
IVT	Propionic Ac [g/L]	2.6.1.90:dTDP-3-amino-3,6-dideoxy-alpha-D-galactopyranose transaminase		0,913079889
IVV	Acetic Ac [g/L]	2.1.1.14:5-methyltetrahydropteroyltriglutamate--homocysteine S-methyltransferase		0,912888195
IVV	Butyric Ac [g/L]	6.1.1.17:Glutamate--tRNA ligase		0,912235235
IVV	Acetic Ac [g/L]	3.4.14.5:Dipeptidyl-peptidase IV		0,908292792
IVV	Butyric Ac [g/L]	4.1.2.19:Rhamnulose-1-phosphate aldolase		0,905026005
IVV	Acetic Ac [g/L]	2.5.1.1:Dimethylallyltranstransferase		0,904084555
IVV	Acetic Ac [g/L]	2.5.1.10:(2E,6E)-farnesyl diphosphate synthase		0,904084555
IVT	Propionic Ac [g/L]	6.1.1.23:Aspartate--tRNA(Asn) ligase		0,904082474
IVT	Acetic Ac [g/L]	2.6.1.16:Glutamine--fructose-6-phosphate transaminase (isomerizing)		0,903657522
IVT	Propionic Ac [g/L]	2.3.1.29:Glycine C-acetyltransferase		0,901372155
IVT	Propionic Ac [g/L]	5.3.1.5:Xylose isomerase		0,900231899
IVT	Propionic Ac [g/L]	2.7.7.41:Phosphatidate cytidylyltransferase		0,899912726
IVV	Acetic Ac [g/L]	3.2.1.135:Neopullulanase		0,898401992
IVV	Propionic Ac [g/L]	2.4.2.22:Xanthine phosphoribosyltransferase		0,897329401
IVV	Propionic Ac [g/L]	4.1.1.48:Indole-3-glycerol-phosphate synthase		0,896658937
IVT	Acetic Ac [g/L]	2.1.1.177:23S rRNA (pseudouridine(1915)-N(3))-methyltransferase		0,89636334
IVV	Propionic Ac [g/L]	5.1.1.1:Alanine racemase		0,895573054
IVT	Acetic Ac [g/L]	2.7.4.9:dTMP kinase		0,894396993
IVT	Acetic Ac [g/L]	1.1.1.274:2,5-didehydrogluconate reductase (2-dehydro-D-gluconate-forming)		0,892632213
IVV	Acetic Ac [g/L]	4.1.1.20:Diaminopimelate decarboxylase		0,891650153
IVV	Butyric Ac [g/L]	3.1.3.2:Acid phosphatase		0,889337108
IVV	Acetic Ac [g/L]	2.7.1.40:Pyruvate kinase		0,884418337
IVV	Propionic Ac [g/L]	2.4.1.1:Glycogen phosphorylase		0,879916741
IVV	Acetic Ac [g/L]	3.1.3.3:Phosphoserine phosphatase		0,879823256
IVT	Acetic Ac [g/L]	2.1.1.207:tRNA (cytidine(34)-2'-O)-methyltransferase		0,878595493
IVT	Acetic Ac [g/L]	6.3.4.19:tRNA(Ile)-lysidine synthetase		0,878033611
IVV	Butyric Ac [g/L]	2.8.1.10:Thiazole synthase		0,877820547
IVV	Acetic Ac [g/L]	6.3.4.5:Argininosuccinate synthase		0,873759723
IVV	Butyric Ac [g/L]	3.2.1.74:Glucan 1,4-beta-glucosidase		0,873563513
IVT	Acetic Ac [g/L]	2.8.1.4:tRNA uracil 4-sulfurtransferase		0,872380994
IVV	Propionic Ac [g/L]	5.1.3.3:Aldose 1-epimerase		0,869952858
IVT	Acetic Ac [g/L]	5.4.3.8:Glutamate-1-semialdehyde 2,1-aminomutase		0,869310418
IVT	Propionic Ac [g/L]	4.1.2.50:6-carboxytetrahydropterin synthase		0,86901899
IVV	Butyric Ac [g/L]	2.3.1.8:Phosphate acetyltransferase		0,867211928
IVV	Propionic Ac [g/L]	2.2.1.9:synthase		0,866267613
IVV	Propionic Ac [g/L]	2.7.1.90:Diphosphate--fructose-6-phosphate 1-phosphotransferase		0,86615347
IVV	Propionic Ac [g/L]	5.2.1.8:Peptidylprolyl isomerase		0,864098212
IVV	Acetic Ac [g/L]	3.2.1.17:Lysozyme		0,863939332
IVV	Butyric Ac [g/L]	3.1.22.4:Crossover junction endodeoxyribonuclease		0,86384992
IVT	Propionic Ac [g/L]	2.7.7.33:Glucose-1-phosphate cytidylyltransferase		0,862508672
IVV	Butyric Ac [g/L]	2.3.1.180:Beta-ketoacyl-[acyl-carrier-protein] synthase III		0,860413415
IVT	Acetic Ac [g/L]	2.6.1.1:Aspartate transaminase		0,859116915
IVV	Acetic Ac [g/L]	2.7.7.60:2-C-methyl-D-erythritol 4-phosphate cytidylyltransferase		0,859011942
IVV	Acetic Ac [g/L]	4.2.1.126:N-acetylmuramic acid 6-phosphate etherase		0,858492601
IVV	Acetic Ac [g/L]	3.1.1.73:Feruloyl esterase		0,857973375
IVV	Acetic Ac [g/L]	3.2.1.74:Glucan 1,4-beta-glucosidase		0,856124683
IVT	Propionic Ac [g/L]	2.7.7.27:Glucose-1-phosphate adenylyltransferase		0,85568073
IVV	Propionic Ac [g/L]	3.6.4.12:DNA helicase		0,855163217
IVV	Butyric Ac [g/L]	2.7.1.23:NAD(+) kinase		0,854214742
IVV	Butyric Ac [g/L]	1.3.1.98:UDP-N-acetylmuramate dehydrogenase		0,853975404
IVT	Propionic Ac [g/L]	4.2.1.46:dTDP-glucose 4,6-dehydratase		0,852452303
IVV	Butyric Ac [g/L]	3.5.99.6:Glucosamine-6-phosphate deaminase		0,852242348
IVT	Acetic Ac [g/L]	2.7.2.2:Carbamate kinase		0,850069728
IVT	Acetic Ac [g/L]	2.4.2.4:Thymidine phosphorylase		0,849702939
IVV	Butyric Ac [g/L]	4.1.1.65:Phosphatidylserine decarboxylase		0,849098888
IVT	Propionic Ac [g/L]	3.5.4.9:Methenyltetrahydrofolate cyclohydrolase		0,848855496
IVT	Propionic Ac [g/L]	3.4.25.2:HslU--HslV peptidase		0,847820382
IVV	Acetic Ac [g/L]	2.1.1.177:23S rRNA (pseudouridine(1915)-N(3))-methyltransferase		0,847371166
IVV	Acetic Ac [g/L]	2.8.1.7:Cysteine desulfurase		0,845220243
IVT	Propionic Ac [g/L]	3.6.3.34:Transferred entry 7.2.2.16		0,845196249
IVT	Acetic Ac [g/L]	2.6.1.37:2-aminoethylphosphonate--pyruvate transaminase		0,842385491
IVV	Propionic Ac [g/L]	2.1.1.163:Demethylmenaquinone methyltransferase		0,841274529
IVV	Acetic Ac [g/L]	3.1.21.3:Type I site-specific deoxyribonuclease		0,841087818
IVV	Propionic Ac [g/L]	3.5.1.2:Glutaminase		0,839957929
IVV	Acetic Ac [g/L]	3.4.21.102:C-terminal processing peptidase		0,839545162
IVT	Propionic Ac [g/L]	2.5.1.49:O-acetylhomoserine aminocarboxypropyltransferase		0,837920915
IVV	Butyric Ac [g/L]	2.4.1.227:acetylglucosaminyltransferase		0,837612495

IVV	Acetic Ac [g/L]	2.4.2.1:Purine-nucleoside phosphorylase		0,837368714
IVT	Propionic Ac [g/L]	2.7.7.8:Polyribonucleotide nucleotidyltransferase		0,837151094
IVV	Propionic Ac [g/L]	6.3.4.5:Argininosuccinate synthase		0,83713247
IVT	Propionic Ac [g/L]	4.2.1.9:Dihydroxy-acid dehydratase		0,833748066
IVV	Butyric Ac [g/L]	2.1.1.207:tRNA (cytidine(34)-2'-O)-methyltransferase		0,8335039
IVT	Propionic Ac [g/L]	4.1.1.49:Phosphoenolpyruvate carboxykinase (ATP)		0,832387703
IVV	Butyric Ac [g/L]	2.3.1.31:Homoserine O-acetyltransferase		0,832360087
IVV	Butyric Ac [g/L]	3.1.26.11:Ribonuclease Z		0,832289869
IVT	Acetic Ac [g/L]	2.4.2.3:Uridine phosphorylase		0,832175024
IVT	Acetic Ac [g/L]	5.5.1.4:Inositol-3-phosphate synthase		0,831706275
IVT	Acetic Ac [g/L]	5.3.1.24:Phosphoribosylanthranilate isomerase		0,831384853
IVT	Propionic Ac [g/L]	1.6.99.5:Transferred entry 1.6.5.11		0,831128795
IVV	Propionic Ac [g/L]	3.4.11.4:Tripeptide aminopeptidase		0,828887377
IVV	Acetic Ac [g/L]	2.7.13.3:Histidine kinase		0,82863614
IVV	Propionic Ac [g/L]	6.1.1.21:Histidine--tRNA ligase		0,828321274
IVV	Acetic Ac [g/L]	2.3.1.54:Formate C-acetyltransferase		0,828116504
IVT	Propionic Ac [g/L]	2.5.1.61:Hydroxymethylbilane synthase		0,827596433
IVV	Acetic Ac [g/L]	4.2.1.11:Phosphopyruvate hydratase		0,825401643
IVV	Acetic Ac [g/L]	5.4.99.2:Methylmalonyl-CoA mutase		0,825360985
IVT	Propionic Ac [g/L]	6.1.1.14:Glycine--tRNA ligase		0,824988765
IVV	Butyric Ac [g/L]	2.4.2.10:Orotate phosphoribosyltransferase		0,824175981
IVV	Acetic Ac [g/L]	5.1.1.1:Alanine racemase		0,823332529
IVV	Propionic Ac [g/L]	2.3.1.31:Homoserine O-acetyltransferase		0,823241903
IVV	Butyric Ac [g/L]	6.3.4.14:Biotin carboxylase		0,823199098
IVT	Propionic Ac [g/L]	2.3.1.18:Galactoside O-acetyltransferase		0,822571619
IVV	Acetic Ac [g/L]	2.8.1.10:Thiazole synthase		0,82208056
IVV	Propionic Ac [g/L]	4.1.99.17:Phosphomethylpyrimidine synthase		0,820321115
IVT	Propionic Ac [g/L]	4.2.1.2:Fumarate hydratase		0,819987068
IVV	Butyric Ac [g/L]	3.1.1.73:Feruloyl esterase		0,819880128
IVT	Propionic Ac [g/L]	5.3.1.9:Glucose-6-phosphate isomerase		0,817811506
IVT	Acetic Ac [g/L]	5.1.3.13:dTDP-4-dehydrorhamnose 3,5-epimerase		0,815652445
IVT	Propionic Ac [g/L]	2.1.3.3:Ornithine carbamoyltransferase		0,815366864
IVT	Propionic Ac [g/L]	1.1.1.22:UDP-glucose 6-dehydrogenase		0,815243242
IVV	Propionic Ac [g/L]	2.4.99.17:S-adenosylmethionine:tRNA ribosyltransferase-isomerase		0,813950027
IVV	Butyric Ac [g/L]	5.5.1.4:Inositol-3-phosphate synthase		0,813757454
IVV	Propionic Ac [g/L]	2.4.1.20:Cellobiose phosphorylase		0,812865733
IVV	Acetic Ac [g/L]	6.3.2.10:UDP-N-acetylmuramoyl-tripeptide--D-alanyl-D-alanine ligase		0,812647276
IVT	Propionic Ac [g/L]	1.1.1.271:GDP-L-fucose synthase		0,812302226
IVT	Acetic Ac [g/L]	3.5.99.6:Glucosamine-6-phosphate deaminase		0,811598496
IVV	Acetic Ac [g/L]	4.2.3.4:3-dehydroquinate synthase		0,811432713
IVV	Propionic Ac [g/L]	2.1.1.37:DNA (cytosine-5-)-methyltransferase		0,811113025
IVV	Propionic Ac [g/L]	3.1.1.11:Pectinesterase		0,810459865
IVV	Propionic Ac [g/L]	2.7.1.23:NAD(+) kinase		0,808580461
IVV	Butyric Ac [g/L]	6.1.1.2:Tryptophan--tRNA ligase		0,807853343
IVT	Propionic Ac [g/L]	2.7.4.22:UMP kinase		0,804570561
IVV	Butyric Ac [g/L]	3.2.1.23:Beta-galactosidase		0,803900732
IVV	Acetic Ac [g/L]	1.1.1.69:Gluconate 5-dehydrogenase		0,803268681
IVV	Butyric Ac [g/L]	2.4.99.17:S-adenosylmethionine:tRNA ribosyltransferase-isomerase		0,80295765
IVT	Acetic Ac [g/L]	1.3.1.98:UDP-N-acetylmuramate dehydrogenase		0,802829137
IVT	Propionic Ac [g/L]	2.4.2.4:Thymidine phosphorylase		0,802156493
IVT	Propionic Ac [g/L]	2.7.2.2:Carbamate kinase		0,801722688
IVV	Acetic Ac [g/L]	4.2.99.18:DNA-(apurinic or apyrimidinic site) lyase		0,801011089
IVT	Propionic Ac [g/L]	6.3.5.2:GMP synthase (glutamine-hydrolyzing)		0,80045482
IVV	Acetic Ac [g/L]	6.1.1.21:Histidine--tRNA ligase		0,799919351
IVV	Butyric Ac [g/L]	2.6.1.83:LL-diaminopimelate aminotransferase		0,797949351
IVV	Propionic Ac [g/L]	3.1.26.4:Ribonuclease H		0,797386561
IVV	Acetic Ac [g/L]	6.3.4.13:Phosphoribosylamine--glycine ligase		0,796585775
IVV	Propionic Ac [g/L]	4.2.3.4:3-dehydroquinate synthase		0,796096691
IVV	Propionic Ac [g/L]	6.3.5.1:NAD(+) synthase (glutamine-hydrolyzing)		0,795563651
IVV	Butyric Ac [g/L]	5.3.1.24:Phosphoribosylanthranilate isomerase		0,794912582
IVV	Acetic Ac [g/L]	4.2.1.75:Uroporphyrinogen-III synthase		0,792046523
IVV	Butyric Ac [g/L]	3.1.1.11:Pectinesterase		0,791988602
IVV	Butyric Ac [g/L]	2.1.1.163:Demethylmenaquinone methyltransferase		0,791545021
IVV	Butyric Ac [g/L]	2.3.1.30:Serine O-acetyltransferase		0,791489138
IVV	Butyric Ac [g/L]	2.7.1.92:5-dehydro-2-deoxygluconokinase		0,790312417
IVT	Acetic Ac [g/L]	2.6.1.76:Diaminobutyrate--2-oxoglutarate transaminase		0,788900257
IVT	Propionic Ac [g/L]	1.5.1.7:Saccharopine dehydrogenase (NAD(+), L-lysine-forming)		0,788597958
IVV	Butyric Ac [g/L]	3.2.1.135:Neopullulanase		0,788509905
IVV	Acetic Ac [g/L]	1.1.1.218:Morphine 6-dehydrogenase		0,787807724
IVV	Acetic Ac [g/L]	4.3.3.6:Pyridoxal 5'-phosphate synthase (glutamine hydrolyzing)		0,787685443
IVT	Acetic Ac [g/L]	2.4.2.7:Adenine phosphoribosyltransferase		0,787669275
IVV	Acetic Ac [g/L]	6.3.1.2:Glutamine synthetase		0,787666871
IVT	Acetic Ac [g/L]	2.7.13.3:Histidine kinase		0,787256615
IVV	Acetic Ac [g/L]	5.3.1.16:isomerase		0,787106566
IVV	Acetic Ac [g/L]	1.3.1.98:UDP-N-acetylmuramate dehydrogenase		0,784519678
IVV	Acetic Ac [g/L]	1.97.1.4:[Formate-C-acetyltransferase]-activating enzyme		0,783727267
IVT	Acetic Ac [g/L]	3.2.1.17:Lysozyme		0,783270959
IVV	Acetic Ac [g/L]	6.3.5.4:Asparagine synthase (glutamine-hydrolyzing)		0,782777765
IVT	Propionic Ac [g/L]	5.4.2.10:Phosphoglucosamine mutase		0,782719064
IVV	Acetic Ac [g/L]	2.4.1.1:Glycogen phosphorylase		0,781695681
IVV	Butyric Ac [g/L]	3.4.22.40:Bleomycin hydrolase		0,781040708
IVV	Butyric Ac [g/L]	5.2.1.8:Peptidylprolyl isomerase		0,779977202
IVV	Butyric Ac [g/L]	2.8.1.13:tRNA-uridine 2-sulfurtransferase		0,779924055

IVV	Propionic Ac [g/L]	1.1.1.40:Malate dehydrogenase (oxaloacetate-decarboxylating) (NADP(+))	0,779504412
IVT	Acetic Ac [g/L]	1.8.5.3:Respiratory dimethylsulfoxide reductase	0,779353602
IVV	Acetic Ac [g/L]	2.7.2.11:Glutamate 5-kinase	0,776301799
IVV	Acetic Ac [g/L]	3.4.23.36:Signal peptidase II	0,776285516
IVT	Propionic Ac [g/L]	2.5.1.47:Cysteine synthase	0,776038173
IVV	Propionic Ac [g/L]	4.3.2.10:Imidazole glycerol-phosphate synthase	0,775542339
IVV	Acetic Ac [g/L]	1.1.1.95:Phosphoglycerate dehydrogenase	0,775068565
IVV	Acetic Ac [g/L]	2.6.1.83:LL-diaminopimelate aminotransferase	0,774942022
IVV	Acetic Ac [g/L]	2.4.2.29:tRNA-guanine(34) transglycosylase	0,774612548
IVV	Propionic Ac [g/L]	1.17.7.3:(E)-4-hydroxy-3-methylbut-2-enyl-diphosphate synthase (flavodoxin)	0,772955067
IVT	Propionic Ac [g/L]	2.1.1.198:16S rRNA (cytidine(1402)-2'-O)-methyltransferase	0,772586878
IVV	Butyric Ac [g/L]	3.5.4.16:GTP cyclohydrolase I	0,772327714
IVV	Acetic Ac [g/L]	2.7.2.7:Butyrate kinase	0,771829899
IVT	Acetic Ac [g/L]	5.4.2.10:Phosphoglucosamine mutase	0,771608835
IVT	Acetic Ac [g/L]	3.5.2.3:Dihydroorotase	0,770855736
IVV	Butyric Ac [g/L]	2.4.2.1:Purine-nucleoside phosphorylase	0,769264476
IVV	Butyric Ac [g/L]	4.1.1.3:Transferred entry 4.1.1.112	0,766113319
IVV	Acetic Ac [g/L]	4.2.1.9:Dihydroxy-acid dehydratase	0,764514032
IVV	Acetic Ac [g/L]	1.4.1.13:Glutamate synthase (NADPH)	0,763593602
IVV	Butyric Ac [g/L]	1.1.1.86:Ketol-acid reductoisomerase (NADP(+))	0,762968063
IVV	Propionic Ac [g/L]	3.6.1.66:XTP/dITP diphosphatase	0,762698767
IVT	Acetic Ac [g/L]	3.6.3.15:Transferred entry 7.2.2.1	0,762610858
IVV	Acetic Ac [g/L]	2.2.1.7:1-deoxy-D-xylulose-5-phosphate synthase	0,762335429
IVT	Acetic Ac [g/L]	3.6.3.34:Transferred entry 7.2.2.16	0,7604766
IVV	Acetic Ac [g/L]	6.1.1.19:Arginine--tRNA ligase	0,759876231
IVT	Acetic Ac [g/L]	6.3.3.2:5-formyltetrahydrofolate cyclo-ligase	0,759531669
IVV	Butyric Ac [g/L]	3.4.21.102:C-terminal processing peptidase	0,758954259
IVT	Acetic Ac [g/L]	1.1.1.79:Glyoxylate reductase (NADP(+))	0,757399578
IVV	Propionic Ac [g/L]	2.5.1.75:tRNA dimethylallyltransferase	0,754963383
IVT	Acetic Ac [g/L]	2.3.1.12:Dihydrolipoyllysine-residue acetyltransferase	0,754468639
IVV	Propionic Ac [g/L]	3.4.14.5:Dipeptidyl-peptidase IV	0,75440655
IVV	Acetic Ac [g/L]	2.3.1.1:Amino-acid N-acetyltransferase	0,754224166
IVV	Acetic Ac [g/L]	2.1.1.34:tRNA (guanosine(18)-2'-O)-methyltransferase	0,754224166
IVV	Acetic Ac [g/L]	3.6.1.7:Acylphosphatase	0,754224166
IVV	Acetic Ac [g/L]	2.6.1.39:2-aminoadipate transaminase	0,754224166
IVV	Acetic Ac [g/L]	3.1.1.72:Acetylxylan esterase	0,754224166
IVV	Acetic Ac [g/L]	3.4.15.5:Peptidyl-dipeptidase Dcp	0,754224166
IVV	Acetic Ac [g/L]	4.6.1.1:Adenylate cyclase	0,754224166
IVV	Acetic Ac [g/L]	5.1.3.20:ADP-glyceromanno-heptose 6-epimerase	0,754224166
IVV	Acetic Ac [g/L]	6.3.4.15:Biotin--[biotin carboxyl-carrier protein] ligase	0,754224166
IVV	Acetic Ac [g/L]	1.3.5.4:Fumarate reductase (quinol)	0,754224166
IVV	Acetic Ac [g/L]	2.6.1.62:Adenosylmethionine--8-amino-7-oxononanoate transaminase	0,754224166
IVV	Acetic Ac [g/L]	3.2.1.14:Chitinase	0,754224166
IVV	Acetic Ac [g/L]	3.2.1.26:Beta-fructofuranosidase	0,754224166
IVV	Acetic Ac [g/L]	2.7.4.6:Nucleoside-diphosphate kinase	0,753679807
IVV	Butyric Ac [g/L]	3.4.21.88:Repressor LexA	0,753511306
IVV	Propionic Ac [g/L]	2.1.1.195:Cobalt-precorrin-5B (C(1))-methyltransferase	0,752936359
IVV	Propionic Ac [g/L]	3.4.21.53:Endopeptidase La	0,751152308
IVT	Acetic Ac [g/L]	3.6.4.12:DNA helicase	0,750699203
IVV	Propionic Ac [g/L]	2.1.1.33:tRNA (guanine(46)-N(7))-methyltransferase	0,75026328
IVV	Propionic Ac [g/L]	1.17.4.1:Ribonucleoside-diphosphate reductase	0,750072693
IVV	Butyric Ac [g/L]	2.5.1.15:Dihydropteroate synthase	0,749616352
IVT	Propionic Ac [g/L]	6.3.4.21:Nicotinate phosphoribosyltransferase	0,74945063
IVV	Butyric Ac [g/L]	2.5.1.1:Dimethylallyltranstransferase	0,748887269
IVV	Butyric Ac [g/L]	2.5.1.10:(2E,6E)-farnesyl diphosphate synthase	0,748887269
IVV	Butyric Ac [g/L]	2.2.1.7:1-deoxy-D-xylulose-5-phosphate synthase	0,748789539
IVT	Propionic Ac [g/L]	3.5.1.10:Formyltetrahydrofolate deformylase	0,747657933
IVV	Acetic Ac [g/L]	1.1.1.86:Ketol-acid reductoisomerase (NADP(+))	0,745953661
IVT	Acetic Ac [g/L]	2.6.1.82:Putrescine--2-oxoglutarate transaminase	0,743698412
IVT	Acetic Ac [g/L]	2.3.3.1:Citrate (Si)-synthase	0,741301247
IVT	Acetic Ac [g/L]	6.3.2.4:D-alanine--D-alanine ligase	0,741229595
IVV	Propionic Ac [g/L]	5.4.99.25:tRNA pseudouridine(55) synthase	0,740236803
IVV	Propionic Ac [g/L]	2.7.7.18:Nicotinate-nucleotide adenylyltransferase	0,73973343
IVV	Propionic Ac [g/L]	2.1.1.148:Thymidylate synthase (FAD)	0,739691317
IVV	Acetic Ac [g/L]	6.3.2.8:UDP-N-acetylmuramate--L-alanine ligase	0,739173492
IVV	Butyric Ac [g/L]	2.2.1.9:synthase	0,739165534
IVV	Propionic Ac [g/L]	2.7.4.3:Adenylate kinase	0,738489794
IVV	Butyric Ac [g/L]	3.6.4.12:DNA helicase	0,738399502
IVV	Acetic Ac [g/L]	2.7.1.5:Rhamnulokinase	0,737520623
IVV	Acetic Ac [g/L]	7.2.1.1:NADH:ubiquinone reductase (Na(+)-transporting)	0,737290822
IVV	Acetic Ac [g/L]	4.2.3.3:Methylglyoxal synthase	0,736910622
IVV	Butyric Ac [g/L]	5.4.99.25:tRNA pseudouridine(55) synthase	0,735051054
IVV	Propionic Ac [g/L]	3.1.22.4:Crossover junction endodeoxyribonuclease	0,733680313
IVV	Butyric Ac [g/L]	4.2.1.11:Phosphopyruvate hydratase	0,732793603
IVT	Acetic Ac [g/L]	2.7.1.19:Phosphoribulokinase	0,731774605
IVT	Acetic Ac [g/L]	3.1.1.96:D-aminoacyl-tRNA deacylase	0,730385755
IVT	Propionic Ac [g/L]	6.1.1.15:Proline--tRNA ligase	0,729335505
IVV	Butyric Ac [g/L]	3.2.2.n1:Cytokinin riboside 5'-monophosphate phosphoribohydrolase	0,729265097
IVT	Propionic Ac [g/L]	2.4.2.14:Amidophosphoribosyltransferase	0,728540666
IVV	Propionic Ac [g/L]	2.3.3.16:Citrate synthase (unknown stereospecificity)	0,728524596
IVV	Propionic Ac [g/L]	4.3.99.3:7-carboxy-7-deazaguanine synthase	0,728524596
IVV	Propionic Ac [g/L]	1.5.98.2:5,10-methylenetetrahydromethanopterin reductase	0,728524596
IVV	Propionic Ac [g/L]	3.2.1.41:Pullulanase	0,728524596

IVV	Propionic Ac [g/L]	4.2.1.70:Pseudouridylate synthase	0,728524596
IVV	Propionic Ac [g/L]	5.1.3.23:UDP-2,3-diacetamido-2,3-dideoxyglucuronic acid 2-epimerase	0,728524596
IVV	Propionic Ac [g/L]	1.18.6.1:Nitrogenase	0,728524596
IVV	Propionic Ac [g/L]	1.3.1.6:Fumarate reductase (NADH)	0,728524596
IVV	Propionic Ac [g/L]	1.3.1.91:tRNA-dihydrouridine(20) synthase (NAD(P)(+))	0,728524596
IVV	Propionic Ac [g/L]	1.3.5.1:Succinate dehydrogenase (quinone)	0,728524596
IVV	Propionic Ac [g/L]	2.3.1.n3:Transferred entry 2.3.1.275	0,728524596
IVV	Propionic Ac [g/L]	2.3.2.6:Lysine/arginine leucyltransferase	0,728524596
IVV	Propionic Ac [g/L]	2.4.2.3:Uridine phosphorylase	0,728524596
IVV	Propionic Ac [g/L]	2.7.9.2:Pyruvate, water dikinase	0,728524596
IVV	Propionic Ac [g/L]	2.8.1.6:Biotin synthase	0,728524596
IVV	Propionic Ac [g/L]	2.8.4.1:Coenzyme-B sulfoethylthiotransferase	0,728524596
IVV	Propionic Ac [g/L]	4.2.1.24:Porphobilinogen synthase	0,728524596
IVV	Propionic Ac [g/L]	5.4.3.8:Glutamate-1-semialdehyde 2,1-aminomutase	0,728524596
IVV	Propionic Ac [g/L]	6.5.1.1:DNA ligase (ATP)	0,728524596
IVV	Propionic Ac [g/L]	1.1.5.3:Glycerol-3-phosphate dehydrogenase	0,728524596
IVV	Propionic Ac [g/L]	1.16.3.1:Ferroxidase	0,728524596
IVV	Propionic Ac [g/L]	1.4.1.2:Glutamate dehydrogenase	0,728524596
IVV	Propionic Ac [g/L]	1.9.6.1:Nitrate reductase (cytochrome)	0,728524596
IVV	Propionic Ac [g/L]	2.1.1.133:Precorrin-4 C(11)-methyltransferase	0,728524596
IVV	Propionic Ac [g/L]	2.1.1.166:23S rRNA (uridine(2552)-2'-O)-methyltransferase	0,728524596
IVV	Propionic Ac [g/L]	2.1.1.206:tRNA (cytidine(56)-2'-O)-methyltransferase	0,728524596
IVV	Propionic Ac [g/L]	2.3.1.222:Phosphate propanoyltransferase	0,728524596
IVV	Propionic Ac [g/L]	2.3.3.1:Citrate (Si)-synthase	0,728524596
IVV	Propionic Ac [g/L]	2.4.2.15:Guanosine phosphorylase	0,728524596
IVV	Propionic Ac [g/L]	2.4.2.2:Pyrimidine-nucleoside phosphorylase	0,728524596
IVV	Propionic Ac [g/L]	2.4.2.4:Thymidine phosphorylase	0,728524596
IVV	Propionic Ac [g/L]	2.6.1.37:2-aminoethylphosphonate--pyruvate transaminase	0,728524596
IVV	Propionic Ac [g/L]	2.7.1.17:Xylulokinase	0,728524596
IVV	Propionic Ac [g/L]	2.7.11.32:[Pyruvate, phosphate dikinase] kinase	0,728524596
IVV	Propionic Ac [g/L]	2.7.4.27:([Pyruvate, phosphate dikinase] phosphate) phosphotransferase	0,728524596
IVV	Propionic Ac [g/L]	3.2.1.131:Xylan alpha-1,2-glucuronosidase	0,728524596
IVV	Propionic Ac [g/L]	3.5.4.5:Cytidine deaminase	0,728524596
IVV	Propionic Ac [g/L]	3.6.1.8:ATP diphosphatase	0,728524596
IVV	Propionic Ac [g/L]	4.2.1.32:L(+)-tartrate dehydratase	0,728524596
IVV	Propionic Ac [g/L]	4.3.1.19:Threonine ammonia-lyase	0,728524596
IVV	Propionic Ac [g/L]	6.2.1.14:6-carboxyhexanoate--CoA ligase	0,728524596
IVV	Propionic Ac [g/L]	6.4.1.1:Pyruvate carboxylase	0,728524596
IVV	Propionic Ac [g/L]	3.2.1.89:Arabinogalactan endo-beta-1,4-galactanase	0,728524596
IVV	Propionic Ac [g/L]	6.1.1.23:Aspartate--tRNA(Asn) ligase	0,728524596
IVV	Propionic Ac [g/L]	2.4.2.48:tRNA-guanine(15) transglycosylase	0,728524596
IVV	Propionic Ac [g/L]	2.5.1.61:Hydroxymethylbilane synthase	0,728524596
IVV	Propionic Ac [g/L]	3.4.14.12:Xaa-Xaa-Pro tripeptidyl-peptidase	0,728524596
IVV	Propionic Ac [g/L]	3.4.25.1:Proteasome endopeptidase complex	0,728524596
IVV	Propionic Ac [g/L]	6.3.4.19:tRNA(Ile)-lysidine synthetase	0,728524596
IVV	Butyric Ac [g/L]	3.4.23.36:Signal peptidase II	0,726652891
IVV	Acetic Ac [g/L]	2.5.1.19:3-phosphoshikimate 1-carboxyvinyltransferase	0,723000675
IVT	Acetic Ac [g/L]	2.3.1.157:Glucosamine-1-phosphate N-acetyltransferase	0,722739456
IVT	Acetic Ac [g/L]	1.1.1.42:Isocitrate dehydrogenase (NADP(+))	0,722143304
IVT	Acetic Ac [g/L]	2.4.1.1:Glycogen phosphorylase	0,721769492
IVV	Butyric Ac [g/L]	4.1.2.13:Fructose-bisphosphate aldolase	0,721596478
IVT	Acetic Ac [g/L]	1.3.1.12:Prephenate dehydrogenase	0,720220971
IVT	Acetic Ac [g/L]	2.7.1.148:4-(cytidine 5'-diphospho)-2-C-methyl-D-erythritol kinase	0,71915022
IVV	Propionic Ac [g/L]	2.4.2.21:Nicotinate-nucleotide--dimethylbenzimidazole phosphoribosyltransferase	0,717963175
IVV	Acetic Ac [g/L]	1.7.2.2:Nitrite reductase (cytochrome ammonia-forming)	0,717438804
IVV	Butyric Ac [g/L]	1.1.1.95:Phosphoglycerate dehydrogenase	0,71693121
IVT	Acetic Ac [g/L]	2.7.8.26:Adenosylcobinamide-GDP ribazoletransferase	0,7166554
IVT	Acetic Ac [g/L]	2.8.1.8:Lipoyl synthase	0,714885249
IVV	Butyric Ac [g/L]	3.6.1.27:Undecaprenyl-diphosphate phosphatase	0,714628604
IVT	Acetic Ac [g/L]	6.3.2.17:Tetrahydrofolate synthase	0,714243735
IVT	Acetic Ac [g/L]	2.7.7.75:Molybdopterin adenylyltransferase	0,713945904
IVV	Acetic Ac [g/L]	2.1.1.193:16S rRNA (uracil(1498)-N(3))-methyltransferase	0,710392484
IVT	Acetic Ac [g/L]	6.3.5.2:GMP synthase (glutamine-hydrolyzing)	0,709200888
IVV	Acetic Ac [g/L]	2.7.7.38:3-deoxy-manno-octulosonate cytidylyltransferase	0,708639605
IVV	Propionic Ac [g/L]	1.5.1.3:Dihydrofolate reductase	0,708593553
IVV	Butyric Ac [g/L]	2.4.2.21:Nicotinate-nucleotide--dimethylbenzimidazole phosphoribosyltransferase	0,708251177
IVT	Propionic Ac [g/L]	2.5.1.7:UDP-N-acetylglucosamine 1-carboxyvinyltransferase	0,708075287
IVV	Acetic Ac [g/L]	2.1.1.207:tRNA (cytidine(34)-2'-O)-methyltransferase	0,705331907
IVV	Acetic Ac [g/L]	4.2.3.1:Threonine synthase	0,705304961
IVT	Acetic Ac [g/L]	3.4.11.1:Leucyl aminopeptidase	0,704007168
IVT	Propionic Ac [g/L]	3.5.3.11:Agmatinase	0,700780661
IVT	Propionic Ac [g/L]	2.3.1.191:UDP-3-O-(3-hydroxymyristoyl)glucosamine N-acyltransferase	0,700780661
IVT	Propionic Ac [g/L]	3.6.1.1:Inorganic diphosphatase	0,700780661
IVT	Propionic Ac [g/L]	5.4.2.8:Phosphomannomutase	0,700780661
IVT	Propionic Ac [g/L]	2.7.1.39:Homoserine kinase	0,700780661
IVT	Propionic Ac [g/L]	4.2.1.49:Urocanate hydratase	0,700780661
IVT	Propionic Ac [g/L]	3.5.2.7:Imidazolonepropionase	0,700780661
IVT	Propionic Ac [g/L]	1.16.1.3:Aquacobalamin reductase	0,700780661
IVT	Propionic Ac [g/L]	1.3.3.3:Coproporphyrinogen oxidase	0,700780661
IVT	Propionic Ac [g/L]	2.7.1.45:2-dehydro-3-deoxygluconokinase	0,700780661
IVT	Propionic Ac [g/L]	3.5.4.13:dCTP deaminase	0,700780661
IVT	Propionic Ac [g/L]	5.4.99.23:23S rRNA pseudouridine(1911/1915/1917) synthase	0,700780661
IVT	Propionic Ac [g/L]	2.7.1.6:Galactokinase	0,700780661

IVT	Propionic Ac [g/L]	6.2.1.14:6-carboxyhexanoate--CoA ligase		0,700780661
IVT	Propionic Ac [g/L]	1.13.11.54:Acireductone dioxygenase (Fe(2+)-requiring)		0,700780661
IVT	Propionic Ac [g/L]	4.1.1.68:5-oxopent-3-ene-1,2,5-tricarboxylate decarboxylase		0,700780661
IVT	Propionic Ac [g/L]	3.6.3.25:Transferred entry 7.3.2.3		0,700780661
IVT	Acetic Ac [g/L]	3.2.1.51:Alpha-L-fucosidase		0,699918912
IVV	Butyric Ac [g/L]	6.3.1.2:Glutamine synthetase		0,699064366
IVT	Acetic Ac [g/L]	2.4.2.9:Uracil phosphoribosyltransferase		0,698357572
IVT	Propionic Ac [g/L]	2.7.7.72:CCA tRNA nucleotidyltransferase		0,697540175
IVV	Propionic Ac [g/L]	2.1.1.199:16S rRNA (cytosine(1402)-N(4))-methyltransferase		0,697298037
IVT	Propionic Ac [g/L]	2.6.1.37:2-aminoethylphosphonate--pyruvate transaminase		0,69542163
IVT	Propionic Ac [g/L]	1.15.1.1:Superoxide dismutase		0,695305023
IVT	Acetic Ac [g/L]	1.5.1.42:FMN reductase (NADH)		0,692541437
IVV	Butyric Ac [g/L]	4.2.1.1:Carbonic anhydrase		0,691617962
IVT	Acetic Ac [g/L]	3.6.1.66:XTP/dITP diphosphatase		0,691156538
IVV	Acetic Ac [g/L]	1.15.1.1:Superoxide dismutase		0,690919065
IVV	Acetic Ac [g/L]	1.11.1.1:NADH peroxidase		0,689246951
IVV	Propionic Ac [g/L]	3.2.1.23:Beta-galactosidase		0,689161819
IVV	Butyric Ac [g/L]	4.1.99.17:Phosphomethylpyrimidine synthase		0,688618033
IVV	Propionic Ac [g/L]	2.3.1.8:Phosphate acetyltransferase		0,686102739
IVT	Acetic Ac [g/L]	2.5.1.9:Riboflavin synthase		0,686095134
IVV	Propionic Ac [g/L]	2.7.7.60:2-C-methyl-D-erythritol 4-phosphate cytidylyltransferase		0,685910811
IVT	Propionic Ac [g/L]	2.7.7.6:DNA-directed RNA polymerase		0,685572516
IVV	Propionic Ac [g/L]	5.3.1.16:isomerase		0,684550433
IVV	Butyric Ac [g/L]	4.3.2.1:Argininosuccinate lyase		0,68427981
IVT	Acetic Ac [g/L]	2.8.4.3:tRNA-2-methylthio-N(6)-dimethylallyladenosine synthase		0,683506184
IVT	Propionic Ac [g/L]	6.3.4.3:Formate--tetrahydrofolate ligase		0,682425735
IVT	Acetic Ac [g/L]	2.7.2.15:Propionate kinase		0,681939064
IVT	Propionic Ac [g/L]	1.17.7.1:(E)-4-hydroxy-3-methylbut-2-enyl-diphosphate synthase (ferredoxin)		0,681699061
IVV	Acetic Ac [g/L]	4.1.1.65:Phosphatidylserine decarboxylase		0,680531081
IVT	Acetic Ac [g/L]	4.1.1.3:Transferred entry 4.1.1.112		0,677906112
IVV	Acetic Ac [g/L]	2.7.2.4:Aspartate kinase		0,677889901
IVT	Propionic Ac [g/L]	6.1.1.9:Valine--tRNA ligase		0,676555257
IVT	Propionic Ac [g/L]	6.1.1.16:Cysteine--tRNA ligase		0,674734299
IVV	Butyric Ac [g/L]	2.7.7.7:DNA-directed DNA polymerase		0,674141833
IVV	Butyric Ac [g/L]	4.2.3.1:Threonine synthase		0,673091059
IVT	Acetic Ac [g/L]	6.3.5.4:Asparagine synthase (glutamine-hydrolyzing)		0,672418706
IVT	Acetic Ac [g/L]	4.1.1.48:Indole-3-glycerol-phosphate synthase		0,672345537
IVV	Butyric Ac [g/L]	2.7.1.21:Thymidine kinase		0,672013546
IVT	Acetic Ac [g/L]	2.1.1.61:tRNA (5-methylaminomethyl-2-thiouridylate)-methyltransferase		0,670734012
IVV	Propionic Ac [g/L]	6.1.1.5:Isoleucine--tRNA ligase		0,670472904
IVT	Acetic Ac [g/L]	4.3.2.10:Imidazole glycerol-phosphate synthase		0,669944365
IVV	Butyric Ac [g/L]	2.4.2.7:Adenine phosphoribosyltransferase		0,669773004
IVT	Propionic Ac [g/L]	2.7.8.7:Holo-[acyl-carrier-protein] synthase		0,669539011
IVT	Acetic Ac [g/L]	4.2.1.47:GDP-mannose 4,6-dehydratase		0,668376147
IVT	Propionic Ac [g/L]	2.4.2.29:tRNA-guanine(34) transglycosylase		0,668135811
IVV	Butyric Ac [g/L]	1.15.1.1:Superoxide dismutase		0,667337284
IVT	Acetic Ac [g/L]	1.7.99.1:Hydroxylamine reductase		0,666352176
IVT	Propionic Ac [g/L]	2.3.1.179:Beta-ketoacyl-[acyl-carrier-protein] synthase II		0,666295513
IVV	Acetic Ac [g/L]	1.1.1.132:GDP-mannose 6-dehydrogenase		0,665933053
IVV	Propionic Ac [g/L]	3.6.3.14:Transferred entry 7.1.2.2		0,665698919
IVV	Acetic Ac [g/L]	2.7.2.1:Acetate kinase		0,664233744
IVT	Acetic Ac [g/L]	5.3.1.16:isomerase		0,663516969
IVV	Propionic Ac [g/L]	6.1.1.3:Threonine--tRNA ligase		0,660852044
IVT	Acetic Ac [g/L]	2.7.7.23:UDP-N-acetylglucosamine diphosphorylase		0,65979264
IVV	Propionic Ac [g/L]	1.17.1.9:Formate dehydrogenase		0,65899979
IVV	Propionic Ac [g/L]	2.7.7.87:L-threonylcarbamoyladenylate synthase		0,658264414
IVT	Propionic Ac [g/L]	1.2.1.41:Glutamate-5-semialdehyde dehydrogenase		0,657349443
IVT	Acetic Ac [g/L]	1.3.5.1:Succinate dehydrogenase (quinone)		0,656307317
IVV	Butyric Ac [g/L]	5.1.3.2:UDP-glucose 4-epimerase		0,654481921
IVV	Butyric Ac [g/L]	2.4.1.20:Cellobiose phosphorylase		0,654304442
IVV	Propionic Ac [g/L]	2.7.4.1:Polyphosphate kinase		0,654190135
IVT	Acetic Ac [g/L]	3.5.1.10:Formyltetrahydrofolate deformylase		0,653834288
IVV	Propionic Ac [g/L]	3.5.2.3:Dihydroorotase		0,652984861
IVV	Butyric Ac [g/L]	6.1.1.3:Threonine--tRNA ligase		0,652087966
IVV	Butyric Ac [g/L]	1.3.1.12:Prephenate dehydrogenase		0,651474702
IVV	Butyric Ac [g/L]	1.17.7.1:(E)-4-hydroxy-3-methylbut-2-enyl-diphosphate synthase (ferredoxin)		0,650567981
IVV	Butyric Ac [g/L]	3.1.11.2:Exodeoxyribonuclease III		0,65031581
IVV	Butyric Ac [g/L]	2.5.1.3:Thiamine phosphate synthase		0,650295629
IVT	Acetic Ac [g/L]	2.7.4.1:Polyphosphate kinase		0,650290259
IVV	Butyric Ac [g/L]	2.1.1.14:5-methyltetrahydropteroyltriglutamate--homocysteine S-methyltransferase		0,650172941
IVV	Propionic Ac [g/L]	3.2.1.74:Glucan 1,4-beta-glucosidase		0,647375079
IVV	Acetic Ac [g/L]	6.1.1.12:Aspartate--tRNA ligase		0,647165215
IVV	Butyric Ac [g/L]	3.6.3.14:Transferred entry 7.1.2.2		0,646817872
IVV	Butyric Ac [g/L]	3.1.3.5:5'-nucleotidase		0,64666123
IVT	Propionic Ac [g/L]	2.4.1.18:1,4-alpha-glucan branching enzyme		0,645909302
IVT	Propionic Ac [g/L]	2.7.7.9:UTP--glucose-1-phosphate uridylyltransferase		0,645693451
IVV	Butyric Ac [g/L]	2.5.1.78:6,7-dimethyl-8-ribityllumazine synthase		0,645652726
IVT	Propionic Ac [g/L]	3.6.5.n1:Elongation factor 4		0,645526639
IVV	Acetic Ac [g/L]	2.6.1.9:Histidinol-phosphate transaminase		0,645262291
IVT	Acetic Ac [g/L]	3.2.1.89:Arabinogalactan endo-beta-1,4-galactanase		0,644971165
IVV	Butyric Ac [g/L]	6.2.1.5:Succinate--CoA ligase (ADP-forming)		0,644712599
IVV	Acetic Ac [g/L]	2.1.1.182:16S rRNA (adenine(1518)-N(6)/adenine(1519)-N(6))-dimethyltransferase		0,643945867
IVV	Butyric Ac [g/L]	3.2.1.17:Lysozyme		0,643469226

IVV	Acetic Ac [g/L]	4.1.3.27:Anthranilate synthase		0,642533522
IVV	Butyric Ac [g/L]	1.3.3.4:Protoporphyrinogen oxidase		0,642381786
IVT	Acetic Ac [g/L]	4.1.1.37:Uroporphyrinogen decarboxylase		0,641848565
IVT	Acetic Ac [g/L]	1.4.1.4:Glutamate dehydrogenase (NADP(+))		0,641445294
IVT	Propionic Ac [g/L]	1.2.7.8:Indolepyruvate ferredoxin oxidoreductase		0,641131752
IVT	Propionic Ac [g/L]	6.1.1.21:Histidine--tRNA ligase		0,639397074
IVT	Propionic Ac [g/L]	6.1.1.6:Lysine--tRNA ligase		0,637433959
IVV	Propionic Ac [g/L]	6.3.2.4:D-alanine--D-alanine ligase		0,636986355
IVV	Butyric Ac [g/L]	4.1.1.20:Diaminopimelate decarboxylase		0,636850445
IVV	Propionic Ac [g/L]	2.2.1.2:Transaldolase		0,635600072
IVT	Acetic Ac [g/L]	2.4.2.14:Amidophosphoribosyltransferase		0,635545818
IVV	Butyric Ac [g/L]	4.2.1.59:3-hydroxyacyl-[acyl-carrier-protein] dehydratase		0,635533315
IVT	Propionic Ac [g/L]	6.3.4.6:Urea carboxylase		0,635321752
IVV	Propionic Ac [g/L]	7.2.1.1:NADH:ubiquinone reductase (Na(+)-transporting)		0,635053419
IVT	Acetic Ac [g/L]	2.7.1.17:Xylulokinase		0,634992338
IVV	Butyric Ac [g/L]	4.2.1.75:Uroporphyrinogen-III synthase		0,634889609
IVT	Propionic Ac [g/L]	2.1.1.14:5-methyltetrahydropteroyltriglutamate--homocysteine S-methyltransferase		0,634747709
IVV	Acetic Ac [g/L]	2.7.9.1:Pyruvate, phosphate dikinase		0,634106328
IVT	Propionic Ac [g/L]	2.6.1.1:Aspartate transaminase		0,633805177
IVT	Acetic Ac [g/L]	6.3.1.5:NAD(+) synthase		0,633573987
IVV	Acetic Ac [g/L]	2.5.1.75:tRNA dimethylallyltransferase		0,632231224
IVT	Acetic Ac [g/L]	6.2.1.1:Acetate--CoA ligase		0,631657843
IVV	Butyric Ac [g/L]	3.5.4.3:Guanine deaminase		0,631587202
IVV	Propionic Ac [g/L]	1.2.1.12:Glyceraldehyde-3-phosphate dehydrogenase (phosphorylating)		0,631185875
IVT	Acetic Ac [g/L]	2.7.7.56:tRNA nucleotidyltransferase		0,631005059
IVT	Acetic Ac [g/L]	2.5.1.74:1,4-dihydroxy-2-naphthoate polyprenyltransferase		0,630611815
IVT	Acetic Ac [g/L]	3.1.26.3:Ribonuclease III		0,629907151
IVV	Propionic Ac [g/L]	3.5.4.3:Guanine deaminase		0,629479964
IVV	Propionic Ac [g/L]	1.1.1.44:Phosphogluconate dehydrogenase (NADP(+)-dependent, decarboxylating)		0,627791789
IVT	Acetic Ac [g/L]	1.2.4.2:Oxoglutarate dehydrogenase (succinyl-transferring)		0,627250004
IVV	Acetic Ac [g/L]	2.4.2.10:Orotate phosphoribosyltransferase		0,625283615
IVV	Propionic Ac [g/L]	2.7.1.71:Shikimate kinase		0,622671392
IVV	Butyric Ac [g/L]	6.3.5.4:Asparagine synthase (glutamine-hydrolyzing)		0,622107486
IVV	Butyric Ac [g/L]	2.3.1.79:Maltose O-acetyltransferase		0,621788878
IVT	Acetic Ac [g/L]	2.3.1.1:Amino-acid N-acetyltransferase		0,619843466
IVV	Propionic Ac [g/L]	3.4.25.2:HslU--HslV peptidase		0,618970302
IVV	Acetic Ac [g/L]	3.4.13.22:D-Ala-D-Ala dipeptidase		0,616587035
IVV	Butyric Ac [g/L]	1.11.1.1:NADH peroxidase		0,616071939
IVV	Butyric Ac [g/L]	2.7.1.180:FAD:protein FMN transferase		0,614571941
IVV	Butyric Ac [g/L]	3.2.1.14:Chitinase		0,613475615
IVV	Butyric Ac [g/L]	3.6.1.7:Acylphosphatase		0,613475615
IVV	Butyric Ac [g/L]	4.6.1.1:Adenylate cyclase		0,613475615
IVV	Butyric Ac [g/L]	6.3.4.15:Biotin--[biotin carboxyl-carrier protein] ligase		0,613475615
IVV	Butyric Ac [g/L]	1.3.5.4:Fumarate reductase (quinol)		0,613475615
IVV	Butyric Ac [g/L]	2.1.1.34:tRNA (guanosine(18)-2'-O)-methyltransferase		0,613475615
IVV	Butyric Ac [g/L]	2.3.1.1:Amino-acid N-acetyltransferase		0,613475615
IVV	Butyric Ac [g/L]	2.6.1.62:Adenosylmethionine--8-amino-7-oxononanoate transaminase		0,613475615
IVV	Butyric Ac [g/L]	2.6.1.39:2-aminoadipate transaminase		0,613475615
IVV	Butyric Ac [g/L]	3.1.1.72:Acetylxylan esterase		0,613475615
IVV	Butyric Ac [g/L]	3.2.1.26:Beta-fructofuranosidase		0,613475615
IVV	Butyric Ac [g/L]	3.4.15.5:Peptidyl-dipeptidase Dcp		0,613475615
IVV	Butyric Ac [g/L]	5.1.3.20:ADP-glyceromanno-heptose 6-epimerase		0,613475615
IVV	Butyric Ac [g/L]	3.5.1.88:Peptide deformylase		0,612643925
IVV	Butyric Ac [g/L]	6.3.2.9:UDP-N-acetylmuramoyl-L-alanine--D-glutamate ligase		0,611158015
IVT	Acetic Ac [g/L]	2.5.1.78:6,7-dimethyl-8-ribityllumazine synthase		0,611124319
IVV	Acetic Ac [g/L]	2.1.1.199:16S rRNA (cytosine(1402)-N(4))-methyltransferase		0,611095911
IVV	Acetic Ac [g/L]	2.6.1.11:Acetylornithine transaminase		0,610265977
IVV	Acetic Ac [g/L]	1.1.1.58:Tagaturonate reductase		0,609902468
IVV	Propionic Ac [g/L]	1.1.1.58:Tagaturonate reductase		0,609442447
IVT	Propionic Ac [g/L]	2.7.7.24:Glucose-1-phosphate thymidylyltransferase		0,609005171
IVT	Propionic Ac [g/L]	5.4.2.11:Phosphoglycerate mutase (2,3-diphosphoglycerate-dependent)		0,608535763
IVV	Propionic Ac [g/L]	2.1.2.11:3-methyl-2-oxobutanoate hydroxymethyltransferase		0,608138502
IVV	Acetic Ac [g/L]	5.1.1.3:Glutamate racemase		0,607532338
IVV	Propionic Ac [g/L]	2.7.1.5:Rhamnulokinase		0,606145266
IVT	Propionic Ac [g/L]	1.7.1.13:PreQ(1) synthase		0,605890923
IVV	Butyric Ac [g/L]	2.5.1.19:3-phosphoshikimate 1-carboxyvinyltransferase		0,604923316
IVV	Propionic Ac [g/L]	2.7.1.156:Adenosylcobinamide kinase		0,603471255
IVV	Propionic Ac [g/L]	2.7.7.62:Adenosylcobinamide-phosphate guanylyltransferase		0,603471255
IVT	Propionic Ac [g/L]	3.2.1.4:Cellulase		0,602028577
IVV	Propionic Ac [g/L]	2.6.1.9:Histidinol-phosphate transaminase		0,600950111
IVV	Acetic Ac [g/L]	2.5.1.15:Dihydropteroate synthase		0,599987156
IVT	Acetic Ac [g/L]	2.4.2.8:Hypoxanthine phosphoribosyltransferase		0,599844995
IVT	Acetic Ac [g/L]	1.1.1.3:Homoserine dehydrogenase		0,59947348
IVT	Acetic Ac [g/L]	6.3.5.7:Glutaminyl-tRNA synthase (glutamine-hydrolyzing)		0,599233115
IVV	Butyric Ac [g/L]	4.2.3.3:Methylglyoxal synthase		0,598519968
IVT	Acetic Ac [g/L]	1.5.1.20:Methylenetetrahydrofolate reductase (NAD(P)H)		0,597900872
IVT	Propionic Ac [g/L]	6.1.1.18:Glutamine--tRNA ligase		0,597147427
IVT	Acetic Ac [g/L]	2.7.7.18:Nicotinate-nucleotide adenylyltransferase		0,595505098
IVV	Propionic Ac [g/L]	6.3.4.21:Nicotinate phosphoribosyltransferase		0,594460233
IVV	Acetic Ac [g/L]	3.4.21.88:Repressor LexA		0,592908706
IVV	Acetic Ac [g/L]	4.1.1.48:Indole-3-glycerol-phosphate synthase		0,591660513
IVV	Propionic Ac [g/L]	2.3.1.28:Chloramphenicol O-acetyltransferase		0,590273328
IVV	Acetic Ac [g/L]	6.3.2.4:D-alanine--D-alanine ligase		0,588710979

IVV	Propionic Ac [g/L]	2.8.1.10:Thiazole synthase		0,587463797
IVV	Acetic Ac [g/L]	3.2.1.37:Xylan 1,4-beta-xylosidase		0,586971004
IVV	Butyric Ac [g/L]	1.17.7.4:4-hydroxy-3-methylbut-2-enyl diphosphate reductase		0,585307484
IVV	Butyric Ac [g/L]	2.1.1.148:Thymidylate synthase (FAD)		0,585026334
IVT	Acetic Ac [g/L]	2.5.1.19:3-phosphoshikimate 1-carboxyvinyltransferase		0,584979733
IVV	Butyric Ac [g/L]	6.2.1.30:Phenylacetate--CoA ligase		0,584892497
IVT	Acetic Ac [g/L]	2.6.1.52:Phosphoserine transaminase		0,584438089
IVT	Acetic Ac [g/L]	2.1.3.3:Ornithine carbamoyltransferase		0,583074687
IVV	Acetic Ac [g/L]	3.4.21.53:Endopeptidase La		0,580693523
IVT	Acetic Ac [g/L]	2.3.1.28:Chloramphenicol O-acetyltransferase		0,578245064
IVV	Propionic Ac [g/L]	1.3.3.4:Protoporphyrinogen oxidase		0,578058525
IVV	Propionic Ac [g/L]	2.7.7.85:Diadenylate cyclase		0,577422773
IVV	Butyric Ac [g/L]	2.1.1.195:Cobalt-precorrin-5B (C(1))-methyltransferase		0,576165191
IVV	Butyric Ac [g/L]	2.4.2.22:Xanthine phosphoribosyltransferase		0,574919546
IVT	Acetic Ac [g/L]	6.3.4.21:Nicotinate phosphoribosyltransferase		0,574740446
IVV	Acetic Ac [g/L]	3.2.1.55:Non-reducing end alpha-L-arabinofuranosidase		0,573543035
IVV	Acetic Ac [g/L]	1.7.99.1:Hydroxylamine reductase		0,573172502
IVV	Propionic Ac [g/L]	3.1.3.11:Fructose-bisphosphatase		0,57281708
IVV	Propionic Ac [g/L]	3.2.1.135:Neopullulanase		0,570464157
IVT	Acetic Ac [g/L]	2.4.1.129:Peptidoglycan glycosyltransferase		0,57010188
IVT	Acetic Ac [g/L]	3.6.3.31:Transferred entry 7.6.2.11		0,569684006
IVV	Acetic Ac [g/L]	3.2.1.22:Alpha-galactosidase		0,569471737
IVT	Acetic Ac [g/L]	2.2.1.2:Transaldolase		0,56888836
IVV	Acetic Ac [g/L]	3.1.26.11:Ribonuclease Z		0,568837948
IVV	Propionic Ac [g/L]	3.2.1.183:UDP-N-acetylglucosamine 2-epimerase (hydrolyzing)		0,568431481
IVT	Propionic Ac [g/L]	1.8.1.4:Dihydrolipoyl dehydrogenase		0,566402996
IVT	Propionic Ac [g/L]	1.1.1.103:L-threonine 3-dehydrogenase		0,565714984
IVV	Propionic Ac [g/L]	2.7.1.130:Tetraacyldisaccharide 4'-kinase		0,565541606
IVV	Butyric Ac [g/L]	5.4.99.2:Methylmalonyl-CoA mutase		0,56511296
IVV	Butyric Ac [g/L]	4.2.99.18:DNA-(apurinic or apyrimidinic site) lyase		0,564423044
IVV	Butyric Ac [g/L]	2.7.1.26:Riboflavin kinase		0,563034035
IVV	Butyric Ac [g/L]	2.7.7.2:FAD synthase		0,563034035
IVV	Propionic Ac [g/L]	6.2.1.5:Succinate--CoA ligase (ADP-forming)		0,562207427
IVT	Propionic Ac [g/L]	1.2.1.70:Glutamyl-tRNA reductase		0,560638248
IVV	Butyric Ac [g/L]	1.5.1.2:Pyrroline-5-carboxylate reductase		0,559545885
IVV	Acetic Ac [g/L]	4.2.1.1:Carbonic anhydrase		0,559088338
IVV	Propionic Ac [g/L]	2.7.7.7:DNA-directed DNA polymerase		0,553953553
IVV	Butyric Ac [g/L]	2.5.1.55:3-deoxy-8-phosphooctulonate synthase		0,552910753
IVT	Propionic Ac [g/L]	1.3.5.1:Succinate dehydrogenase (quinone)		0,551665591
IVT	Acetic Ac [g/L]	1.1.1.95:Phosphoglycerate dehydrogenase		0,548463197
IVV	Butyric Ac [g/L]	3.5.1.2:Glutaminase		0,548063476
IVV	Butyric Ac [g/L]	2.7.2.3:Phosphoglycerate kinase		0,546825817
IVT	Acetic Ac [g/L]	5.3.1.9:Glucose-6-phosphate isomerase		0,546681762
IVV	Acetic Ac [g/L]	2.7.1.90:Diphosphate--fructose-6-phosphate 1-phosphotransferase		0,546292824
IVT	Acetic Ac [g/L]	2.6.99.2:Pyridoxine 5'-phosphate synthase		0,546271488
IVV	Propionic Ac [g/L]	3.1.3.5:5'-nucleotidase		0,546030506
IVT	Propionic Ac [g/L]	2.1.1.177:23S rRNA (pseudouridine(1915)-N(3))-methyltransferase		0,545981315
IVT	Acetic Ac [g/L]	2.3.1.18:Galactoside O-acetyltransferase		0,545750033
IVT	Acetic Ac [g/L]	6.3.4.5:Argininosuccinate synthase		0,545729056
IVV	Propionic Ac [g/L]	2.5.1.6:Methionine adenosyltransferase		0,545667831
IVT	Propionic Ac [g/L]	3.6.1.31:Phosphoribosyl-ATP diphosphatase		0,545449676
IVV	Acetic Ac [g/L]	6.1.1.17:Glutamate--tRNA ligase		0,544340622
IVT	Propionic Ac [g/L]	2.7.4.6:Nucleoside-diphosphate kinase		0,541211663
IVV	Butyric Ac [g/L]	5.1.3.3:Aldose 1-epimerase		0,540542791
IVV	Propionic Ac [g/L]	1.1.1.132:GDP-mannose 6-dehydrogenase		0,539547481
IVT	Acetic Ac [g/L]	2.7.7.87:L-threonylcarbamoyladenylate synthase		0,539310611
IVT	Propionic Ac [g/L]	3.4.11.5:Prolyl aminopeptidase		0,538122131
IVV	Butyric Ac [g/L]	2.6.1.42:Branched-chain-amino-acid transaminase		0,537458099
IVT	Acetic Ac [g/L]	2.7.7.9:UTP--glucose-1-phosphate uridylyltransferase		0,536866336
IVV	Acetic Ac [g/L]	2.8.1.13:tRNA-uridine 2-sulfurtransferase		0,536204513
IVV	Acetic Ac [g/L]	2.7.1.180:FAD:protein FMN transferase		0,536038496
IVV	Propionic Ac [g/L]	2.5.1.78:6,7-dimethyl-8-ribityllumazine synthase		0,534632981
IVV	Propionic Ac [g/L]	2.7.1.2:Glucokinase		0,534438871
IVT	Propionic Ac [g/L]	1.1.1.132:GDP-mannose 6-dehydrogenase		0,533796646
IVT	Propionic Ac [g/L]	6.3.3.1:Phosphoribosylformylglycinamidine cyclo-ligase		0,533512148
IVT	Acetic Ac [g/L]	2.7.1.24:Dephospho-CoA kinase		0,532614395
IVT	Propionic Ac [g/L]	2.7.2.4:Aspartate kinase		0,528615035
IVV	Butyric Ac [g/L]	2.7.13.3:Histidine kinase		0,528486562
IVV	Acetic Ac [g/L]	2.7.1.11:6-phosphofructokinase		0,526923551
IVT	Acetic Ac [g/L]	3.1.11.6:Exodeoxyribonuclease VII		0,526068418
IVV	Acetic Ac [g/L]	3.5.4.16:GTP cyclohydrolase I		0,525852385
IVV	Propionic Ac [g/L]	2.5.1.1:Dimethylallyltranstransferase		0,525844588
IVV	Propionic Ac [g/L]	2.5.1.10:(2E,6E)-farnesyl diphosphate synthase		0,525844588
IVT	Acetic Ac [g/L]	6.3.5.1:NAD(+) synthase (glutamine-hydrolyzing)		0,525738237
IVT	Acetic Ac [g/L]	2.7.7.85:Diadenylate cyclase		0,523402178
IVV	Butyric Ac [g/L]	2.1.1.182:16S rRNA (adenine(1518)-N(6)/adenine(1519)-N(6))-dimethyltransferase		0,523198713
IVV	Butyric Ac [g/L]	2.1.2.11:3-methyl-2-oxobutanoate hydroxymethyltransferase		0,522027532
IVV	Acetic Ac [g/L]	4.2.1.51:Prephenate dehydratase		0,522027072
IVT	Propionic Ac [g/L]	3.6.5.3:Protein-synthesizing GTPase		0,520483196
IVV	Butyric Ac [g/L]	2.5.1.47:Cysteine synthase		0,520025018
IVV	Acetic Ac [g/L]	2.7.7.85:Diadenylate cyclase		0,519102557
IVV	Propionic Ac [g/L]	3.1.11.6:Exodeoxyribonuclease VII		0,518604123
IVT	Propionic Ac [g/L]	2.3.3.1:Citrate (Si)-synthase		0,517847991

IVT	Acetic Ac [g/L]	2.7.7.60:2-C-methyl-D-erythritol 4-phosphate cytidylyltransferase		0,515366192
IVV	Acetic Ac [g/L]	4.2.1.20:Tryptophan synthase		0,514680329
IVV	Acetic Ac [g/L]	2.7.1.92:5-dehydro-2-deoxygluconokinase		0,514235863
IVV	Butyric Ac [g/L]	3.2.2.9:Adenosylhomocysteine nucleosidase		0,513624641
IVV	Butyric Ac [g/L]	2.3.1.54:Formate C-acetyltransferase		0,512944235
IVV	Butyric Ac [g/L]	4.2.1.126:N-acetylmuramic acid 6-phosphate etherase		0,512309014
IVV	Propionic Ac [g/L]	4.2.1.33:3-isopropylmalate dehydratase		0,512198641
IVT	Acetic Ac [g/L]	2.7.9.1:Pyruvate, phosphate dikinase		0,512184161
IVV	Butyric Ac [g/L]	2.1.2.9:Methionyl-tRNA formyltransferase		0,510342273
IVT	Acetic Ac [g/L]	2.7.1.33:Pantothenate kinase		0,510027333
IVT	Propionic Ac [g/L]	3.5.4.10:IMP cyclohydrolase		0,509897687
IVT	Acetic Ac [g/L]	3.2.1.23:Beta-galactosidase		0,508111242
IVV	Propionic Ac [g/L]	4.3.2.1:Argininosuccinate lyase		0,505423661
IVV	Butyric Ac [g/L]	4.2.1.19:Imidazoleglycerol-phosphate dehydratase		0,50472378
IVV	Acetic Ac [g/L]	2.3.1.30:Serine O-acetyltransferase		0,504306752
IVV	Propionic Ac [g/L]	2.7.2.3:Phosphoglycerate kinase		0,503924232
IVV	Butyric Ac [g/L]	2.4.1.281:4-O-beta-D-mannosyl-D-glucose phosphorylase		0,503764113
IVT	Acetic Ac [g/L]	3.5.1.88:Peptide deformylase		0,50241014
IVV	Butyric Ac [g/L]	3.5.4.33:tRNA(adenine(34)) deaminase		0,501565941
IVV	Propionic Ac [g/L]	1.1.1.133:dTDP-4-dehydrorhamnose reductase		0,501499027
IVT	Propionic Ac [g/L]	2.4.2.8:Hypoxanthine phosphoribosyltransferase		0,501362755
IVT	Propionic Ac [g/L]	1.5.1.5:Methylenetetrahydrofolate dehydrogenase (NADP(+))		0,501190268
IVT	Propionic Ac [g/L]	3.3.1.1:Adenosylhomocysteinase		0,500415225
IVV	Butyric Ac [g/L]	2.1.1.193:16S rRNA (uracil(1498)-N(3))-methyltransferase		0,499111936
IVV	Propionic Ac [g/L]	1.11.1.15:Peroxiredoxin		0,498886
IVT	Acetic Ac [g/L]	4.2.99.18:DNA-(apurinic or apyrimidinic site) lyase		0,497988217
IVV	Propionic Ac [g/L]	2.6.1.11:Acetylornithine transaminase		0,497754081
IVV	Acetic Ac [g/L]	2.7.1.26:Riboflavin kinase		0,496474033
IVV	Acetic Ac [g/L]	2.7.7.2:FAD synthase		0,496474033
IVT	Propionic Ac [g/L]	5.3.1.17:5-dehydro-4-deoxy-D-glucuronate isomerase		0,496073731
IVT	Propionic Ac [g/L]	6.3.2.4:D-alanine--D-alanine ligase		0,49566966
IVV	Propionic Ac [g/L]	2.7.3.9:Phosphoenolpyruvate--protein phosphotransferase		0,495428033
IVV	Butyric Ac [g/L]	5.3.1.5:Xylose isomerase		0,495268863
IVV	Butyric Ac [g/L]	1.17.7.3:(E)-4-hydroxy-3-methylbut-2-enyl-diphosphate synthase (flavodoxin)		0,494849395
IVV	Butyric Ac [g/L]	4.2.1.2:Fumarate hydratase		0,493926797
IVV	Acetic Ac [g/L]	5.3.1.24:Phosphoribosylanthranilate isomerase		0,493559303
IVV	Acetic Ac [g/L]	2.4.2.17:ATP phosphoribosyltransferase		0,493230673
IVV	Propionic Ac [g/L]	3.1.11.2:Exodeoxyribonuclease III		0,492398648
IVT	Propionic Ac [g/L]	6.3.4.5:Argininosuccinate synthase		0,492205504
IVV	Propionic Ac [g/L]	1.3.1.98:UDP-N-acetylmuramate dehydrogenase		0,490203905
IVV	Propionic Ac [g/L]	2.5.1.7:UDP-N-acetylglucosamine 1-carboxyvinyltransferase		0,488880667
IVV	Propionic Ac [g/L]	5.1.1.3:Glutamate racemase		0,488489306
IVT	Propionic Ac [g/L]	3.4.21.92:Endopeptidase Clp		0,487067823
IVT	Acetic Ac [g/L]	3.4.13.22:D-Ala-D-Ala dipeptidase		0,486571463
IVT	Propionic Ac [g/L]	7.1.2.2:H(+)-transporting two-sector ATPase		0,485822232
IVV	Butyric Ac [g/L]	4.3.2.10:Imidazole glycerol-phosphate synthase		0,48572243
IVT	Propionic Ac [g/L]	6.1.1.22:Asparagine--tRNA ligase		0,483162943
IVV	Propionic Ac [g/L]	2.5.1.74:1,4-dihydroxy-2-naphthoate polyprenyltransferase		0,48268504
IVV	Propionic Ac [g/L]	1.17.7.1:(E)-4-hydroxy-3-methylbut-2-enyl-diphosphate synthase (ferredoxin)		0,481963522
IVT	Acetic Ac [g/L]	5.1.1.7:Diaminopimelate epimerase		0,480724913
IVV	Propionic Ac [g/L]	2.7.1.148:4-(cytidine 5'-diphospho)-2-C-methyl-D-erythritol kinase		0,480514921
IVT	Propionic Ac [g/L]	2.6.1.16:Glutamine--fructose-6-phosphate transaminase (isomerizing)		0,480342814
IVT	Acetic Ac [g/L]	1.3.98.3:Coproporphyrinogen dehydrogenase		0,479904713
IVV	Acetic Ac [g/L]	2.6.1.42:Branched-chain-amino-acid transaminase		0,479843053
IVT	Propionic Ac [g/L]	1.1.1.69:Gluconate 5-dehydrogenase		0,479801987
IVT	Acetic Ac [g/L]	2.1.2.10:Aminomethyltransferase		0,477495487
IVT	Propionic Ac [g/L]	1.2.1.12:Glyceraldehyde-3-phosphate dehydrogenase (phosphorylating)		0,475852423
IVT	Propionic Ac [g/L]	2.7.4.1:Polyphosphate kinase		0,474220093
IVT	Propionic Ac [g/L]	2.4.2.3:Uridine phosphorylase		0,474168037
IVV	Propionic Ac [g/L]	4.2.1.8:Mannonate dehydratase		0,474154197
IVV	Butyric Ac [g/L]	4.1.3.27:Anthranilate synthase		0,474022893
IVV	Propionic Ac [g/L]	2.3.1.n2:Transferred entry 2.3.1.274		0,473569069
IVV	Acetic Ac [g/L]	2.1.1.37:DNA (cytosine-5-)-methyltransferase		0,472472885
IVT	Propionic Ac [g/L]	3.5.4.16:GTP cyclohydrolase I		0,470338678
IVT	Propionic Ac [g/L]	6.1.1.1:Tyrosine--tRNA ligase		0,470000323
IVV	Acetic Ac [g/L]	2.7.4.3:Adenylate kinase		0,469651852
IVV	Acetic Ac [g/L]	1.1.1.3:Homoserine dehydrogenase		0,466527325
IVT	Acetic Ac [g/L]	6.1.1.21:Histidine--tRNA ligase		0,46568236
IVT	Propionic Ac [g/L]	3.2.1.131:Xylan alpha-1,2-glucuronosidase		0,465402116
IVV	Butyric Ac [g/L]	4.2.1.9:Dihydroxy-acid dehydratase		0,463969908
IVV	Propionic Ac [g/L]	6.2.1.30:Phenylacetate--CoA ligase		0,463945983
IVT	Acetic Ac [g/L]	5.3.1.1:Triose-phosphate isomerase		0,46278442
IVT	Propionic Ac [g/L]	3.5.99.6:Glucosamine-6-phosphate deaminase		0,460506948
IVV	Acetic Ac [g/L]	2.6.1.1:Aspartate transaminase		0,459988389
IVV	Acetic Ac [g/L]	2.5.1.9:Riboflavin synthase		0,459500737
IVT	Propionic Ac [g/L]	6.3.2.8:UDP-N-acetylmuramate--L-alanine ligase		0,459423141
IVV	Acetic Ac [g/L]	4.2.1.2:Fumarate hydratase		0,457387607
IVT	Acetic Ac [g/L]	3.1.21.3:Type I site-specific deoxyribonuclease		0,456342946
IVT	Propionic Ac [g/L]	2.4.2.22:Xanthine phosphoribosyltransferase		0,455218106
IVV	Propionic Ac [g/L]	6.3.2.9:UDP-N-acetylmuramoyl-L-alanine--D-glutamate ligase		0,454786362
IVV	Butyric Ac [g/L]	2.7.1.2:Glucokinase		0,451866256
IVV	Propionic Ac [g/L]	3.4.11.18:Methionyl aminopeptidase		0,451370934
IVV	Butyric Ac [g/L]	2.7.2.4:Aspartate kinase		0,450240961

IVT	Propionic Ac [g/L]	3.5.4.5:Cytidine deaminase		0,445685445
IVV	Butyric Ac [g/L]	1.97.1.4:[Formate-C-acetyltransferase]-activating enzyme		0,443435688
IVT	Acetic Ac [g/L]	4.1.2.50:6-carboxytetrahydropterin synthase		0,442473658
IVV	Propionic Ac [g/L]	2.5.1.3:Thiamine phosphate synthase		0,442375539
IVT	Acetic Ac [g/L]	5.3.1.28:D-sedoheptulose 7-phosphate isomerase		0,44056522
IVV	Butyric Ac [g/L]	2.1.1.33:tRNA (guanine(46)-N(7))-methyltransferase		0,440193161
IVV	Acetic Ac [g/L]	4.2.1.7:Altronate dehydratase		0,439560769
IVT	Acetic Ac [g/L]	2.2.1.9:synthase		0,439304493
IVV	Acetic Ac [g/L]	2.1.1.61:tRNA (5-methylaminomethyl-2-thiouridylate)-methyltransferase		0,438636765
IVV	Propionic Ac [g/L]	2.1.1.14:5-methyltetrahydropteroyltriglutamate--homocysteine S-methyltransferase		0,43860712
IVT	Acetic Ac [g/L]	4.3.3.7:4-hydroxy-tetrahydrodipicolinate synthase		0,435824578
IVT	Propionic Ac [g/L]	1.1.1.218:Morphine 6-dehydrogenase		0,435377134
IVT	Acetic Ac [g/L]	2.5.1.6:Methionine adenosyltransferase		0,435128427
IVT	Acetic Ac [g/L]	2.5.1.61:Hydroxymethylbilane synthase		0,434600386
IVV	Acetic Ac [g/L]	1.1.1.193:5-amino-6-(5-phosphoribosylamino)uracil reductase		0,433785444
IVV	Acetic Ac [g/L]	3.5.4.26:Diaminohydroxyphosphoribosylaminopyrimidine deaminase		0,433785444
IVT	Acetic Ac [g/L]	4.3.1.19:Threonine ammonia-lyase		0,433725691
IVV	Propionic Ac [g/L]	3.4.21.102:C-terminal processing peptidase		0,433479751
IVT	Acetic Ac [g/L]	5.4.99.12:tRNA pseudouridine(38-40) synthase		0,431460939
IVT	Propionic Ac [g/L]	1.11.1.1:NADH peroxidase		0,431145236
IVT	Propionic Ac [g/L]	1.17.4.1:Ribonucleoside-diphosphate reductase		0,431145236
IVT	Propionic Ac [g/L]	2.6.1.13:Ornithine aminotransferase		0,431145236
IVT	Propionic Ac [g/L]	1.1.1.157:3-hydroxybutyryl-CoA dehydrogenase		0,431145236
IVT	Propionic Ac [g/L]	1.1.1.17:Mannitol-1-phosphate 5-dehydrogenase		0,431145236
IVT	Propionic Ac [g/L]	1.2.1.79:Succinate-semialdehyde dehydrogenase (NADP(+))		0,431145236
IVT	Propionic Ac [g/L]	1.7.1.4:Nitrite reductase (NAD(P)H)		0,431145236
IVT	Propionic Ac [g/L]	2.1.1.173:23S rRNA (guanine(2445)-N(2))-methyltransferase		0,431145236
IVT	Propionic Ac [g/L]	2.3.1.183:Phosphinothricin acetyltransferase		0,431145236
IVT	Propionic Ac [g/L]	2.7.1.107:Diacylglycerol kinase (ATP)		0,431145236
IVT	Propionic Ac [g/L]	2.7.7.12:UDP-glucose--hexose-1-phosphate uridylyltransferase		0,431145236
IVT	Propionic Ac [g/L]	3.5.1.94:Gamma-glutamyl-gamma-aminobutyrate hydrolase		0,431145236
IVT	Propionic Ac [g/L]	4.1.3.1:Isocitrate lyase		0,431145236
IVT	Propionic Ac [g/L]	5.4.99.61:Precorrin-8X methylmutase		0,431145236
IVT	Propionic Ac [g/L]	6.3.1.8:Glutathionylspermidine synthase		0,431145236
IVT	Propionic Ac [g/L]	2.1.1.72:Site-specific DNA-methyltransferase (adenine-specific)		0,431145236
IVT	Propionic Ac [g/L]	2.7.1.144:Tagatose-6-phosphate kinase		0,431145236
IVT	Propionic Ac [g/L]	3.6.1.7:Acylphosphatase		0,431145236
IVT	Propionic Ac [g/L]	4.1.1.31:Phosphoenolpyruvate carboxylase		0,431145236
IVT	Propionic Ac [g/L]	2.7.1.50:Hydroxyethylthiazole kinase		0,431145236
IVT	Propionic Ac [g/L]	3.5.1.18:Succinyl-diaminopimelate desuccinylase		0,431145236
IVT	Propionic Ac [g/L]	3.6.3.3:Transferred entry 7.2.2.21		0,431145236
IVT	Propionic Ac [g/L]	2.4.2.15:Guanosine phosphorylase		0,431145236
IVT	Propionic Ac [g/L]	2.4.2.2:Pyrimidine-nucleoside phosphorylase		0,431145236
IVT	Propionic Ac [g/L]	5.1.3.23:UDP-2,3-diacetamido-2,3-dideoxyglucuronic acid 2-epimerase		0,431145236
IVT	Propionic Ac [g/L]	1.1.1.6:Glycerol dehydrogenase		0,431145236
IVT	Propionic Ac [g/L]	1.11.1.6:Catalase		0,431145236
IVT	Propionic Ac [g/L]	1.2.1.39:Phenylacetaldehyde dehydrogenase		0,431145236
IVT	Propionic Ac [g/L]	1.2.1.71:Succinylglutamate-semialdehyde dehydrogenase		0,431145236
IVT	Propionic Ac [g/L]	1.2.7.7:3-methyl-2-oxobutanoate dehydrogenase (ferredoxin)		0,431145236
IVT	Propionic Ac [g/L]	1.4.1.14:Glutamate synthase (NADH)		0,431145236
IVT	Propionic Ac [g/L]	1.4.3.5:Pyridoxal 5'-phosphate synthase		0,431145236
IVT	Propionic Ac [g/L]	1.4.99.6:D-arginine dehydrogenase		0,431145236
IVT	Propionic Ac [g/L]	1.8.1.7:Glutathione-disulfide reductase		0,431145236
IVT	Propionic Ac [g/L]	2.1.1.171:16S rRNA (guanine(966)-N(2))-methyltransferase		0,431145236
IVT	Propionic Ac [g/L]	2.1.1.186:23S rRNA (cytidine(2498)-2'-O)-methyltransferase		0,431145236
IVT	Propionic Ac [g/L]	2.1.1.190:23S rRNA (uracil(1939)-C(5))-methyltransferase		0,431145236
IVT	Propionic Ac [g/L]	2.1.1.264:23S rRNA (guanine(2069)-N(7))-methyltransferase		0,431145236
IVT	Propionic Ac [g/L]	2.1.1.35:tRNA (uracil(54)-C(5))-methyltransferase		0,431145236
IVT	Propionic Ac [g/L]	2.3.1.118:N-hydroxyarylamine O-acetyltransferase		0,431145236
IVT	Propionic Ac [g/L]	2.7.14.1:Protein arginine kinase		0,431145236
IVT	Propionic Ac [g/L]	2.7.7.59:[Protein-PII] uridylyltransferase		0,431145236
IVT	Propionic Ac [g/L]	2.7.8.5:CDP-diacylglycerol--glycerol-3-phosphate 1-phosphatidyltransferase		0,431145236
IVT	Propionic Ac [g/L]	2.7.9.3:Selenide, water dikinase		0,431145236
IVT	Propionic Ac [g/L]	3.1.21.7:Deoxyribonuclease V		0,431145236
IVT	Propionic Ac [g/L]	3.1.7.2:Guanosine-3',5'-bis(diphosphate) 3'-diphosphatase		0,431145236
IVT	Propionic Ac [g/L]	3.2.1.170:Mannosylglycerate hydrolase		0,431145236
IVT	Propionic Ac [g/L]	3.4.13.9:Xaa-Pro dipeptidase		0,431145236
IVT	Propionic Ac [g/L]	3.5.4.12:dCMP deaminase		0,431145236
IVT	Propionic Ac [g/L]	3.6.1.41:Bis(5'-nucleosyl)-tetraphosphatase (symmetrical)		0,431145236
IVT	Propionic Ac [g/L]	3.6.3.19:Transferred entry 7.5.2.1		0,431145236
IVT	Propionic Ac [g/L]	4.2.1.42:Galactarate dehydratase		0,431145236
IVT	Propionic Ac [g/L]	4.2.2.n2:Peptidoglycan lytic endotransglycosylase		0,431145236
IVT	Propionic Ac [g/L]	4.3.1.2:Methylaspartate ammonia-lyase		0,431145236
IVT	Propionic Ac [g/L]	4.99.1.12:Pyridinium-3,5-bisthiocarboxylic acid mononucleotide nickel chelatase		0,431145236
IVT	Propionic Ac [g/L]	5.1.3.7:UDP-N-acetylglucosamine 4-epimerase		0,431145236
IVT	Propionic Ac [g/L]	6.2.1.17:Propionate--CoA ligase		0,431145236
IVT	Propionic Ac [g/L]	7.1.1.1:Proton-translocating NAD(P)(+) transhydrogenase		0,431145236
IVT	Propionic Ac [g/L]	3.2.1.70:Glucan 1,6-alpha-glucosidase		0,431145236
IVT	Propionic Ac [g/L]	6.3.1.1:Aspartate--ammonia ligase		0,431019076
IVT	Propionic Ac [g/L]	6.1.1.4:Leucine--tRNA ligase		0,428780594
IVT	Acetic Ac [g/L]	2.1.2.1:Glycine hydroxymethyltransferase		0,427985588
IVV	Acetic Ac [g/L]	2.1.3.2:Aspartate carbamoyltransferase		0,426151176
IVV	Acetic Ac [g/L]	2.5.1.55:3-deoxy-8-phosphooctulonate synthase		0,425739102

IVV	Acetic Ac [g/L]	1.17.99.6:Epoxyqueuosine reductase		0,424667694
IVV	Propionic Ac [g/L]	1.4.1.13:Glutamate synthase (NADPH)		0,424479055
IVT	Acetic Ac [g/L]	5.4.99.25:tRNA pseudouridine(55) synthase		0,424443872
IVV	Acetic Ac [g/L]	2.5.1.47:Cysteine synthase		0,423878765
IVV	Butyric Ac [g/L]	5.1.1.1:Alanine racemase		0,422983489
IVV	Acetic Ac [g/L]	5.1.3.2:UDP-glucose 4-epimerase		0,422945818
IVT	Propionic Ac [g/L]	2.6.1.52:Phosphoserine transaminase		0,421987656
IVT	Acetic Ac [g/L]	6.1.1.20:Phenylalanine--tRNA ligase		0,421870706
IVT	Acetic Ac [g/L]	2.7.1.2:Glucokinase		0,418068984
IVT	Propionic Ac [g/L]	4.2.1.1:Carbonic anhydrase		0,416199312
IVV	Propionic Ac [g/L]	2.3.1.39:[Acyl-carrier-protein] S-malonyltransferase		0,416063501
IVV	Acetic Ac [g/L]	4.2.1.10:3-dehydroquinate dehydratase		0,415789293
IVV	Acetic Ac [g/L]	2.1.3.3:Ornithine carbamoyltransferase		0,415672887
IVV	Acetic Ac [g/L]	1.1.1.131:Mannuronate reductase		0,414746516
IVV	Butyric Ac [g/L]	3.1.3.3:Phosphoserine phosphatase		0,413558384
IVT	Acetic Ac [g/L]	3.4.14.4:Dipeptidyl-peptidase III		0,412969566
IVV	Butyric Ac [g/L]	2.5.1.6:Methionine adenosyltransferase		0,412938144
IVV	Propionic Ac [g/L]	3.2.1.37:Xylan 1,4-beta-xylosidase		0,412714766
IVV	Propionic Ac [g/L]	1.4.4.2:Glycine dehydrogenase (aminomethyl-transferring)		0,412170642
IVV	Acetic Ac [g/L]	1.1.1.271:GDP-L-fucose synthase		0,41184911
IVT	Propionic Ac [g/L]	2.8.4.3:tRNA-2-methylthio-N(6)-dimethylallyladenosine synthase		0,411842914
IVT	Propionic Ac [g/L]	6.3.5.3:Phosphoribosylformylglycinamidine synthase		0,4113828
IVT	Acetic Ac [g/L]	2.1.1.192:23S rRNA (adenine(2503)-C(2))-methyltransferase		0,409882441
IVV	Butyric Ac [g/L]	3.4.11.4:Tripeptide aminopeptidase		0,408933119
IVV	Propionic Ac [g/L]	4.1.1.70:Transferred entry 7.2.4.5		0,408144121
IVV	Propionic Ac [g/L]	3.2.1.55:Non-reducing end alpha-L-arabinofuranosidase		0,408099481
IVV	Acetic Ac [g/L]	2.3.1.180:Beta-ketoacyl-[acyl-carrier-protein] synthase III		0,406149896
IVT	Propionic Ac [g/L]	4.1.1.32:Phosphoenolpyruvate carboxykinase (GTP)		0,405900511
IVT	Propionic Ac [g/L]	5.99.1.2:Transferred entry 5.6.2.1		0,405393112
IVV	Butyric Ac [g/L]	3.1.11.6:Exodeoxyribonuclease VII		0,403189695
IVV	Butyric Ac [g/L]	4.3.3.7:4-hydroxy-tetrahydrodipicolinate synthase		0,401616207
IVV	Propionic Ac [g/L]	2.6.1.83:LL-diaminopimelate aminotransferase		0,400546197
IVT	Acetic Ac [g/L]	2.3.1.234:N(6)-L-threonylcarbamoyladenine synthase		0,398955892
IVT	Acetic Ac [g/L]	3.1.3.10:Glucose-1-phosphatase		0,397990297
IVV	Acetic Ac [g/L]	3.4.22.40:Bleomycin hydrolase		0,397831554
IVT	Acetic Ac [g/L]	2.3.2.6:Lysine/arginine leucyltransferase		0,393365811
IVT	Acetic Ac [g/L]	3.5.4.43:Hydroxydechloroatrazine ethylaminohydrolase		0,392547294
IVT	Propionic Ac [g/L]	2.8.1.6:Biotin synthase		0,391205518
IVT	Propionic Ac [g/L]	1.8.4.8:Phosphoadenylyl-sulfate reductase (thioredoxin)		0,39030076
IVV	Propionic Ac [g/L]	1.1.1.37:Malate dehydrogenase		0,388529244
IVT	Acetic Ac [g/L]	2.7.7.38:3-deoxy-manno-octulosonate cytidylyltransferase		0,387952462
IVV	Acetic Ac [g/L]	2.3.1.79:Maltose O-acetyltransferase		0,387793169
IVT	Acetic Ac [g/L]	2.7.3.9:Phosphoenolpyruvate--protein phosphotransferase		0,387532506
IVT	Propionic Ac [g/L]	1.1.1.23:Histidinol dehydrogenase		0,387395994
IVV	Propionic Ac [g/L]	4.2.1.11:Phosphopyruvate hydratase		0,385939508
IVV	Propionic Ac [g/L]	3.5.1.88:Peptide deformylase		0,385768554
IVV	Butyric Ac [g/L]	3.6.1.66:XTP/dITP diphosphatase		0,38539391
IVV	Acetic Ac [g/L]	2.5.1.7:UDP-N-acetylglucosamine 1-carboxyvinyltransferase		0,385350842
IVV	Acetic Ac [g/L]	3.4.25.2:HslU--HslV peptidase		0,385341667
IVV	Propionic Ac [g/L]	6.1.1.17:Glutamate--tRNA ligase		0,384825168
IVT	Propionic Ac [g/L]	2.5.1.129:Flavin prenyltransferase		0,383828858
IVT	Acetic Ac [g/L]	2.1.1.13:Methionine synthase		0,382812288
IVV	Butyric Ac [g/L]	6.3.4.5:Argininosuccinate synthase		0,381620328
IVT	Acetic Ac [g/L]	1.1.1.38:Malate dehydrogenase (oxaloacetate-decarboxylating)		0,380865185
IVV	Butyric Ac [g/L]	2.7.1.40:Pyruvate kinase		0,378180995
IVV	Propionic Ac [g/L]	2.1.1.207:tRNA (cytidine(34)-2'-O)-methyltransferase		0,37675708
IVT	Acetic Ac [g/L]	2.7.7.41:Phosphatidate cytidylyltransferase		0,375452618
IVT	Acetic Ac [g/L]	1.1.1.23:Histidinol dehydrogenase		0,375255915
IVT	Propionic Ac [g/L]	3.5.1.5:Urease		0,374998436
IVV	Acetic Ac [g/L]	1.1.1.40:Malate dehydrogenase (oxaloacetate-decarboxylating) (NADP(+))		0,374929067
IVT	Acetic Ac [g/L]	3.4.21.107:Peptidase Do		0,374911355
IVV	Propionic Ac [g/L]	5.99.1.2:Transferred entry 5.6.2.1		0,374006615
IVV	Butyric Ac [g/L]	6.3.2.10:UDP-N-acetylmuramoyl-tripeptide--D-alanyl-D-alanine ligase		0,373325266
IVT	Propionic Ac [g/L]	1.7.99.1:Hydroxylamine reductase		0,373287452
IVT	Acetic Ac [g/L]	3.5.1.16:Acetylornithine deacetylase		0,372713668
IVV	Propionic Ac [g/L]	6.3.2.8:UDP-N-acetylmuramate--L-alanine ligase		0,372407981
IVT	Propionic Ac [g/L]	6.1.1.5:Isoleucine--tRNA ligase		0,367137194
IVV	Propionic Ac [g/L]	3.1.3.2:Acid phosphatase		0,367087693
IVT	Propionic Ac [g/L]	1.2.1.38:N-acetyl-gamma-glutamyl-phosphate reductase		0,366820195
IVV	Propionic Ac [g/L]	1.6.5.11:NADH dehydrogenase (quinone)		0,365954696
IVV	Propionic Ac [g/L]	6.3.4.13:Phosphoribosylamine--glycine ligase		0,364296015
IVV	Propionic Ac [g/L]	6.3.4.3:Formate--tetrahydrofolate ligase		0,36345851
IVT	Propionic Ac [g/L]	2.7.1.5:Rhamnulokinase		0,363080218
IVV	Acetic Ac [g/L]	1.18.6.1:Nitrogenase		0,362252502
IVV	Acetic Ac [g/L]	1.3.1.6:Fumarate reductase (NADH)		0,362252502
IVV	Acetic Ac [g/L]	1.3.1.91:tRNA-dihydrouridine(20) synthase (NAD(P)(+))		0,362252502
IVV	Acetic Ac [g/L]	2.3.3.16:Citrate synthase (unknown stereospecificity)		0,362252502
IVV	Acetic Ac [g/L]	2.7.9.2:Pyruvate, water dikinase		0,362252502
IVV	Acetic Ac [g/L]	2.8.1.6:Biotin synthase		0,362252502
IVV	Acetic Ac [g/L]	2.8.4.1:Coenzyme-B sulfoethylthiotransferase		0,362252502
IVV	Acetic Ac [g/L]	3.2.1.89:Arabinogalactan endo-beta-1,4-galactanase		0,362252502
IVV	Acetic Ac [g/L]	4.2.1.24:Porphobilinogen synthase		0,362252502
IVV	Acetic Ac [g/L]	4.3.99.3:7-carboxy-7-deazaguanine synthase		0,362252502

IVV	Acetic Ac [g/L]	5.4.3.8:Glutamate-1-semialdehyde 2,1-aminomutase	0,362252502
IVV	Acetic Ac [g/L]	6.1.1.23:Aspartate--tRNA(Asn) ligase	0,362252502
IVV	Acetic Ac [g/L]	6.5.1.1:DNA ligase (ATP)	0,362252502
IVV	Acetic Ac [g/L]	1.16.3.1:Ferroxidase	0,362252502
IVV	Acetic Ac [g/L]	1.4.1.2:Glutamate dehydrogenase	0,362252502
IVV	Acetic Ac [g/L]	1.5.98.2:5,10-methylenetetrahydromethanopterin reductase	0,362252502
IVV	Acetic Ac [g/L]	1.9.6.1:Nitrate reductase (cytochrome)	0,362252502
IVV	Acetic Ac [g/L]	2.1.1.206:tRNA (cytidine(56)-2'-O)-methyltransferase	0,362252502
IVV	Acetic Ac [g/L]	2.3.1.222:Phosphate propanoyltransferase	0,362252502
IVV	Acetic Ac [g/L]	2.3.3.1:Citrate (Si)-synthase	0,362252502
IVV	Acetic Ac [g/L]	2.5.1.61:Hydroxymethylbilane synthase	0,362252502
IVV	Acetic Ac [g/L]	2.6.1.37:2-aminoethylphosphonate--pyruvate transaminase	0,362252502
IVV	Acetic Ac [g/L]	2.7.1.17:Xylulokinase	0,362252502
IVV	Acetic Ac [g/L]	2.7.11.32:[Pyruvate, phosphate dikinase] kinase	0,362252502
IVV	Acetic Ac [g/L]	2.7.4.27:([Pyruvate, phosphate dikinase] phosphate) phosphotransferase	0,362252502
IVV	Acetic Ac [g/L]	3.2.1.131:Xylan alpha-1,2-glucuronosidase	0,362252502
IVV	Acetic Ac [g/L]	3.2.1.41:Pullulanase	0,362252502
IVV	Acetic Ac [g/L]	3.4.14.12:Xaa-Xaa-Pro tripeptidyl-peptidase	0,362252502
IVV	Acetic Ac [g/L]	3.4.25.1:Proteasome endopeptidase complex	0,362252502
IVV	Acetic Ac [g/L]	3.5.4.5:Cytidine deaminase	0,362252502
IVV	Acetic Ac [g/L]	3.6.1.8:ATP diphosphatase	0,362252502
IVV	Acetic Ac [g/L]	4.2.1.32:L(+)-tartrate dehydratase	0,362252502
IVV	Acetic Ac [g/L]	4.2.1.70:Pseudouridylate synthase	0,362252502
IVV	Acetic Ac [g/L]	4.3.1.19:Threonine ammonia-lyase	0,362252502
IVV	Acetic Ac [g/L]	5.1.3.23:UDP-2,3-diacetamido-2,3-dideoxyglucuronic acid 2-epimerase	0,362252502
IVV	Acetic Ac [g/L]	6.2.1.14:6-carboxyhexanoate--CoA ligase	0,362252502
IVV	Acetic Ac [g/L]	6.3.4.19:tRNA(Ile)-lysidine synthetase	0,362252502
IVV	Acetic Ac [g/L]	6.4.1.1:Pyruvate carboxylase	0,362252502
IVV	Acetic Ac [g/L]	1.3.5.1:Succinate dehydrogenase (quinone)	0,362252502
IVV	Acetic Ac [g/L]	2.3.1.n3:Transferred entry 2.3.1.275	0,362252502
IVV	Acetic Ac [g/L]	2.3.2.6:Lysine/arginine leucyltransferase	0,362252502
IVV	Acetic Ac [g/L]	2.4.2.3:Uridine phosphorylase	0,362252502
IVV	Acetic Ac [g/L]	1.1.5.3:Glycerol-3-phosphate dehydrogenase	0,362252502
IVV	Acetic Ac [g/L]	2.1.1.133:Precorrin-4 C(11)-methyltransferase	0,362252502
IVV	Acetic Ac [g/L]	2.1.1.166:23S rRNA (uridine(2552)-2'-O)-methyltransferase	0,362252502
IVV	Acetic Ac [g/L]	2.4.2.15:Guanosine phosphorylase	0,362252502
IVV	Acetic Ac [g/L]	2.4.2.2:Pyrimidine-nucleoside phosphorylase	0,362252502
IVV	Acetic Ac [g/L]	2.4.2.4:Thymidine phosphorylase	0,362252502
IVV	Acetic Ac [g/L]	2.4.2.48:tRNA-guanine(15) transglycosylase	0,362252502
IVV	Propionic Ac [g/L]	2.8.1.7:Cysteine desulfurase	0,361823634
IVT	Acetic Ac [g/L]	2.3.1.129:Acyl-[acyl-carrier-protein]--UDP-N-acetylglucosamine O-acyltransferase	0,360631623
IVT	Propionic Ac [g/L]	6.3.2.6:Phosphoribosylaminoimidazolesuccinocarboxamide synthase	0,358212931
IVT	Acetic Ac [g/L]	6.3.2.9:UDP-N-acetylmuramoyl-L-alanine--D-glutamate ligase	0,357927845
IVV	Propionic Ac [g/L]	5.5.1.4:Inositol-3-phosphate synthase	0,357386635
IVV	Propionic Ac [g/L]	3.2.1.78:Mannan endo-1,4-beta-mannosidase	0,357024663
IVV	Propionic Ac [g/L]	3.2.1.17:Lysozyme	0,354573329
IVT	Acetic Ac [g/L]	3.1.3.18:Phosphoglycolate phosphatase	0,353894758
IVV	Butyric Ac [g/L]	2.7.1.50:Hydroxyethylthiazole kinase	0,351990551
IVT	Propionic Ac [g/L]	5.3.1.1:Triose-phosphate isomerase	0,351924587
IVV	Propionic Ac [g/L]	3.1.3.25:Inositol-phosphate phosphatase	0,351873748
IVT	Acetic Ac [g/L]	3.5.4.1:Cytosine deaminase	0,350966467
IVT	Propionic Ac [g/L]	6.3.3.2:5-formyltetrahydrofolate cyclo-ligase	0,348982218
IVT	Acetic Ac [g/L]	3.2.1.135:Neopullulanase	0,348939454
IVV	Propionic Ac [g/L]	2.1.1.74:(NAD(P)H-oxidizing)	0,348472896
IVT	Propionic Ac [g/L]	1.5.1.20:Methylenetetrahydrofolate reductase (NAD(P)H)	0,347792006
IVT	Acetic Ac [g/L]	5.3.1.14:L-rhamnose isomerase	0,34776039
IVV	Propionic Ac [g/L]	1.7.99.1:Hydroxylamine reductase	0,346454232
IVT	Propionic Ac [g/L]	2.3.3.13:2-isopropylmalate synthase	0,346348215
IVV	Butyric Ac [g/L]	1.17.99.6:Epoxyqueuosine reductase	0,345128833
IVT	Propionic Ac [g/L]	2.7.1.24:Dephospho-CoA kinase	0,344937081
IVT	Propionic Ac [g/L]	2.3.1.15:Glycerol-3-phosphate 1-O-acyltransferase	0,34386388
IVT	Acetic Ac [g/L]	2.4.1.21:Starch synthase	0,343480568
IVT	Propionic Ac [g/L]	6.3.4.14:Biotin carboxylase	0,34313883
IVT	Acetic Ac [g/L]	3.2.1.4:Cellulase	0,342260606
IVT	Propionic Ac [g/L]	2.7.9.1:Pyruvate, phosphate dikinase	0,340922324
IVV	Butyric Ac [g/L]	2.7.2.1:Acetate kinase	0,340731484
IVV	Butyric Ac [g/L]	4.6.1.12:2-C-methyl-D-erythritol 2,4-cyclodiphosphate synthase	0,339229081
IVT	Propionic Ac [g/L]	1.8.4.11:Peptide-methionine (S)-S-oxide reductase	0,339019568
IVV	Acetic Ac [g/L]	6.3.5.1:NAD(+) synthase (glutamine-hydrolyzing)	0,338806757
IVT	Acetic Ac [g/L]	2.3.2.2:Gamma-glutamyltransferase	0,33838262
IVV	Acetic Ac [g/L]	1.3.1.12:Prephenate dehydrogenase	0,338292215
IVT	Acetic Ac [g/L]	3.5.3.6:Arginine deiminase	0,337302299
IVV	Acetic Ac [g/L]	5.3.1.4:L-arabinose isomerase	0,33684459
IVT	Acetic Ac [g/L]	2.1.1.223:tRNA(1)(Val) (adenine(37)-N(6))-methyltransferase	0,336077447
IVT	Propionic Ac [g/L]	2.5.1.3:Thiamine phosphate synthase	0,33599903
IVT	Acetic Ac [g/L]	6.3.2.5:Phosphopantothenate--cysteine ligase (CTP)	0,334594058
IVV	Butyric Ac [g/L]	1.17.1.8:4-hydroxy-tetrahydrodipicolinate reductase	0,334036283
IVV	Propionic Ac [g/L]	1.1.1.23:Histidinol dehydrogenase	0,333948773
IVT	Propionic Ac [g/L]	3.5.4.19:Phosphoribosyl-AMP cyclohydrolase	0,332391623
IVV	Propionic Ac [g/L]	2.7.7.27:Glucose-1-phosphate adenylyltransferase	0,332250638
IVV	Butyric Ac [g/L]	2.4.1.182:Lipid-A-disaccharide synthase	0,332206487
IVV	Acetic Ac [g/L]	1.2.1.12:Glyceraldehyde-3-phosphate dehydrogenase (phosphorylating)	0,332204495
IVV	Propionic Ac [g/L]	4.1.2.19:Rhamnulose-1-phosphate aldolase	0,331920778

IVT	Acetic Ac [g/L]	5.4.4.2:Isochorismate synthase	0,331616424
IVV	Butyric Ac [g/L]	2.7.1.35:Pyridoxal kinase	0,330657113
IVV	Propionic Ac [g/L]	1.1.1.86:Ketol-acid reductoisomerase (NADP(+))	0,328880266
IVT	Propionic Ac [g/L]	3.6.3.31:Transferred entry 7.6.2.11	0,328379402
IVT	Propionic Ac [g/L]	2.8.1.7:Cysteine desulfurase	0,327792968
IVV	Butyric Ac [g/L]	2.3.2.3:Lysyltransferase	0,326970742
IVV	Butyric Ac [g/L]	2.7.7.27:Glucose-1-phosphate adenylyltransferase	0,326377147
IVV	Propionic Ac [g/L]	2.3.2.3:Lysyltransferase	0,32560043
IVV	Propionic Ac [g/L]	1.6.99.5:Transferred entry 1.6.5.11	0,325216229
IVV	Butyric Ac [g/L]	2.1.1.74:(NAD(P)H-oxidizing)	0,324784206
IVV	Butyric Ac [g/L]	3.1.3.25:Inositol-phosphate phosphatase	0,324423673
IVV	Butyric Ac [g/L]	3.2.1.78:Mannan endo-1,4-beta-mannosidase	0,323859725
IVV	Propionic Ac [g/L]	4.6.1.12:2-C-methyl-D-erythritol 2,4-cyclodiphosphate synthase	0,322848098
IVV	Butyric Ac [g/L]	1.1.1.127:2-dehydro-3-deoxy-D-gluconate 5-dehydrogenase	0,322549725
IVV	Butyric Ac [g/L]	3.4.14.5:Dipeptidyl-peptidase IV	0,321718155
IVV	Butyric Ac [g/L]	1.1.1.37:Malate dehydrogenase	0,319925704
IVT	Acetic Ac [g/L]	2.6.1.11:Acetylornithine transaminase	0,317417007
IVV	Butyric Ac [g/L]	4.2.1.8:Mannonate dehydratase	0,317385105
IVV	Propionic Ac [g/L]	6.1.1.2:Tryptophan--tRNA ligase	0,317272444
IVV	Butyric Ac [g/L]	4.1.1.70:Transferred entry 7.2.4.5	0,317034054
IVV	Propionic Ac [g/L]	2.7.7.38:3-deoxy-manno-octulosonate cytidylyltransferase	0,314799814
IVT	Acetic Ac [g/L]	2.3.1.35:Glutamate N-acetyltransferase	0,313513688
IVV	Butyric Ac [g/L]	1.3.1.9:Enoyl-[acyl-carrier-protein] reductase (NADH)	0,313264144
IVV	Butyric Ac [g/L]	2.6.1.87:UDP-4-amino-4-deoxy-L-arabinose aminotransferase	0,313264144
IVV	Butyric Ac [g/L]	1.3.1.1:Dihydrouracil dehydrogenase (NAD(+))	0,313264144
IVV	Butyric Ac [g/L]	1.8.4.8:Phosphoadenylyl-sulfate reductase (thioredoxin)	0,313264144
IVV	Butyric Ac [g/L]	2.5.1.31:diphosphate specific)	0,313264144
IVV	Butyric Ac [g/L]	3.2.1.52:Beta-N-acetylhexosaminidase	0,313264144
IVV	Butyric Ac [g/L]	3.4.24.70:Oligopeptidase A	0,313264144
IVV	Butyric Ac [g/L]	1.1.1.135:GDP-6-deoxy-D-talose 4-dehydrogenase	0,313264144
IVV	Butyric Ac [g/L]	1.1.1.38:Malate dehydrogenase (oxaloacetate-decarboxylating)	0,313264144
IVV	Butyric Ac [g/L]	1.12.7.2:Ferredoxin hydrogenase	0,313264144
IVV	Butyric Ac [g/L]	1.2.1.27:Methylmalonate-semialdehyde dehydrogenase (CoA acylating)	0,313264144
IVV	Butyric Ac [g/L]	1.3.1.28:2,3-dihydro-2,3-dihydroxybenzoate dehydrogenase	0,313264144
IVV	Butyric Ac [g/L]	1.6.1.2:NAD(P)(+) transhydrogenase (Re/Si-specific)	0,313264144
IVV	Butyric Ac [g/L]	1.7.1.7:GMP reductase	0,313264144
IVV	Butyric Ac [g/L]	2.1.1.131:Precorrin-3B C(17)-methyltransferase	0,313264144
IVV	Butyric Ac [g/L]	2.1.1.185:23S rRNA (guanosine(2251)-2'-O)-methyltransferase	0,313264144
IVV	Butyric Ac [g/L]	2.1.1.197:Malonyl-[acyl-carrier protein] O-methyltransferase	0,313264144
IVV	Butyric Ac [g/L]	2.3.1.157:Glucosamine-1-phosphate N-acetyltransferase	0,313264144
IVV	Butyric Ac [g/L]	2.3.1.16:Acetyl-CoA C-acyltransferase	0,313264144
IVV	Butyric Ac [g/L]	2.3.1.181:Lipoyl(octanoyl) transferase	0,313264144
IVV	Butyric Ac [g/L]	2.5.1.18:Glutathione transferase	0,313264144
IVV	Butyric Ac [g/L]	2.6.1.13:Ornithine aminotransferase	0,313264144
IVV	Butyric Ac [g/L]	2.7.7.23:UDP-N-acetylglucosamine diphosphorylase	0,313264144
IVV	Butyric Ac [g/L]	2.8.1.4:tRNA uracil 4-sulfurtransferase	0,313264144
IVV	Butyric Ac [g/L]	3.1.1.5:Lysophospholipase	0,313264144
IVV	Butyric Ac [g/L]	3.1.21.4:Type II site-specific deoxyribonuclease	0,313264144
IVV	Butyric Ac [g/L]	3.1.3.23:Sugar-phosphatase	0,313264144
IVV	Butyric Ac [g/L]	3.1.4.52:Cyclic-guanylate-specific phosphodiesterase	0,313264144
IVV	Butyric Ac [g/L]	3.2.1.51:Alpha-L-fucosidase	0,313264144
IVV	Butyric Ac [g/L]	3.4.21.107:Peptidase Do	0,313264144
IVV	Butyric Ac [g/L]	3.4.24.75:Lysostaphin	0,313264144
IVV	Butyric Ac [g/L]	3.5.1.18:Succinyl-diaminopimelate desuccinylase	0,313264144
IVV	Butyric Ac [g/L]	3.5.99.2:Aminopyrimidine aminohydrolase	0,313264144
IVV	Butyric Ac [g/L]	3.5.99.5:2-aminomuconate deaminase	0,313264144
IVV	Butyric Ac [g/L]	3.6.3.2:Transferred entry 7.2.2.14	0,313264144
IVV	Butyric Ac [g/L]	3.6.3.3:Transferred entry 7.2.2.21	0,313264144
IVV	Butyric Ac [g/L]	3.7.1.20:3-fumarylpyruvate hydrolase	0,313264144
IVV	Butyric Ac [g/L]	3.7.1.5:Acylpyruvate hydrolase	0,313264144
IVV	Butyric Ac [g/L]	4.1.1.86:Diaminobutyrate decarboxylase	0,313264144
IVV	Butyric Ac [g/L]	4.4.1.5:Lactoylglutathione lyase	0,313264144
IVV	Butyric Ac [g/L]	5.4.1.2:Transferred entry 5.4.99.61	0,313264144
IVV	Butyric Ac [g/L]	6.3.2.2:Glutamate--cysteine ligase	0,313264144
IVV	Butyric Ac [g/L]	7.3.2.1:ABC-type phosphate transporter	0,313264144
IVV	Butyric Ac [g/L]	2.1.1.222:2-polyprenyl-6-hydroxyphenol methylase	0,313264144
IVV	Butyric Ac [g/L]	2.1.1.64:3-demethylubiquinol 3-O-methyltransferase	0,313264144
IVV	Butyric Ac [g/L]	2.5.1.54:3-deoxy-7-phosphoheptulonate synthase	0,313264144
IVV	Butyric Ac [g/L]	5.1.99.1:Methylmalonyl-CoA epimerase	0,313264144
IVV	Butyric Ac [g/L]	5.3.3.10:5-carboxymethyl-2-hydroxymuconate Delta-isomerase	0,313264144
IVV	Propionic Ac [g/L]	1.5.1.5:Methylenetetrahydrofolate dehydrogenase (NADP(+))	0,313057814
IVV	Acetic Ac [g/L]	6.3.4.14:Biotin carboxylase	0,310997005
IVV	Propionic Ac [g/L]	1.1.1.95:Phosphoglycerate dehydrogenase	0,310889509
IVT	Propionic Ac [g/L]	3.4.13.22:D-Ala-D-Ala dipeptidase	0,31083602
IVT	Acetic Ac [g/L]	2.7.7.27:Glucose-1-phosphate adenylyltransferase	0,310512575
IVT	Acetic Ac [g/L]	3.4.16.4:Serine-type D-Ala-D-Ala carboxypeptidase	0,309925478
IVV	Acetic Ac [g/L]	1.1.1.85:3-isopropylmalate dehydrogenase	0,309217032
IVV	Propionic Ac [g/L]	6.3.1.2:Glutamine synthetase	0,307463052
IVT	Propionic Ac [g/L]	5.1.3.20:ADP-glyceromanno-heptose 6-epimerase	0,306633037
IVV	Propionic Ac [g/L]	4.2.1.10:3-dehydroquinate dehydratase	0,305996761
IVV	Propionic Ac [g/L]	5.3.1.9:Glucose-6-phosphate isomerase	0,3058522
IVT	Acetic Ac [g/L]	2.7.1.30:Glycerol kinase	0,305255527
IVV	Butyric Ac [g/L]	2.3.1.n2:Transferred entry 2.3.1.274	0,304624537

IVT	Acetic Ac [g/L]	2.6.1.9:Histidinol-phosphate transaminase		0,303838838
IVT	Acetic Ac [g/L]	3.2.1.99:Arabinan endo-1,5-alpha-L-arabinosidase		0,30337578
IVV	Propionic Ac [g/L]	5.1.3.1:Ribulose-phosphate 3-epimerase		0,302999856
IVV	Butyric Ac [g/L]	2.5.1.74:1,4-dihydroxy-2-naphthoate polyprenyltransferase		0,302518738
IVT	Acetic Ac [g/L]	4.1.3.27:Anthranilate synthase		0,301004384
IVT	Acetic Ac [g/L]	5.4.99.2:Methylmalonyl-CoA mutase		0,299876119
IVV	Propionic Ac [g/L]	2.4.2.10:Orotate phosphoribosyltransferase		0,299630305
IVV	Butyric Ac [g/L]	2.4.2.19:Nicotinate-nucleotide diphosphorylase (carboxylating)		0,298952281
IVV	Butyric Ac [g/L]	1.11.1.15:Peroxiredoxin		0,298520322
IVT	Acetic Ac [g/L]	2.5.1.15:Dihydropteroate synthase		0,296807008
IVV	Propionic Ac [g/L]	6.1.1.4:Leucine--tRNA ligase		0,294469767
IVT	Propionic Ac [g/L]	2.7.8.26:Adenosylcobinamide-GDP ribazoletransferase		0,293226227
IVT	Acetic Ac [g/L]	3.2.1.52:Beta-N-acetylhexosaminidase		0,293106077
IVT	Propionic Ac [g/L]	2.5.1.16:Spermidine synthase		0,292817213
IVT	Propionic Ac [g/L]	1.14.13.81:Magnesium-protoporphyrin IX monomethyl ester (oxidative) cyclase		0,289634784
IVV	Propionic Ac [g/L]	6.1.1.19:Arginine--tRNA ligase		0,287658337
IVV	Butyric Ac [g/L]	6.1.1.21:Histidine--tRNA ligase		0,287450172
IVT	Propionic Ac [g/L]	2.7.2.8:Acetylglutamate kinase		0,287385371
IVT	Acetic Ac [g/L]	2.3.1.9:Acetyl-CoA C-acetyltransferase		0,287080459
IVT	Acetic Ac [g/L]	2.5.1.49:O-acetylhomoserine aminocarboxypropyltransferase		0,285093778
IVV	Propionic Ac [g/L]	4.1.1.23:Orotidine-5'-phosphate decarboxylase		0,284995448
IVT	Propionic Ac [g/L]	6.3.4.15:Biotin--[biotin carboxyl-carrier protein] ligase		0,284651426
IVV	Acetic Ac [g/L]	1.5.1.3:Dihydrofolate reductase		0,284478355
IVV	Butyric Ac [g/L]	4.1.3.36:1,4-dihydroxy-2-naphthoyl-CoA synthase		0,28415266
IVV	Acetic Ac [g/L]	2.1.3.9:N-acetylornithine carbamoyltransferase		0,283574862
IVV	Propionic Ac [g/L]	2.7.6.1:Ribose-phosphate diphosphokinase		0,282111439
IVV	Propionic Ac [g/L]	2.1.1.45:Thymidylate synthase		0,282036822
IVT	Acetic Ac [g/L]	2.7.6.5:GTP diphosphokinase		0,281091873
IVT	Acetic Ac [g/L]	4.1.2.13:Fructose-bisphosphate aldolase		0,28069219
IVV	Propionic Ac [g/L]	1.1.1.267:1-deoxy-D-xylulose-5-phosphate reductoisomerase		0,27980591
IVT	Acetic Ac [g/L]	4.2.1.1:Carbonic anhydrase		0,278650488
IVV	Butyric Ac [g/L]	2.7.1.130:Tetraacyldisaccharide 4'-kinase		0,278055333
IVT	Propionic Ac [g/L]	5.5.1.4:Inositol-3-phosphate synthase		0,278053803
IVV	Propionic Ac [g/L]	1.17.7.4:4-hydroxy-3-methylbut-2-enyl diphosphate reductase		0,2780489
IVV	Butyric Ac [g/L]	6.1.1.20:Phenylalanine--tRNA ligase		0,27708762
IVV	Butyric Ac [g/L]	3.2.1.183:UDP-N-acetylglucosamine 2-epimerase (hydrolyzing)		0,276997163
IVT	Propionic Ac [g/L]	1.1.1.42:Isocitrate dehydrogenase (NADP(+))		0,276983114
IVV	Butyric Ac [g/L]	3.1.26.4:Ribonuclease H		0,276077893
IVV	Acetic Ac [g/L]	5.2.1.8:Peptidylprolyl isomerase		0,275920407
IVV	Propionic Ac [g/L]	6.3.5.5:Carbamoyl-phosphate synthase (glutamine-hydrolyzing)		0,275547241
IVT	Acetic Ac [g/L]	2.4.1.227:acetylglucosaminyltransferase		0,275284441
IVT	Propionic Ac [g/L]	3.1.3.1:Alkaline phosphatase		0,274546662
IVT	Propionic Ac [g/L]	1.3.1.6:Fumarate reductase (NADH)		0,273241959
IVT	Acetic Ac [g/L]	3.2.1.122:Maltose-6'-phosphate glucosidase		0,273228527
IVV	Propionic Ac [g/L]	3.1.26.11:Ribonuclease Z		0,273050825
IVT	Acetic Ac [g/L]	3.6.5.3:Protein-synthesizing GTPase		0,27223432
IVV	Propionic Ac [g/L]	2.7.2.11:Glutamate 5-kinase		0,272094621
IVV	Butyric Ac [g/L]	2.6.1.16:Glutamine--fructose-6-phosphate transaminase (isomerizing)		0,270922976
IVT	Propionic Ac [g/L]	6.1.1.3:Threonine--tRNA ligase		0,269543236
IVT	Propionic Ac [g/L]	2.4.2.7:Adenine phosphoribosyltransferase		0,269258084
IVV	Propionic Ac [g/L]	2.7.9.1:Pyruvate, phosphate dikinase		0,268816515
IVV	Butyric Ac [g/L]	2.3.1.28:Chloramphenicol O-acetyltransferase		0,268442881
IVV	Acetic Ac [g/L]	2.7.1.23:NAD(+) kinase		0,267710567
IVT	Propionic Ac [g/L]	4.1.1.48:Indole-3-glycerol-phosphate synthase		0,26750251
IVT	Propionic Ac [g/L]	2.1.3.2:Aspartate carbamoyltransferase		0,266786661
IVV	Butyric Ac [g/L]	6.3.4.21:Nicotinate phosphoribosyltransferase		0,266682461
IVT	Acetic Ac [g/L]	6.1.1.18:Glutamine--tRNA ligase		0,264879117
IVT	Acetic Ac [g/L]	4.2.1.2:Fumarate hydratase		0,264152031
IVV	Acetic Ac [g/L]	3.5.99.6:Glucosamine-6-phosphate deaminase		0,263986058
IVT	Acetic Ac [g/L]	4.1.1.36:Phosphopantothenoylcysteine decarboxylase		0,263864111
IVV	Acetic Ac [g/L]	2.3.1.31:Homoserine O-acetyltransferase		0,262935967
IVV	Butyric Ac [g/L]	2.7.1.156:Adenosylcobinamide kinase		0,262749381
IVV	Butyric Ac [g/L]	2.7.7.62:Adenosylcobinamide-phosphate guanylyltransferase		0,262749381
IVV	Butyric Ac [g/L]	6.3.4.20:7-cyano-7-deazaguanine synthase		0,260807499
IVT	Acetic Ac [g/L]	3.4.22.40:Bleomycin hydrolase		0,260385245
IVT	Acetic Ac [g/L]	1.7.99.4:Nitrate reductase		0,260280551
IVV	Acetic Ac [g/L]	6.3.2.1:Pantoate--beta-alanine ligase (AMP-forming)		0,259175501
IVV	Propionic Ac [g/L]	4.2.1.75:Uroporphyrinogen-III synthase		0,25632626
IVT	Propionic Ac [g/L]	1.1.1.27:L-lactate dehydrogenase		0,255562856
IVV	Butyric Ac [g/L]	1.1.1.133:dTDP-4-dehydrorhamnose reductase		0,25505306
IVV	Propionic Ac [g/L]	1.3.99.1:Deleted entry		0,254701661
IVV	Acetic Ac [g/L]	2.4.2.22:Xanthine phosphoribosyltransferase		0,254442031
IVV	Butyric Ac [g/L]	3.5.1.108:UDP-3-O-acyl-N-acetylglucosamine deacetylase		0,253304793
IVV	Butyric Ac [g/L]	1.1.1.44:Phosphogluconate dehydrogenase (NADP(+)-dependent, decarboxylating)		0,251033459
IVV	Acetic Ac [g/L]	2.2.1.9:synthase		0,250993218
IVV	Acetic Ac [g/L]	5.4.2.11:Phosphoglycerate mutase (2,3-diphosphoglycerate-dependent)		0,250763516
IVT	Acetic Ac [g/L]	2.5.1.75:tRNA dimethylallyltransferase		0,247649235
IVV	Butyric Ac [g/L]	2.2.1.2:Transaldolase		0,24688305
IVV	Acetic Ac [g/L]	1.5.1.7:Saccharopine dehydrogenase (NAD(+), L-lysine-forming)		0,246460835
IVT	Propionic Ac [g/L]	2.7.1.17:Xylulokinase		0,246312641
IVT	Acetic Ac [g/L]	3.1.26.11:Ribonuclease Z		0,245164851
IVV	Propionic Ac [g/L]	3.1.3.3:Phosphoserine phosphatase		0,245120706
IVV	Propionic Ac [g/L]	5.4.99.2:Methylmalonyl-CoA mutase		0,244871256

IVV	Butyric Ac [g/L]	1.8.1.9:Thioredoxin-disulfide reductase	0,244829509
IVV	Butyric Ac [g/L]	2.7.4.16:Thiamine-phosphate kinase	0,242457928
IVV	Acetic Ac [g/L]	2.3.1.39:[Acyl-carrier-protein] S-malonyltransferase	0,24244169
IVV	Propionic Ac [g/L]	2.7.1.40:Pyruvate kinase	0,241992283
IVV	Acetic Ac [g/L]	2.1.1.45:Thymidylate synthase	0,241486054
IVV	Propionic Ac [g/L]	1.5.1.2:Pyrroline-5-carboxylate reductase	0,241254146
IVV	Propionic Ac [g/L]	2.3.1.180:Beta-ketoacyl-[acyl-carrier-protein] synthase III	0,240113167
IVV	Propionic Ac [g/L]	1.5.1.20:Methylenetetrahydrofolate reductase (NAD(P)H)	0,238335435
IVT	Propionic Ac [g/L]	1.1.1.133:dTDP-4-dehydrorhamnose reductase	0,236831317
IVV	Butyric Ac [g/L]	2.7.4.1:Polyphosphate kinase	0,23608603
IVV	Propionic Ac [g/L]	6.3.5.4:Asparagine synthase (glutamine-hydrolyzing)	0,235745921
IVT	Propionic Ac [g/L]	2.7.1.191:Protein-N(pi)-phosphohistidine--D-mannose phosphotransferase	0,233961587
IVV	Butyric Ac [g/L]	6.3.5.5:Carbamoyl-phosphate synthase (glutamine-hydrolyzing)	0,233861395
IVV	Butyric Ac [g/L]	2.7.7.87:L-threonylcarbamoyladenylate synthase	0,233524248
IVV	Butyric Ac [g/L]	1.17.1.9:Formate dehydrogenase	0,233053566
IVT	Propionic Ac [g/L]	1.3.1.9:Enoyl-[acyl-carrier-protein] reductase (NADH)	0,231247533
IVV	Propionic Ac [g/L]	3.1.21.3:Type I site-specific deoxyribonuclease	0,229204932
IVT	Acetic Ac [g/L]	3.1.1.11:Pectinesterase	0,229147248
IVT	Propionic Ac [g/L]	3.5.2.3:Dihydroorotase	0,228869583
IVT	Propionic Ac [g/L]	3.4.11.18:Methionyl aminopeptidase	0,227480093
IVT	Acetic Ac [g/L]	2.4.1.18:1,4-alpha-glucan branching enzyme	0,227280549
IVT	Acetic Ac [g/L]	5.1.1.1:Alanine racemase	0,225099351
IVV	Butyric Ac [g/L]	1.2.1.41:Glutamate-5-semialdehyde dehydrogenase	0,224552682
IVT	Propionic Ac [g/L]	2.4.2.18:Anthranilate phosphoribosyltransferase	0,224494066
IVT	Propionic Ac [g/L]	6.1.1.10:Methionine--tRNA ligase	0,222072195
IVV	Acetic Ac [g/L]	2.4.99.17:S-adenosylmethionine:tRNA ribosyltransferase-isomerase	0,222018701
IVT	Propionic Ac [g/L]	2.4.2.17:ATP phosphoribosyltransferase	0,220434495
IVT	Acetic Ac [g/L]	1.7.1.7:GMP reductase	0,219815249
IVV	Butyric Ac [g/L]	1.1.1.23:Histidinol dehydrogenase	0,219754281
IVT	Propionic Ac [g/L]	2.1.1.207:tRNA (cytidine(34)-2'-O)-methyltransferase	0,219598079
IVV	Propionic Ac [g/L]	2.4.1.227:acetylglucosaminyltransferase	0,219434596
IVT	Propionic Ac [g/L]	6.3.4.19:tRNA(Ile)-lysidine synthetase	0,218799977
IVT	Acetic Ac [g/L]	2.1.1.74:(NAD(P)H-oxidizing)	0,217561037
IVT	Acetic Ac [g/L]	2.7.1.71:Shikimate kinase	0,216991086
IVT	Acetic Ac [g/L]	4.3.1.1:Aspartate ammonia-lyase	0,216271035
IVV	Propionic Ac [g/L]	4.2.1.59:3-hydroxyacyl-[acyl-carrier-protein] dehydratase	0,216068896
IVV	Propionic Ac [g/L]	2.3.1.54:Formate C-acetyltransferase	0,215947677
IVV	Propionic Ac [g/L]	4.1.1.32:Phosphoenolpyruvate carboxykinase (GTP)	0,215181667
IVV	Propionic Ac [g/L]	4.2.99.18:DNA-(apurinic or apyrimidinic site) lyase	0,214539483
IVT	Acetic Ac [g/L]	6.1.1.14:Glycine--tRNA ligase	0,213633719
IVV	Propionic Ac [g/L]	2.4.1.182:Lipid-A-disaccharide synthase	0,21306497
IVV	Acetic Ac [g/L]	4.1.2.19:Rhamnulose-1-phosphate aldolase	0,212970374
IVT	Acetic Ac [g/L]	6.4.1.1:Pyruvate carboxylase	0,211653284
IVT	Acetic Ac [g/L]	1.17.1.8:4-hydroxy-tetrahydrodipicolinate reductase	0,211597706
IVV	Butyric Ac [g/L]	2.5.1.9:Riboflavin synthase	0,210206369
IVT	Acetic Ac [g/L]	1.1.1.22:UDP-glucose 6-dehydrogenase	0,209110015
IVT	Acetic Ac [g/L]	4.1.1.49:Phosphoenolpyruvate carboxykinase (ATP)	0,208537722
IVV	Butyric Ac [g/L]	2.1.1.13:Methionine synthase	0,208533542
IVT	Acetic Ac [g/L]	2.3.1.179:Beta-ketoacyl-[acyl-carrier-protein] synthase II	0,208411772
IVT	Propionic Ac [g/L]	4.2.1.33:3-isopropylmalate dehydratase	0,207563899
IVT	Propionic Ac [g/L]	5.4.3.8:Glutamate-1-semialdehyde 2,1-aminomutase	0,206597336
IVT	Propionic Ac [g/L]	6.3.4.13:Phosphoribosylamine--glycine ligase	0,206145158
IVV	Butyric Ac [g/L]	2.2.1.6:Acetolactate synthase	0,205993638
IVV	Butyric Ac [g/L]	2.7.1.39:Homoserine kinase	0,20424671
IVV	Propionic Ac [g/L]	3.5.99.6:Glucosamine-6-phosphate deaminase	0,201951905
IVV	Propionic Ac [g/L]	4.2.3.1:Threonine synthase	0,200834098
IVV	Acetic Ac [g/L]	3.5.2.3:Dihydroorotase	0,200671744
IVT	Acetic Ac [g/L]	2.7.4.16:Thiamine-phosphate kinase	0,199837872
IVT	Propionic Ac [g/L]	1.6.5.11:NADH dehydrogenase (quinone)	0,199654024
IVT	Propionic Ac [g/L]	1.2.1.72:Erythrose-4-phosphate dehydrogenase	0,199651462
IVV	Butyric Ac [g/L]	3.1.3.15:Histidinol-phosphatase	0,198871782
IVV	Propionic Ac [g/L]	2.1.1.177:23S rRNA (pseudouridine(1915)-N(3))-methyltransferase	0,198516356
IVV	Propionic Ac [g/L]	2.3.1.1:Amino-acid N-acetyltransferase	0,198385416
IVV	Propionic Ac [g/L]	1.3.5.4:Fumarate reductase (quinol)	0,198385416
IVV	Propionic Ac [g/L]	2.1.1.34:tRNA (guanosine(18)-2'-O)-methyltransferase	0,198385416
IVV	Propionic Ac [g/L]	3.2.1.14:Chitinase	0,198385416
IVV	Propionic Ac [g/L]	3.6.1.7:Acylphosphatase	0,198385416
IVV	Propionic Ac [g/L]	2.6.1.39:2-aminoadipate transaminase	0,198385416
IVV	Propionic Ac [g/L]	3.1.1.72:Acetylxylan esterase	0,198385416
IVV	Propionic Ac [g/L]	3.2.1.26:Beta-fructofuranosidase	0,198385416
IVV	Propionic Ac [g/L]	3.4.15.5:Peptidyl-dipeptidase Dcp	0,198385416
IVV	Propionic Ac [g/L]	4.6.1.1:Adenylate cyclase	0,198385416
IVV	Propionic Ac [g/L]	5.1.3.20:ADP-glyceromanno-heptose 6-epimerase	0,198385416
IVV	Propionic Ac [g/L]	6.3.4.15:Biotin--[biotin carboxyl-carrier protein] ligase	0,198385416
IVV	Propionic Ac [g/L]	2.6.1.62:Adenosylmethionine--8-amino-7-oxononanoate transaminase	0,198385416
IVT	Propionic Ac [g/L]	6.2.1.30:Phenylacetate--CoA ligase	0,198069964
IVT	Propionic Ac [g/L]	2.1.1.176:16S rRNA (cytosine(967)-C(5))-methyltransferase	0,196447606
IVT	Acetic Ac [g/L]	2.7.9.2:Pyruvate, water dikinase	0,19581236
IVV	Acetic Ac [g/L]	3.1.22.4:Crossover junction endodeoxyribonuclease	0,195531021
IVV	Butyric Ac [g/L]	3.1.13.1:Exoribonuclease II	0,193892883
IVV	Propionic Ac [g/L]	2.5.1.15:Dihydropteroate synthase	0,192833792
IVV	Propionic Ac [g/L]	3.4.21.88:Repressor LexA	0,192264032
IVT	Propionic Ac [g/L]	2.1.3.9:N-acetylornithine carbamoyltransferase	0,192142706

IVV	Propionic Ac [g/L]	1.1.1.42:Isocitrate dehydrogenase (NADP(+))		0,191081078
IVV	Acetic Ac [g/L]	4.2.1.45:CDP-glucose 4,6-dehydratase		0,190919599
IVV	Propionic Ac [g/L]	2.7.1.92:5-dehydro-2-deoxygluconokinase		0,187480763
IVV	Propionic Ac [g/L]	2.8.1.13:tRNA-uridine 2-sulfurtransferase		0,187058715
IVV	Propionic Ac [g/L]	6.1.1.20:Phenylalanine--tRNA ligase		0,187055989
IVT	Propionic Ac [g/L]	6.3.2.9:UDP-N-acetylmuramoyl-L-alanine--D-glutamate ligase		0,186948337
IVT	Acetic Ac [g/L]	3.5.1.2:Glutaminase		0,185825301
IVT	Acetic Ac [g/L]	2.7.1.21:Thymidine kinase		0,184995932
IVV	Butyric Ac [g/L]	2.7.7.60:2-C-methyl-D-erythritol 4-phosphate cytidylyltransferase		0,184876098
IVV	Propionic Ac [g/L]	2.7.2.7:Butyrate kinase		0,184090364
IVV	Propionic Ac [g/L]	2.3.1.30:Serine O-acetyltransferase		0,183689782
IVT	Propionic Ac [g/L]	2.5.1.54:3-deoxy-7-phosphoheptulonate synthase		0,182852469
IVV	Propionic Ac [g/L]	1.15.1.1:Superoxide dismutase		0,182825317
IVV	Propionic Ac [g/L]	5.3.1.24:Phosphoribosylanthranilate isomerase		0,182491326
IVT	Acetic Ac [g/L]	1.5.1.7:Saccharopine dehydrogenase (NAD(+), L-lysine-forming)		0,180031698
IVV	Propionic Ac [g/L]	1.1.1.218:Morphine 6-dehydrogenase		0,17958866
IVT	Acetic Ac [g/L]	6.3.4.20:7-cyano-7-deazaguanine synthase		0,178796465
IVV	Propionic Ac [g/L]	4.1.1.19:Arginine decarboxylase		0,176831013
IVT	Propionic Ac [g/L]	2.3.1.157:Glucosamine-1-phosphate N-acetyltransferase		0,176336969
IVV	Acetic Ac [g/L]	2.4.2.14:Amidophosphoribosyltransferase		0,174224372
IVT	Propionic Ac [g/L]	2.7.11.32:[Pyruvate, phosphate dikinase] kinase		0,173603108
IVT	Propionic Ac [g/L]	2.7.4.27:([Pyruvate, phosphate dikinase] phosphate) phosphotransferase		0,173603108
IVV	Propionic Ac [g/L]	3.5.4.16:GTP cyclohydrolase I		0,172113055
IVT	Acetic Ac [g/L]	5.4.2.12:Phosphoglycerate mutase (2,3-diphosphoglycerate-independent)		0,171114555
IVV	Butyric Ac [g/L]	1.1.1.100:3-oxoacyl-[acyl-carrier-protein] reductase		0,170098048
IVT	Acetic Ac [g/L]	2.8.4.4:[Ribosomal protein S12] (aspartate(89)-C(3))-methylthiotransferase		0,169930897
IVT	Propionic Ac [g/L]	1.3.99.1:Deleted entry		0,169729069
IVT	Acetic Ac [g/L]	4.1.99.17:Phosphomethylpyrimidine synthase		0,169391405
IVV	Propionic Ac [g/L]	4.2.3.3:Methylglyoxal synthase		0,16930208
IVT	Acetic Ac [g/L]	6.1.1.23:Aspartate--tRNA(Asn) ligase		0,16908268
IVT	Acetic Ac [g/L]	2.7.2.8:Acetylglutamate kinase		0,165351104
IVT	Propionic Ac [g/L]	1.2.7.3:2-oxoglutarate synthase		0,164229291
IVV	Butyric Ac [g/L]	2.7.3.9:Phosphoenolpyruvate--protein phosphotransferase		0,164045014
IVV	Propionic Ac [g/L]	2.5.1.19:3-phosphoshikimate 1-carboxyvinyltransferase		0,160534764
IVT	Acetic Ac [g/L]	6.3.4.3:Formate--tetrahydrofolate ligase		0,160321869
IVV	Acetic Ac [g/L]	3.5.1.28:N-acetylmuramoyl-L-alanine amidase		0,158139498
IVV	Propionic Ac [g/L]	6.3.4.14:Biotin carboxylase		0,158135725
IVT	Acetic Ac [g/L]	1.14.14.9:4-hydroxyphenylacetate 3-monooxygenase		0,157723931
IVV	Acetic Ac [g/L]	2.3.1.8:Phosphate acetyltransferase		0,157647592
IVT	Acetic Ac [g/L]	4.2.1.20:Tryptophan synthase		0,15744752
IVT	Acetic Ac [g/L]	2.7.1.48:Uridine kinase		0,157175408
IVV	Butyric Ac [g/L]	2.4.2.29:tRNA-guanine(34) transglycosylase		0,156996888
IVV	Butyric Ac [g/L]	1.3.99.1:Deleted entry		0,155938685
IVT	Acetic Ac [g/L]	3.1.11.2:Exodeoxyribonuclease III		0,153851231
IVT	Acetic Ac [g/L]	2.7.1.180:FAD:protein FMN transferase		0,153599057
IVV	Butyric Ac [g/L]	2.7.7.18:Nicotinate-nucleotide adenylyltransferase		0,150518593
IVV	Propionic Ac [g/L]	4.1.1.3:Transferred entry 4.1.1.112		0,149228215
IVV	Propionic Ac [g/L]	2.1.1.192:23S rRNA (adenine(2503)-C(2))-methyltransferase		0,148184483
IVT	Acetic Ac [g/L]	6.5.1.2:DNA ligase (NAD(+))		0,14660165
IVT	Acetic Ac [g/L]	5.1.3.14:UDP-N-acetylglucosamine 2-epimerase (non-hydrolyzing)		0,145136722
IVT	Acetic Ac [g/L]	2.8.1.10:Thiazole synthase		0,139843524
IVT	Propionic Ac [g/L]	4.1.1.11:Aspartate 1-decarboxylase		0,139031734
IVV	Propionic Ac [g/L]	1.11.1.1:NADH peroxidase		0,137968921
IVV	Butyric Ac [g/L]	1.6.5.11:NADH dehydrogenase (quinone)		0,137014068
IVT	Propionic Ac [g/L]	1.1.1.274:2,5-didehydrogluconate reductase (2-dehydro-D-gluconate-forming)		0,135952186
IVT	Acetic Ac [g/L]	1.2.1.12:Glyceraldehyde-3-phosphate dehydrogenase (phosphorylating)		0,135132533
IVT	Acetic Ac [g/L]	6.1.1.2:Tryptophan--tRNA ligase		0,134569736
IVT	Acetic Ac [g/L]	1.17.1.9:Formate dehydrogenase		0,133956225
IVV	Acetic Ac [g/L]	1.17.4.1:Ribonucleoside-diphosphate reductase		0,133725986
IVV	Acetic Ac [g/L]	5.1.3.13:dTDP-4-dehydrorhamnose 3,5-epimerase		0,131865209
IVV	Acetic Ac [g/L]	3.4.11.4:Tripeptide aminopeptidase		0,131703124
IVV	Propionic Ac [g/L]	6.3.3.1:Phosphoribosylformylglycinamidine cyclo-ligase		0,131030658
IVT	Acetic Ac [g/L]	1.1.1.49:Glucose-6-phosphate dehydrogenase (NADP(+))		0,130435817
IVV	Propionic Ac [g/L]	6.3.2.10:UDP-N-acetylmuramoyl-tripeptide--D-alanyl-D-alanine ligase		0,129668993
IVT	Propionic Ac [g/L]	6.3.4.2:CTP synthase (glutamine hydrolyzing)		0,128857981
IVV	Butyric Ac [g/L]	2.1.1.37:DNA (cytosine-5-)-methyltransferase		0,128835694
IVT	Acetic Ac [g/L]	4.2.1.10:3-dehydroquinate dehydratase		0,127713951
IVV	Propionic Ac [g/L]	2.7.1.35:Pyridoxal kinase		0,126631358
IVT	Propionic Ac [g/L]	2.4.1.1:Glycogen phosphorylase		0,126573334
IVT	Propionic Ac [g/L]	4.4.1.11:Methionine gamma-lyase		0,126303135
IVT	Acetic Ac [g/L]	4.4.1.11:Methionine gamma-lyase		0,125894623
IVV	Butyric Ac [g/L]	1.17.4.1:Ribonucleoside-diphosphate reductase		0,124584214
IVT	Propionic Ac [g/L]	1.1.1.100:3-oxoacyl-[acyl-carrier-protein] reductase		0,124542628
IVV	Propionic Ac [g/L]	1.97.1.4:[Formate-C-acetyltransferase]-activating enzyme		0,123164671
IVV	Propionic Ac [g/L]	3.4.22.40:Bleomycin hydrolase		0,123048451
IVT	Acetic Ac [g/L]	4.2.1.11:Phosphopyruvate hydratase		0,12126589
IVV	Acetic Ac [g/L]	3.1.26.4:Ribonuclease H		0,12106606
IVV	Propionic Ac [g/L]	2.1.1.13:Methionine synthase		0,119779865
IVT	Propionic Ac [g/L]	1.1.1.58:Tagaturonate reductase		0,119002872
IVT	Acetic Ac [g/L]	3.4.15.5:Peptidyl-dipeptidase Dcp		0,118786841
IVT	Acetic Ac [g/L]	3.6.1.23:dUTP diphosphatase		0,116050504
IVV	Butyric Ac [g/L]	2.1.1.228:tRNA (guanine(37)-N(1))-methyltransferase		0,115397141
IVV	Acetic Ac [g/L]	1.1.1.25:Shikimate dehydrogenase		0,114746595

IVT	Propionic Ac [g/L]	2.7.7.23:UDP-N-acetylglucosamine diphosphorylase	0,113615713
IVV	Propionic Ac [g/L]	2.7.4.16:Thiamine-phosphate kinase	0,113531946
IVV	Acetic Ac [g/L]	3.1.3.2:Acid phosphatase	0,113370152
IVT	Acetic Ac [g/L]	4.4.1.21:S-ribosylhomocysteine lyase	0,11271797
IVT	Acetic Ac [g/L]	2.1.1.198:16S rRNA (cytidine(1402)-2'-O)-methyltransferase	0,112505894
IVV	Propionic Ac [g/L]	4.2.1.9:Dihydroxy-acid dehydratase	0,111454659
IVV	Acetic Ac [g/L]	4.1.1.23:Orotidine-5'-phosphate decarboxylase	0,108595225
IVV	Butyric Ac [g/L]	6.3.4.3:Formate--tetrahydrofolate ligase	0,107524932
IVT	Propionic Ac [g/L]	2.7.7.4:Sulfate adenylyltransferase	0,107065487
IVV	Propionic Ac [g/L]	6.3.3.2:5-formyltetrahydrofolate cyclo-ligase	0,106991138
IVT	Acetic Ac [g/L]	3.2.1.55:Non-reducing end alpha-L-arabinofuranosidase	0,106815669
IVV	Butyric Ac [g/L]	4.3.3.6:Pyridoxal 5'-phosphate synthase (glutamine hydrolyzing)	0,105640911
IVV	Propionic Ac [g/L]	4.2.1.1:Carbonic anhydrase	0,105396074
IVV	Propionic Ac [g/L]	3.2.2.n1:Cytokinin riboside 5'-monophosphate phosphoribohydrolase	0,105264317
IVT	Acetic Ac [g/L]	1.11.1.15:Peroxiredoxin	0,103798549
IVV	Propionic Ac [g/L]	1.1.1.69:Gluconate 5-dehydrogenase	0,102008536
IVT	Acetic Ac [g/L]	4.1.1.70:Transferred entry 7.2.4.5	0,100417035
IVT	Acetic Ac [g/L]	1.2.1.41:Glutamate-5-semialdehyde dehydrogenase	0,098900847
IVV	Acetic Ac [g/L]	2.7.4.8:Guanylate kinase	0,092582408
IVV	Propionic Ac [g/L]	2.8.4.4:[Ribosomal protein S12] (aspartate(89)-C(3))-methylthiotransferase	0,092213783
IVV	Propionic Ac [g/L]	2.4.2.7:Adenine phosphoribosyltransferase	0,091914984
IVT	Acetic Ac [g/L]	3.2.1.37:Xylan 1,4-beta-xylosidase	0,091078808
IVT	Acetic Ac [g/L]	3.4.11.4:Tripeptide aminopeptidase	0,090867901
IVV	Acetic Ac [g/L]	2.7.1.71:Shikimate kinase	0,088739707
IVT	Acetic Ac [g/L]	3.1.3.45:3-deoxy-manno-octulosonate-8-phosphatase	0,088227116
IVV	Propionic Ac [g/L]	2.4.1.281:4-O-beta-D-mannosyl-D-glucose phosphorylase	0,08753851
IVV	Acetic Ac [g/L]	3.2.1.23:Beta-galactosidase	0,087513381
IVT	Propionic Ac [g/L]	2.7.1.11:6-phosphofructokinase	0,08717298
IVV	Butyric Ac [g/L]	2.5.1.75:tRNA dimethylallyltransferase	0,08523846
IVT	Acetic Ac [g/L]	3.1.26.5:Ribonuclease P	0,085179261
IVT	Acetic Ac [g/L]	2.7.4.3:Adenylate kinase	0,08432376
IVT	Acetic Ac [g/L]	2.4.2.17:ATP phosphoribosyltransferase	0,084230834
IVV	Acetic Ac [g/L]	3.4.11.5:Prolyl aminopeptidase	0,083416218
IVV	Butyric Ac [g/L]	5.1.3.32:L-rhamnose mutarotase	0,082862969
IVV	Acetic Ac [g/L]	5.4.99.25:tRNA pseudouridine(55) synthase	0,079926943
IVV	Acetic Ac [g/L]	2.7.7.18:Nicotinate-nucleotide adenylyltransferase	0,078782663
IVT	Propionic Ac [g/L]	4.1.2.25:Dihydroneopterin aldolase	0,078576153
IVT	Acetic Ac [g/L]	2.7.7.33:Glucose-1-phosphate cytidylyltransferase	0,078107876
IVT	Acetic Ac [g/L]	4.2.1.126:N-acetylmuramic acid 6-phosphate etherase	0,076568958
IVT	Acetic Ac [g/L]	3.6.3.12:Transferred entry 7.2.2.6	0,076478716
IVV	Butyric Ac [g/L]	2.4.1.129:Peptidoglycan glycosyltransferase	0,076187268
IVV	Butyric Ac [g/L]	1.1.1.267:1-deoxy-D-xylulose-5-phosphate reductoisomerase	0,075665462
IVV	Propionic Ac [g/L]	2.1.1.193:16S rRNA (uracil(1498)-N(3))-methyltransferase	0,074795459
IVT	Acetic Ac [g/L]	1.3.3.4:Protoporphyrinogen oxidase	0,074273455
IVT	Propionic Ac [g/L]	2.7.7.18:Nicotinate-nucleotide adenylyltransferase	0,073659354
IVV	Butyric Ac [g/L]	2.1.1.177:23S rRNA (pseudouridine(1915)-N(3))-methyltransferase	0,073384579
IVV	Acetic Ac [g/L]	2.4.1.227:acetylglucosaminyltransferase	0,072926248
IVT	Acetic Ac [g/L]	5.4.99.18:5-(carboxyamino)imidazole ribonucleotide mutase	0,067350449
IVV	Propionic Ac [g/L]	6.1.1.16:Cysteine--tRNA ligase	0,066637584
IVV	Propionic Ac [g/L]	3.2.1.1:Alpha-amylase	0,066483622
IVT	Acetic Ac [g/L]	2.7.1.40:Pyruvate kinase	0,064992757
IVV	Acetic Ac [g/L]	3.1.3.11:Fructose-bisphosphatase	0,063520616
IVT	Propionic Ac [g/L]	3.1.3.3:Phosphoserine phosphatase	0,063370624
IVT	Propionic Ac [g/L]	2.4.2.10:Orotate phosphoribosyltransferase	0,062250027
IVV	Propionic Ac [g/L]	2.4.1.129:Peptidoglycan glycosyltransferase	0,061295065
IVV	Butyric Ac [g/L]	2.7.4.6:Nucleoside-diphosphate kinase	0,060918098
IVV	Acetic Ac [g/L]	2.7.1.148:4-(cytidine 5'-diphospho)-2-C-methyl-D-erythritol kinase	0,060730802
IVV	Acetic Ac [g/L]	1.17.4.2:Ribonucleoside-triphosphate reductase (thioredoxin)	0,057955639
IVV	Butyric Ac [g/L]	2.1.1.198:16S rRNA (cytidine(1402)-2'-O)-methyltransferase	0,057461167
IVV	Acetic Ac [g/L]	4.1.1.19:Arginine decarboxylase	0,055733167
IVV	Acetic Ac [g/L]	2.7.1.21:Thymidine kinase	0,055540318
IVT	Propionic Ac [g/L]	4.2.1.11:Phosphopyruvate hydratase	0,0537138
IVT	Acetic Ac [g/L]	1.1.1.44:Phosphogluconate dehydrogenase (NADP(+)-dependent, decarboxylating)	0,053622921
IVV	Propionic Ac [g/L]	3.5.4.9:Methenyltetrahydrofolate cyclohydrolase	0,053204357
IVV	Propionic Ac [g/L]	4.3.3.6:Pyridoxal 5'-phosphate synthase (glutamine hydrolyzing)	0,053200872
IVT	Acetic Ac [g/L]	1.97.1.4:[Formate-C-acetyltransferase]-activating enzyme	0,052662176
IVV	Butyric Ac [g/L]	2.6.1.1:Aspartate transaminase	0,051971747
IVV	Butyric Ac [g/L]	2.1.1.192:23S rRNA (adenine(2503)-C(2))-methyltransferase	0,049824771
IVV	Propionic Ac [g/L]	3.5.2.6:Beta-lactamase	0,04939059
IVT	Propionic Ac [g/L]	6.2.1.5:Succinate--CoA ligase (ADP-forming)	0,048267488
IVT	Propionic Ac [g/L]	5.1.3.1:Ribulose-phosphate 3-epimerase	0,046893799
IVT	Propionic Ac [g/L]	2.3.2.3:Lysyltransferase	0,046093305
IVT	Propionic Ac [g/L]	2.7.1.48:Uridine kinase	0,04408321
IVV	Acetic Ac [g/L]	2.4.2.18:Anthranilate phosphoribosyltransferase	0,043980858
IVT	Propionic Ac [g/L]	3.6.3.14:Transferred entry 7.1.2.2	0,043702852
IVT	Acetic Ac [g/L]	3.5.4.3:Guanine deaminase	0,042901144
IVT	Propionic Ac [g/L]	2.8.1.4:tRNA uracil 4-sulfurtransferase	0,042884965
IVV	Butyric Ac [g/L]	1.1.1.69:Gluconate 5-dehydrogenase	0,042183746
IVV	Acetic Ac [g/L]	1.5.1.5:Methylenetetrahydrofolate dehydrogenase (NADP(+))	0,041511114
IVT	Acetic Ac [g/L]	1.1.1.193:5-amino-6-(5-phosphoribosylamino)uracil reductase	0,040662629
IVT	Acetic Ac [g/L]	3.5.4.26:Diaminohydroxyphosphoribosylaminopyrimidine deaminase	0,040662629
IVT	Acetic Ac [g/L]	3.2.2.n1:Cytokinin riboside 5'-monophosphate phosphoribohydrolase	0,03970314
IVT	Propionic Ac [g/L]	5.99.1.3:Transferred entry 5.6.2.2	0,039403002

IVT	Acetic Ac [g/L]	2.6.1.90:dTDP-3-amino-3,6-dideoxy-alpha-D-galactopyranose transaminase		0,038947401
IVV	Butyric Ac [g/L]	6.3.3.2:5-formyltetrahydrofolate cyclo-ligase		0,038777323
IVV	Butyric Ac [g/L]	6.1.1.7:Alanine--tRNA ligase		0,038093157
IVV	Propionic Ac [g/L]	6.1.1.7:Alanine--tRNA ligase		0,035750319
IVT	Acetic Ac [g/L]	4.3.1.17:L-serine ammonia-lyase		0,035584735
IVV	Butyric Ac [g/L]	3.1.26.3:Ribonuclease III		0,034408476
IVT	Propionic Ac [g/L]	2.3.1.51:1-acylglycerol-3-phosphate O-acyltransferase		0,033255188
IVV	Acetic Ac [g/L]	1.6.99.5:Transferred entry 1.6.5.11		0,032941959
IVV	Acetic Ac [g/L]	4.3.2.10:Imidazole glycerol-phosphate synthase		0,031893209
IVV	Acetic Ac [g/L]	6.1.1.5:Isoleucine--tRNA ligase		0,030529662
IVV	Propionic Ac [g/L]	2.4.2.29:tRNA-guanine(34) transglycosylase		0,029748361
IVV	Butyric Ac [g/L]	6.1.1.5:Isoleucine--tRNA ligase		0,029019811
IVT	Propionic Ac [g/L]	1.1.1.131:Mannuronate reductase		0,028595249
IVT	Propionic Ac [g/L]	2.6.1.76:Diaminobutyrate--2-oxoglutarate transaminase		0,028459716
IVT	Acetic Ac [g/L]	5.4.2.7:Phosphopentomutase		0,028046131
IVV	Butyric Ac [g/L]	3.6.3.15:Transferred entry 7.2.2.1		0,027895472
IVT	Propionic Ac [g/L]	2.1.1.34:tRNA (guanosine(18)-2'-O)-methyltransferase		0,026070375
IVV	Butyric Ac [g/L]	3.1.21.3:Type I site-specific deoxyribonuclease		0,0255578
IVT	Propionic Ac [g/L]	2.3.1.n2:Transferred entry 2.3.1.274		0,025140894
IVT	Acetic Ac [g/L]	2.5.1.55:3-deoxy-8-phosphooctulonate synthase		0,024621959
IVV	Butyric Ac [g/L]	5.4.2.11:Phosphoglycerate mutase (2,3-diphosphoglycerate-dependent)		0,024343459
IVV	Propionic Ac [g/L]	4.2.1.136:ADP-dependent NAD(P)H-hydrate dehydratase		0,023226126
IVV	Propionic Ac [g/L]	5.1.99.6:NAD(P)H-hydrate epimerase		0,023226126
IVT	Acetic Ac [g/L]	5.1.3.32:L-rhamnose mutarotase		0,022824321
IVV	Propionic Ac [g/L]	2.1.1.198:16S rRNA (cytidine(1402)-2'-O)-methyltransferase		0,022707117
IVV	Butyric Ac [g/L]	1.5.1.20:Methylenetetrahydrofolate reductase (NAD(P)H)		0,022294055
IVT	Acetic Ac [g/L]	2.4.1.182:Lipid-A-disaccharide synthase		0,021510238
IVT	Acetic Ac [g/L]	2.3.1.29:Glycine C-acetyltransferase		0,021264875
IVV	Propionic Ac [g/L]	4.1.2.13:Fructose-bisphosphate aldolase		0,02061171
IVT	Acetic Ac [g/L]	4.2.1.19:Imidazoleglycerol-phosphate dehydratase		0,020312364
IVV	Propionic Ac [g/L]	1.8.1.4:Dihydrolipoyl dehydrogenase		0,020120792
IVT	Propionic Ac [g/L]	1.8.5.3:Respiratory dimethylsulfoxide reductase		0,019854114
IVT	Acetic Ac [g/L]	3.1.3.5:5'-nucleotidase		0,019151893
IVV	Propionic Ac [g/L]	1.1.1.127:2-dehydro-3-deoxy-D-gluconate 5-dehydrogenase		0,018496527
IVV	Butyric Ac [g/L]	5.3.1.12:Glucuronate isomerase		0,018365786
IVT	Propionic Ac [g/L]	2.4.2.9:Uracil phosphoribosyltransferase		0,018343451
IVT	Propionic Ac [g/L]	2.7.6.3:2-amino-4-hydroxy-6-hydroxymethyldihydropteridine diphosphokinase		0,017731806
IVV	Butyric Ac [g/L]	2.7.7.72:CCA tRNA nucleotidyltransferase		0,017722382
IVT	Propionic Ac [g/L]	4.2.1.126:N-acetylmuramic acid 6-phosphate etherase		0,017721371
IVV	Butyric Ac [g/L]	1.1.1.3:Homoserine dehydrogenase		0,017436043
IVT	Propionic Ac [g/L]	5.3.1.8:Mannose-6-phosphate isomerase		0,017278789
IVT	Acetic Ac [g/L]	3.1.11.5:Exodeoxyribonuclease V		0,017068275
IVT	Acetic Ac [g/L]	3.1.22.4:Crossover junction endodeoxyribonuclease		0,016201445
IVV	Acetic Ac [g/L]	1.1.1.100:3-oxoacyl-[acyl-carrier-protein] reductase		0,015560803
IVV	Propionic Ac [g/L]	1.7.2.2:Nitrite reductase (cytochrome ammonia-forming)		0,014607816
IVV	Propionic Ac [g/L]	2.7.2.4:Aspartate kinase		0,014261992
IVT	Propionic Ac [g/L]	3.2.1.51:Alpha-L-fucosidase		0,012821667
IVT	Propionic Ac [g/L]	3.4.21.88:Repressor LexA		0,012555451
IVT	Acetic Ac [g/L]	2.1.1.37:DNA (cytosine-5-)-methyltransferase		0,012525543
IVV	Acetic Ac [g/L]	3.6.1.27:Undecaprenyl-diphosphate phosphatase		0,010248615
IVT	Propionic Ac [g/L]	3.5.4.25:GTP cyclohydrolase II		0,0102046
IVT	Acetic Ac [g/L]	5.4.99.27:tRNA pseudouridine(13) synthase		0,00967164
IVV	Butyric Ac [g/L]	3.2.1.4:Cellulase		0,008651591
IVV	Butyric Ac [g/L]	2.7.7.33:Glucose-1-phosphate cytidylyltransferase		0,008213007
IVT	Propionic Ac [g/L]	3.1.26.11:Ribonuclease Z		0,008152858
IVT	Acetic Ac [g/L]	3.4.21.88:Repressor LexA		0,008009368
IVV	Acetic Ac [g/L]	4.2.1.33:3-isopropylmalate dehydratase		0,006549018
IVV	Propionic Ac [g/L]	6.1.1.11:Serine--tRNA ligase		0,006459613
IVV	Butyric Ac [g/L]	2.8.1.7:Cysteine desulfurase		0,005050235
IVT	Acetic Ac [g/L]	3.4.21.92:Endopeptidase Clp		0,00487843
IVT	Acetic Ac [g/L]	3.5.4.33:tRNA(adenine(34)) deaminase		0,004784627
IVT	Acetic Ac [g/L]	4.1.1.5:Acetolactate decarboxylase		0,004528789
IVV	Butyric Ac [g/L]	2.3.1.129:Acyl-[acyl-carrier-protein]--UDP-N-acetylglucosamine O-acyltransferase		0,003986109
IVT	Acetic Ac [g/L]	3.4.25.2:HslU--HslV peptidase		0,003812912
IVT	Propionic Ac [g/L]	2.1.1.61:tRNA (5-methylaminomethyl-2-thiouridylate)-methyltransferase		0,003502309
IVT	Acetic Ac [g/L]	5.3.1.5:Xylose isomerase		0,002988092
IVV	Propionic Ac [g/L]	6.5.1.2:DNA ligase (NAD(+))		0,002457273
IVT	Propionic Ac [g/L]	4.2.3.1:Threonine synthase		0,001454033
IVV	Propionic Ac [g/L]	2.7.4.6:Nucleoside-diphosphate kinase		0,000704517
IVT	Propionic Ac [g/L]	1.1.1.79:Glyoxylate reductase (NADP(+))		0,000650507
IVV	Propionic Ac [g/L]	5.1.3.32:L-rhamnose mutarotase		0,000128263
